# SPACA9 and MNMIP1 bridge the seam of spermatid manchette microtubules

**DOI:** 10.1038/s44318-026-00833-w

**Published:** 2026-06-12

**Authors:** Jo H Judernatz, Svetlana Doroshev, Robin A Hoogebeen, Sven Jonkers, Donna Schweizer, Molly S C Gravett, Elizabeth G Bromfield, Stuart C Howes, Anna Akhmanova, Rui Zhang, Tzviya Zeev-Ben-Mordehai

**Affiliations:** 1 Bijvoet Centre for Biomolecular Research, Utrecht University, Utrecht, The Netherlands; 2 Cell Biology, Neurobiology and Biophysics, Department of Biology, Faculty of Science, Utrecht University, Utrecht, The Netherlands; 3 Bio21 Institute, School of BioSciences, Faculty of Science, University of Melbourne, Parkville, VIC Australia; 4 Department of Biochemistry and Molecular Biophysics, Washington University in St. Louis, School of Medicine, St. Louis, MO USA; 5 Present Address: Centre for Genomic Regulation (CRG), The Barcelona Institute of Science and Technology, Barcelona, Spain

**Keywords:** Cell Adhesion, Polarity & Cytoskeleton, Structural Biology

## Abstract

The manchette is a transient microtubule (MT)-based structure that is vital for the correct shaping of sperm during spermiogenesis. Throughout spermiogenesis, the manchette retains structural integrity for several days, raising the question of how its MTs are regulated. Here, using cryo-electron tomography of manchettes isolated from rat testes, we find that manchette MT ends are structurally diverse. We show that the MT-binding protein CLASP2 is present throughout the manchette and likely regulates both MT ends. Using cryo-electron microscopy single particle analysis and super-resolution microscopy, we reveal that SPACA9 and MNMIP1 (SH3D21) bind to the seam of manchette MTs from the luminal side. SPACA9 binds to both α- and β-tubulin of protofilament 1 but does not interact directly with protofilament 13, while MNMIP1 binds directly to protofilament 13. MNMIP1 further extends and threads through the MT lattice at the seam. Our study reveals a novel seam MT inner protein complex with a unique binding mode, providing a plausible explanation for MT regulation that maintains manchette integrity over an extended period.

## Synopsis

The spermatid manchette is a transient microtubule (MT) scaffold critical for proper sperm formation. This study uses cryo-ET and cryo-EM to reveal a protein complex formed by SPACA9 and MNMIP1 that bridges the seam of manchette MTs on the luminal side.

- Manchette MTs have blunt, flared, and tapered ends.
- SPACA9 and MNMIP1 form a bridge across the seam on the luminal side of manchette MTs.
- MNMIP1 extends and threads through the MT lattice at the seam.

## Abstract (web summary)

Cryo-EM and cryo-ET reveal a protein complex at the lumen of rat spermatid microtubules that binds the lattice at the seam.

## Graphical abstract

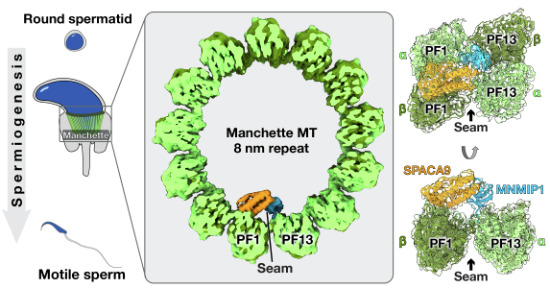

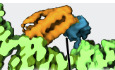

## Introduction

Cytosolic microtubules (MTs) exhibit dynamic instability, enabling them to rapidly alternate between growth and shrinkage phases within the cell (Mitchison and Kirschner, 1984; Desai and Mitchison, 1997; Heald and Nogales, 2002). This behavior is driven by the stochastic addition and loss of tubulin subunits, primarily at the plus end of the MT. Dynamic instability allows MTs to efficiently explore the intracellular environment, undergo remodeling in response to cellular cues, and fulfill essential roles in processes like mitosis, intracellular transport, and cell shape maintenance (Heald and Nogales, 2002).

MTs contain a structural discontinuity known as the “seam,” where the helical symmetry is disrupted. At the seam, lateral interactions between protofilaments (PFs) are heterotypic (α-β tubulin interactions) rather than homotypic (α–α tubulin or β–β tubulin interactions) as seen throughout the remainder of the MT lattice (Mandelkow et al, 1986; Kikkawa et al, 1994; Zhang and Nogales, 2015). The physiological significance of the seam is uncertain. The seam is likely a weak point in the structure of the MT and has thus been suggested to be important for the regulation of MT dynamics (LaFrance et al, 2022; Roostalu et al, 2020; Mandelkow et al, 1986; Zhang et al, 2015). In this sense, the seam is interesting as a specialized binding site for MT-associated proteins (MAPs) that can increase MT stability (des Georges et al, 2008; Mandelkow et al, 1986). However, while some seam-binding proteins have been discovered for axonemal MTs (Ma et al, 2019; Leung et al, 2023; Gui et al, 2022a; Legal et al, 2025), none have yet been conclusively identified for non-axonemal MTs.

MT inner proteins (MIPs) are a subset of MAPs that bind to the luminal surface of the MT lattice. The doublet MTs of axonemes and the triplet MTs of basal bodies and centrioles are often decorated by various MIPs (LeGuennec et al, 2021; Ma et al, 2019; Leung et al, 2023; Gui et al, 2022b). Recently, an intricate network of MIPs was found in the doublet MTs of mammalian sperm tails, where they are believed to contribute to doublet MT stabilization and regulation of flagellar motility (Leung et al, 2023; Ichikawa and Bui, 2018; Leung et al, 2021). In singlet MTs, MIPs were shown in the ventral disc MTs of *Giardia lamblia* (Schwartz et al, 2012), in the cortical MTs of the apicomplexan parasite *Toxoplasma gondii* (Wang et al, 2021), and in the sperm endpiece (Leung et al, 2023). In addition, mammalian neuronal MTs show a high concentration of luminal densities (Chakraborty et al, 2025), but to date, only two MIPs—MAP6 and α-tubulin acetyltransferase (αTAT)—have been unequivocally identified in mammalian non-axonemal MTs, where they promote MT stability, mechanical resilience, lattice integrity, and longevity under conditions of cold stress and pharmacological perturbation (Delphin et al, 2012; Cuveillier et al, 2020; Luo et al, 2025; Janke and Montagnac, 2017; Eshun-Wilson et al, 2019; Niu et al, 2023). However, the role of MIPs in non-axonemal MTs remains poorly understood, with only a few examples currently known.

**Figure 1:**
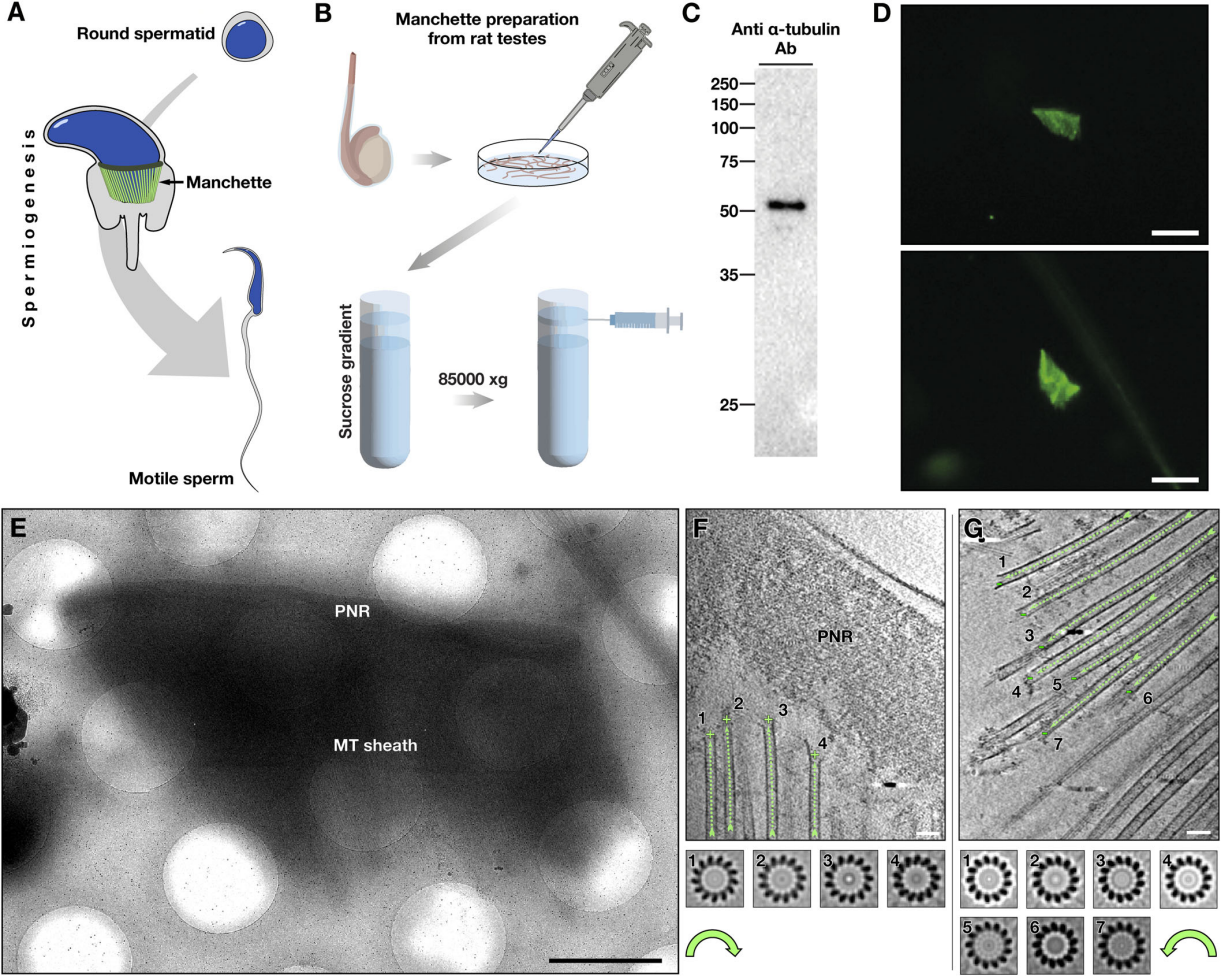
The manchette is made of 13 protofilament microtubules arranged unidirectionally. (**A**) Schematic illustration showing the position of the manchette in elongating rat spermatid. (**B**) Schematic diagram summarizing the main steps to isolate manchettes, including mechanical disruption of the seminiferous tubules in a microtubule-stabilizing buffer, followed by separation on a stepwise sucrose gradient. (**C**) Immunoblotting detection of α-tubulin in a manchette preparation. (**D**) Immunofluorescence using anti-α-tubulin antibodies shows manchettes with their characteristic trapezoid shape. Scale bars, 10 μm. (**E**) A cryo-EM projection image of an isolated manchette showing the perinuclear ring (PNR) and the microtubular (MT) sheath. Scale bar, 2 μm. (**F**) A tomographic slice of the manchette at the perinuclear ring region (upper panel) and rotational averages of the microtubules (lower panel). A clockwise protofilament slew indicates a view from minus to plus end direction. Arrows indicate the direction of the slew. Scale bar, 50 nm. (**G**) A tomographic slice of the manchette at the caudal end (upper panel) and rotational averages of the microtubules (lower panel). A counterclockwise protofilament slew indicates a view from plus to minus end direction. Arrows indicate the direction of the slew. Scale bar, 50 nm. Source data are available online for this figure.

A special subset of non-axonemal MTs appears during spermiogenesis and organizes into a scaffold around the spermatid’s nucleus, called the manchette (see Fig. 1A). Spermiogenesis defines the last step of spermatogenesis, where the round, haploid spermatids are structurally reorganized into elongated, motile  sperm cells (Hermo et al, 2010; Auger, 2018; O’Donnell, 2014). The manchette plays a crucial role in the morphological and functional transformations of spermatids during spermiogenesis (Rattner and Brinkley, 1972; Fouquet and Kann, 1994; Russell et al, 1991; Meistrich et al, 1990). Dysfunctions of the manchette, introduced either through chemical treatments or gene knockouts, have been reported to lead to deformed sperm heads (Dunleavy et al, 2021; Cheers et al, 2023; Hu et al, 2023; Russell et al, 1991; Meistrich et al, 1990). Hence, it was proposed that one of the main functions of the manchette is to aid in shaping and elongating the spermatid nucleus by exerting constriction forces (Meistrich et al, 1990). The correct morphology of sperm cells is an essential factor for male fertility (Oehninger and Kruger, 2021). Errors in sperm shaping lead to abnormal spermatozoa morphology, including teratozoospermia, which has been linked to male infertility due to an overall reduction of semen quality (Said et al, 2004; Kim, 2018; Agarwal et al, 2014).

The manchette emerges as a MT sheath around the distal part of the nucleus, after the nucleus has moved to the periphery of the spermatid and nuclear condensation has started (Lehti and Sironen, 2016; Moreno and Schatten, 2000). Throughout spermiogenesis, the manchette gradually moves towards the distal end of the nucleus, while its MTs extend and then disappear, commensurate with the completion of nuclear shaping (Lehti and Sironen, 2016; Rattner and Brinkley, 1972; Kierszenbaum and Tres, 2004). This process takes several days, in which the manchette and its MTs retain structural integrity, raising questions about their regulation (Dunleavy et al, 2019a; Lehti and Sironen, 2016; Mochida et al, 1998).

At the equatorial region of the spermatid’s nucleus, the upper border of the manchette is marked by the perinuclear ring, an amorphous protein matrix. The MT plus end tracking proteins ( + TIPs) end-binding protein 3 (EB3) and CAP-Gly domain-containing linker protein 170 (CLIP170) have been found to colocalize to the area of the perinuclear ring in mouse spermatids (Akhmanova et al, 2005; Lehti and Sironen, 2016), suggesting that MT plus ends face toward the perinuclear ring. The exact function and molecular composition of the perinuclear ring are not fully known, and structural information about the perinuclear ring and its interaction with the plus ends of manchette MTs is scarce (Rattner and Brinkley, 1972; Lehti and Sironen, 2016; Judernatz et al, 2025). The minus-end binding calmodulin-regulated spectrin-associated protein 1 (CAMSAP1) localizes to the caudal end of the manchette and was recently shown to play a role in manchette MT minus-end regulation (Hu et al, 2023). Knock-out of CAMSAP1 reduces the abundance of kinesin family member 2A (KIF2A) in manchette preparations and causes manchettes to elongate. It has been suggested that CAMSAP1 controls manchette MT length by recruiting KIF2A, which can depolymerize MTs.

Here, we combined cryo-electron microscopy (cryo-EM) and tomography (cryo-ET) with super-resolution microscopy to investigate structural factors that regulate manchette MTs. We find that manchette MTs have blunt, flared, and tapered ends. Furthermore, we find that manchette MTs are longitudinally compacted and slightly elliptical, and in their lumen, they are bridged at the seam by sperm acrosome-associated protein 9 (SPACA9) and manchette MT inner protein (MNMIP1; previously Src homology 3 domain-containing protein 21 (SH3D21)). Taken together, our data suggest two potential molecular mechanisms for manchette MT regulation, one at the ends and one along the lattice.

## Results

### Manchette MT ends are structurally variable

To image the manchette with cryo-EM, manchettes were isolated from rat testes in the presence of the MT-stabilizing reagent paclitaxel (Mochida et al, 1998) (Fig. 1B, see “Methods” for more details). Successful isolation was confirmed by Western blotting analysis and immunofluorescence staining (IF) using an anti-α-tubulin antibody (Fig. 1C,D). In IF, the isolated manchettes appeared as trapezoids, and cryo-EM further confirmed that the trapezoids are manchettes with the perinuclear ring on one side and numerous MTs forming the microtubular sheath on the other (Fig. 1E).

Based on localization experiments that found the +TIPs CLIP170 and EB3 at the equatorial region of the manchette (Akhmanova et al, 2005), MTs in the manchette are expected to be organized unidirectionally, with the plus ends embedded in the perinuclear ring. To confirm the MT polarity and PF number in our isolated manchette preps, we used cryo-ET and subtomogram averaging (Foster et al, 2022) (for details see “Methods”; Fig. 1F,G). Almost all manchette MTs measured (357/358 in 25 tomograms) comprised 13 PFs and had the same polarity as their neighbors, indicating tight regulation during nucleation and polymerization. 100% (102/102 in 8 tomograms) of MT plus ends faced toward the perinuclear ring (Fig. 1F). In contrast, at the caudal side of the manchette, 99% (138/139 in 7 tomograms) of the MT ends were minus ends (Fig. 1G). Notably, the MT plus ends were often not embedded in the dense matrix of the perinuclear ring (Fig. 1F).

**Figure 2:**
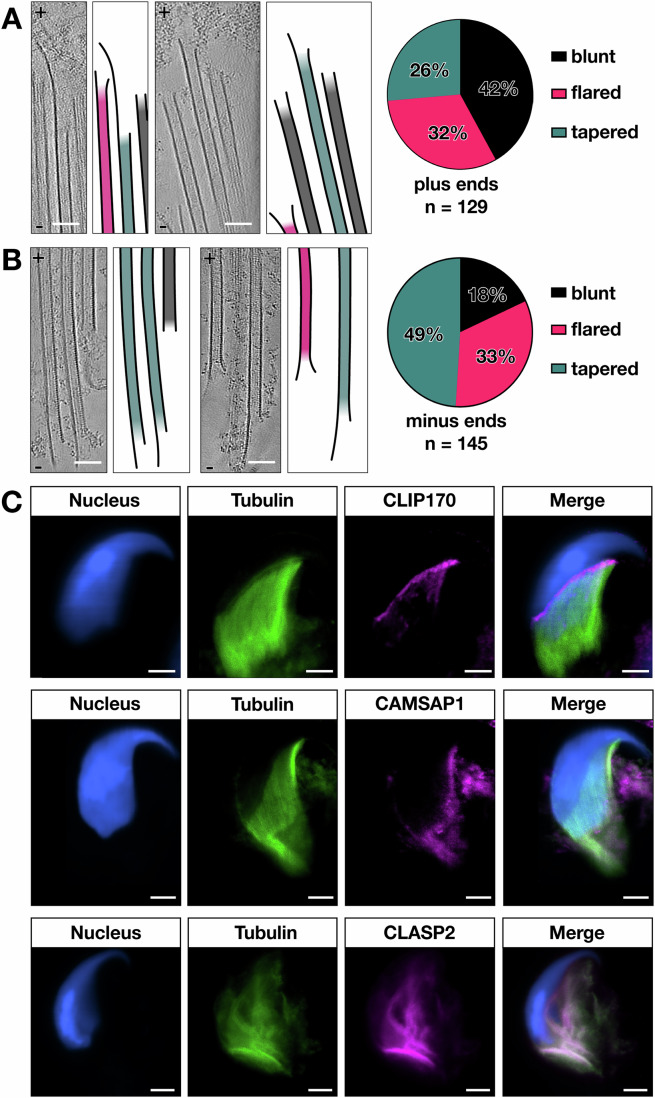
Manchette microtubule ends are structurally variable. (**A**) Tomographic slices with annotations and quantifications of microtubule plus ends showing blunt (dark gray) and flared ends (pink) as well as tapered ends with extended protofilaments (cyan). Scale bars, 50 nm. (**B**) Tomographic slices with annotations and quantifications of microtubule minus ends showing blunt (dark gray) and flared ends (pink) as well as tapered ends with extended protofilaments (cyan). Scale bars, 50 nm. (**C**) STED imaging of isolated spermatids with anti CLIP170, anti-CAMSAP1, anti-CLASP2, and anti α-tubulin antibodies. CLASP2 localizes to the entire manchette (lower panels), while the CLIP170 signal is concentrated at the equatorial region of the nucleus (upper panels), and CAMSAP1 is enriched at the distal ends of manchette microtubules (middle panels). Tubulin is green, and the nucleus is blue. Scale bars, 2 μm. Source data are available online for this figure.

Detailed 3D analysis of the manchette MT ends revealed structural variability. On both ends, we observed MTs with blunt, flared, and tapered ends with different frequencies (Fig. 2A,B, 10 tomograms for plus ends, 7 tomograms for minus ends). Most plus ends (42%) had blunt ends, while approximately a quarter (26%) had tapered ends with extending PFs (Fig. 2A). In contrast, almost half (49%) of the MT minus ends had tapered ends, and blunt ends were substantially less abundant (18%) (Fig. 2B). CLASP2 was previously shown to inhibit catastrophes at MT ends by stabilizing incomplete end structures (Aher et al, 2018; Lawrence and Zanic, 2019; Lawrence et al, 2018). These so-called CLASP2-like curved end structures highly resemble the tapered ends we observed for manchette MTs (Fig. 2A,B). To check CLASP2 localization in manchette MT, we used IF and stimulated emission depletion (STED) microscopy. While the +TIP CLIP170 clearly localized to the area of the perinuclear ring as previously reported (Akhmanova et al, 2005), the minus-end tracking protein (−TIP) CAMSAP1 localized more broadly across the manchette, indicating MTs in the manchette are highly variable in length (Fig. 2C; Movies EV1 and 2). In contrast, CLASP2 localized to the entire manchette (Fig. 2C; Movie EV3). The localization of CLASP2 to both ends of the manchette agrees with our observations of tapered MT ends with extending PFs on both sides of the manchette (Fig. 2A,B).

### MT inner proteins bind regularly to manchette MTs

**Figure 3:**
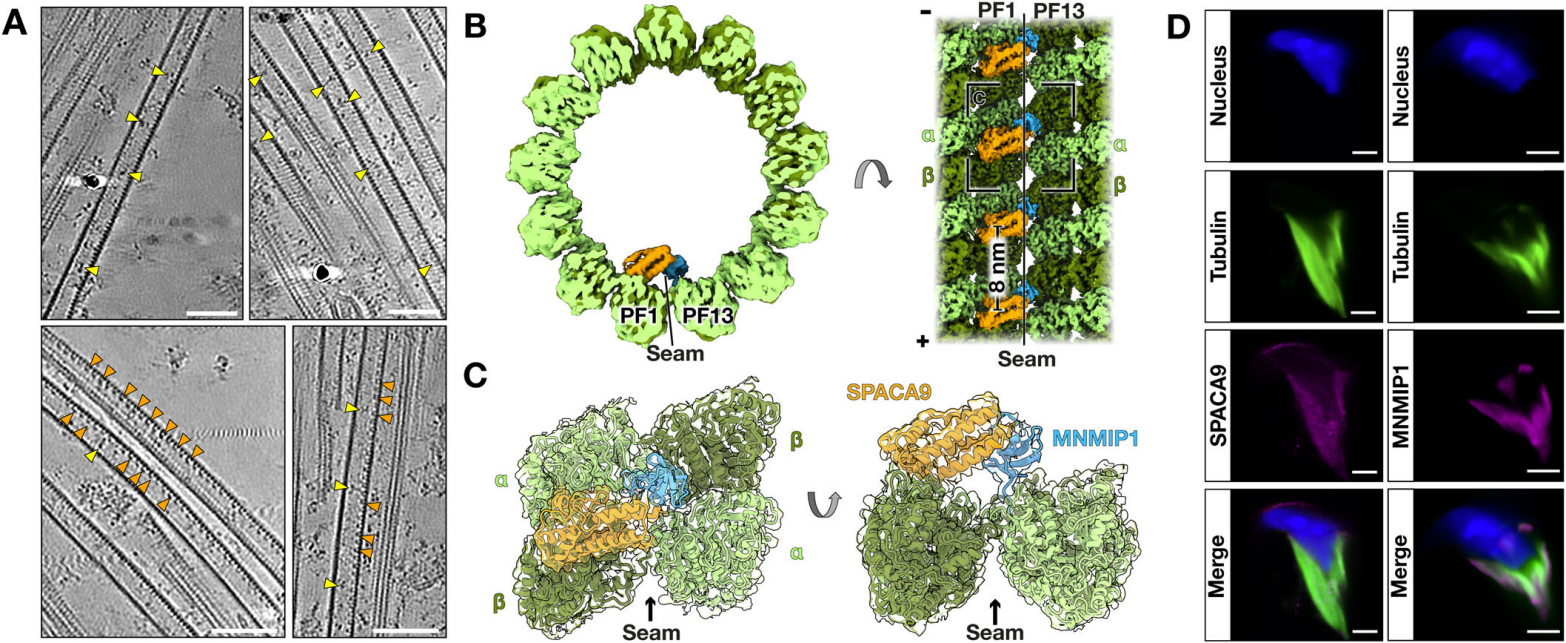
SPACA9 and MNMIP1 form a bridge at the microtubule seam. (**A**) Tomographic slices of manchette microtubules reveal non-uniformly distributed microtubule inner proteins (yellow arrowheads) and microtubule inner proteins regularly binding the lattice with a periodicity of 8 nm (orange arrowheads). Scale bars, 50 nm. (**B**) Cross-section and longitudinal section of single particle analysis 3D reconstruction of 8 nm manchette microtubule repeat, resolving the molecular bridge formed at the seam. (**C**) Molecular model of SPACA9 (orange) and MNMIP1 (blue) bound at the seam, the outline is the cryo-EM map. (**D**) STED imaging using antibodies against SPACA9 and MNMIP1 confirms their localization to the manchette in intact mouse spermatids. Scale bars, 2 μm. Source data are available online for this figure.

**Figure EV1:**
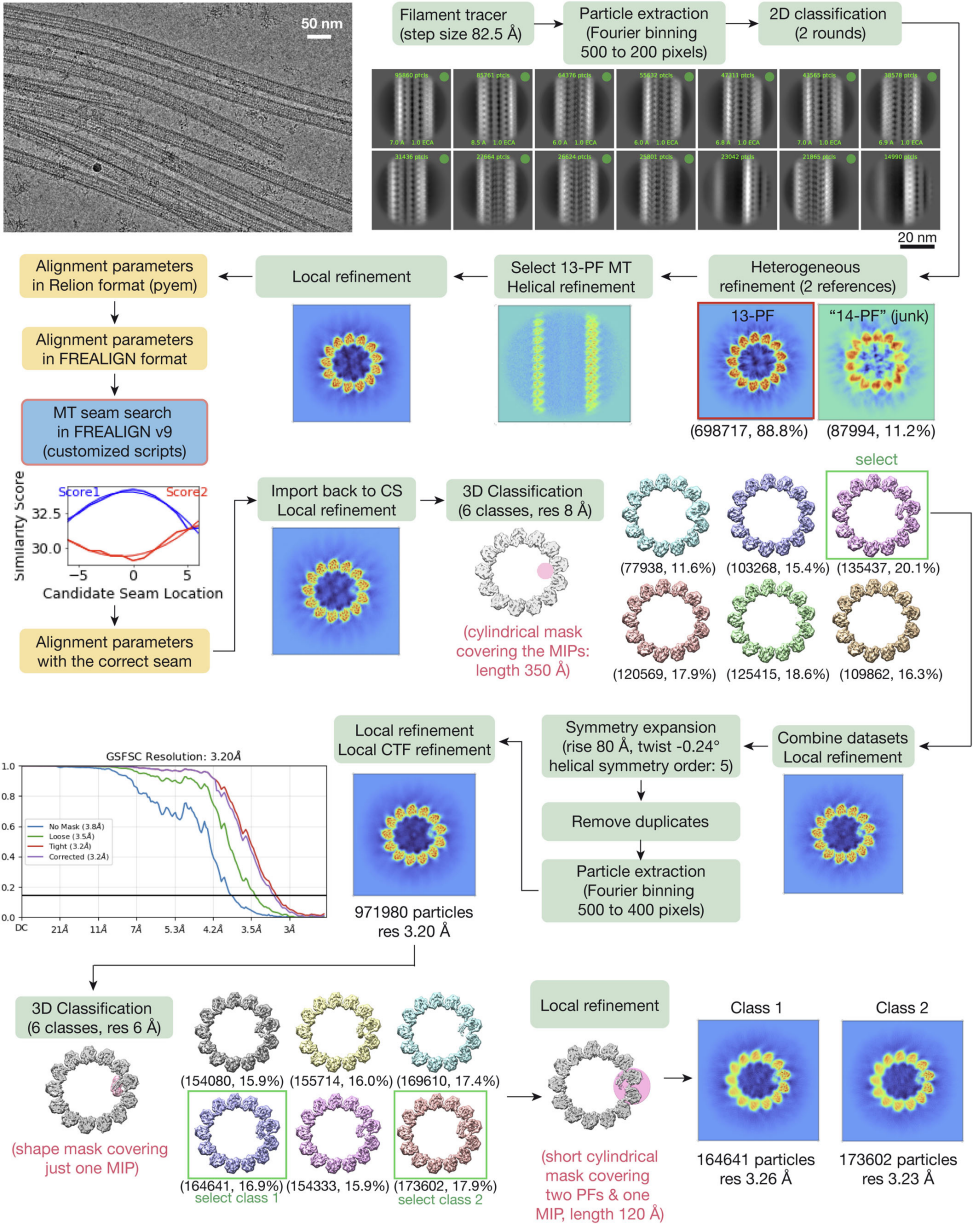
Cryo-EM single particle analysis workflow of the 8 nm manchette microtubule repeat.

**Figure EV2:**
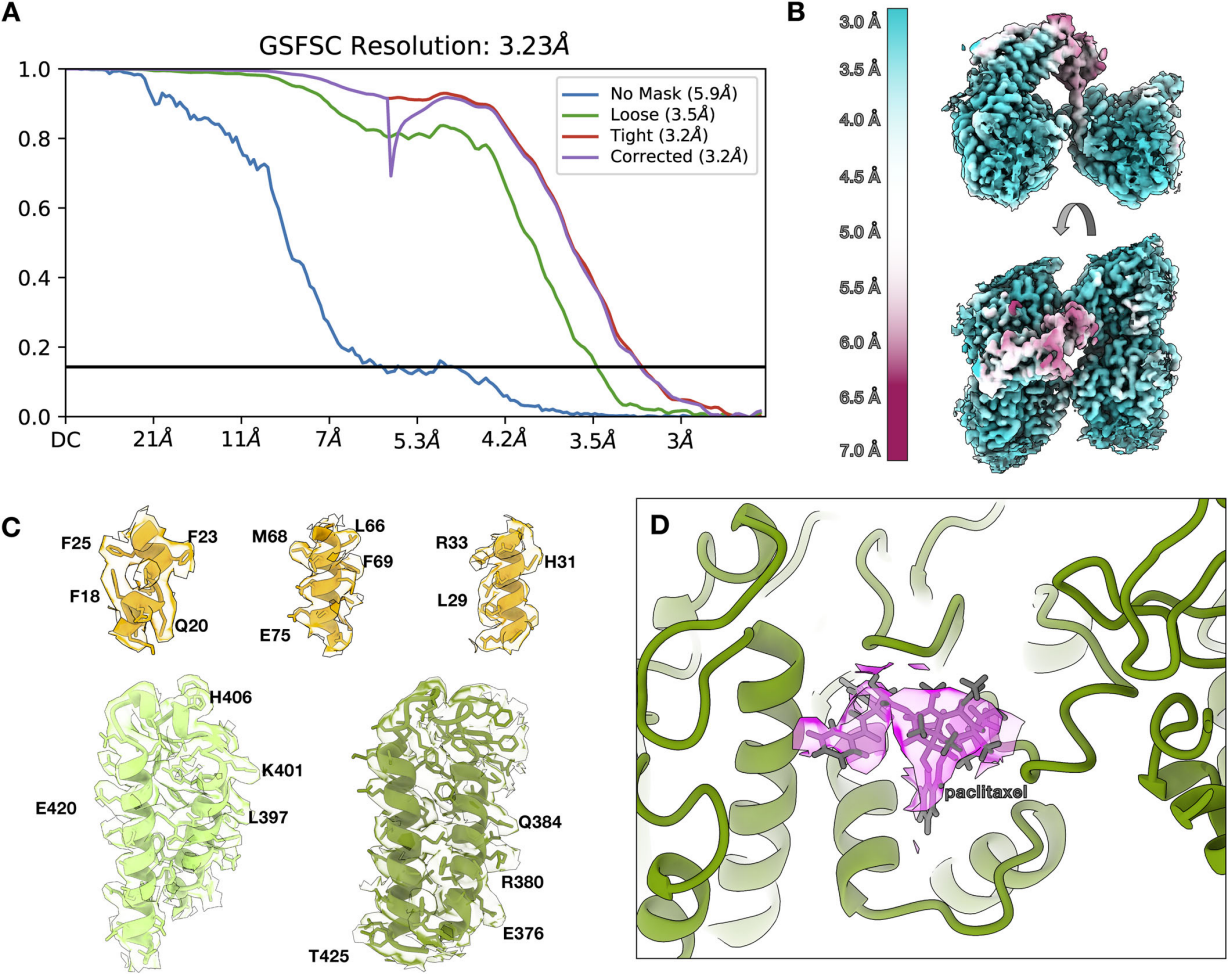
Resolution estimation of the cryo-EM SPA map. (**A**) FSC curve of the SPA map of the 8 nm repeat of a manchette MT. (**B**) Local resolution map of the seam region. (**C**) Fitting of the build model of SPACA9 (orange), α-tubulin (light green), and β-tubulin (dark green) into the cryo-EM density map. (**D**) Cryo-EM density for paclitaxel bound to manchette MTs.

**Figure EV3:**
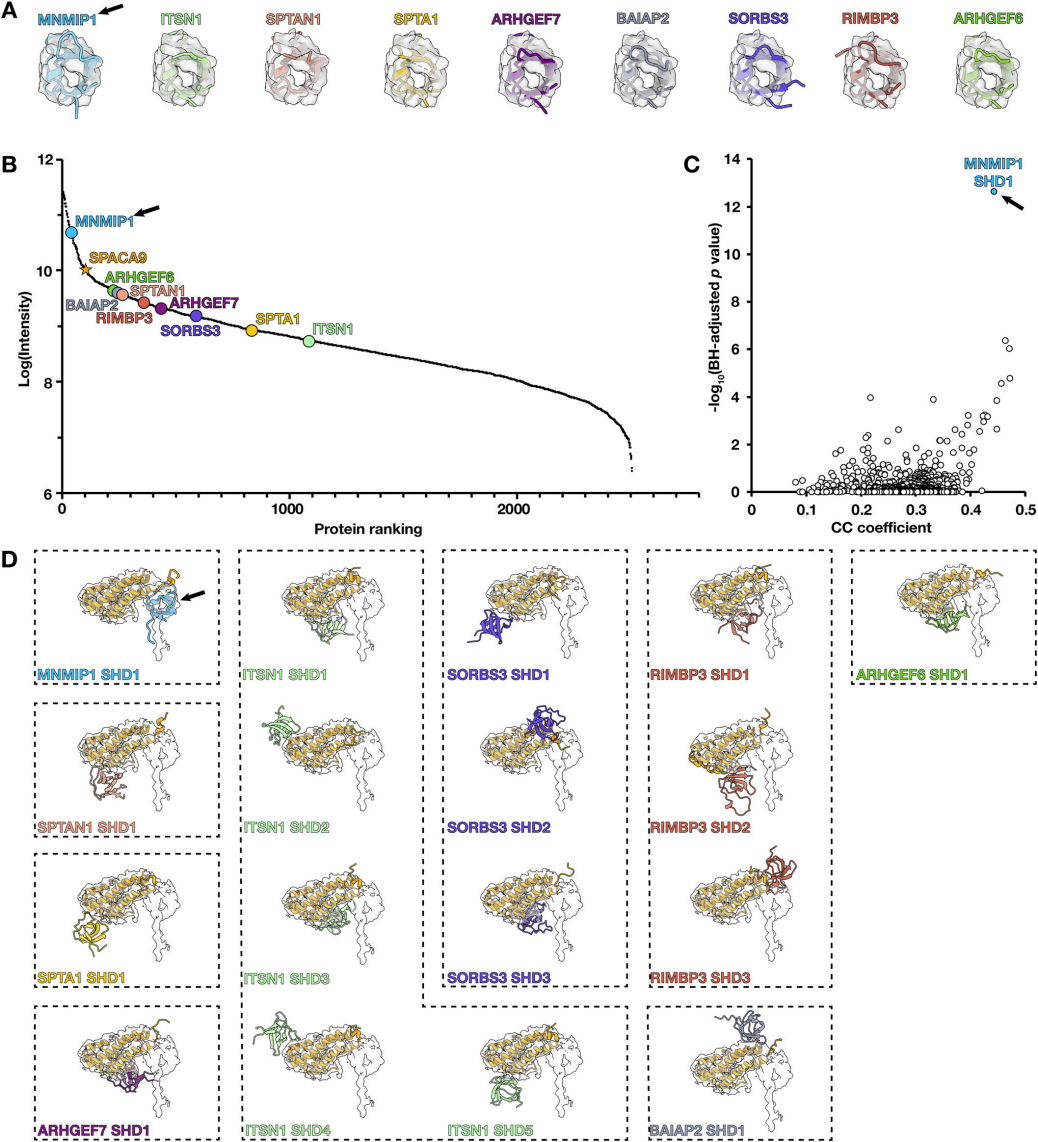
Identification of MNMIP1. (**A**) Fit of all SH3 domain proteins in the manchette proteome into the cryo-EM map. (**B**) Relative abundance of SH3 domain-containing proteins in the proteomics of isolated rat manchettes. (**C**) Scatterplot of the DomainSeeker results showing the negative log value of the BH-adjusted p value versus the cross-correlation coefficient of candidates from proteomics data of purified manchettes (Judernatz et al, 2025) into the EM density. The point of MNMIP1 SH domain 1(SHD1) is well separated from the rest of the points cloud, indicating a well-matched fit into the density. (**D**) AlphaFold3 predictions of all SH3 domains in complex with SPACA9 show the SH3 domain of MNMIP1 in the correct position.

**Figure EV4:**
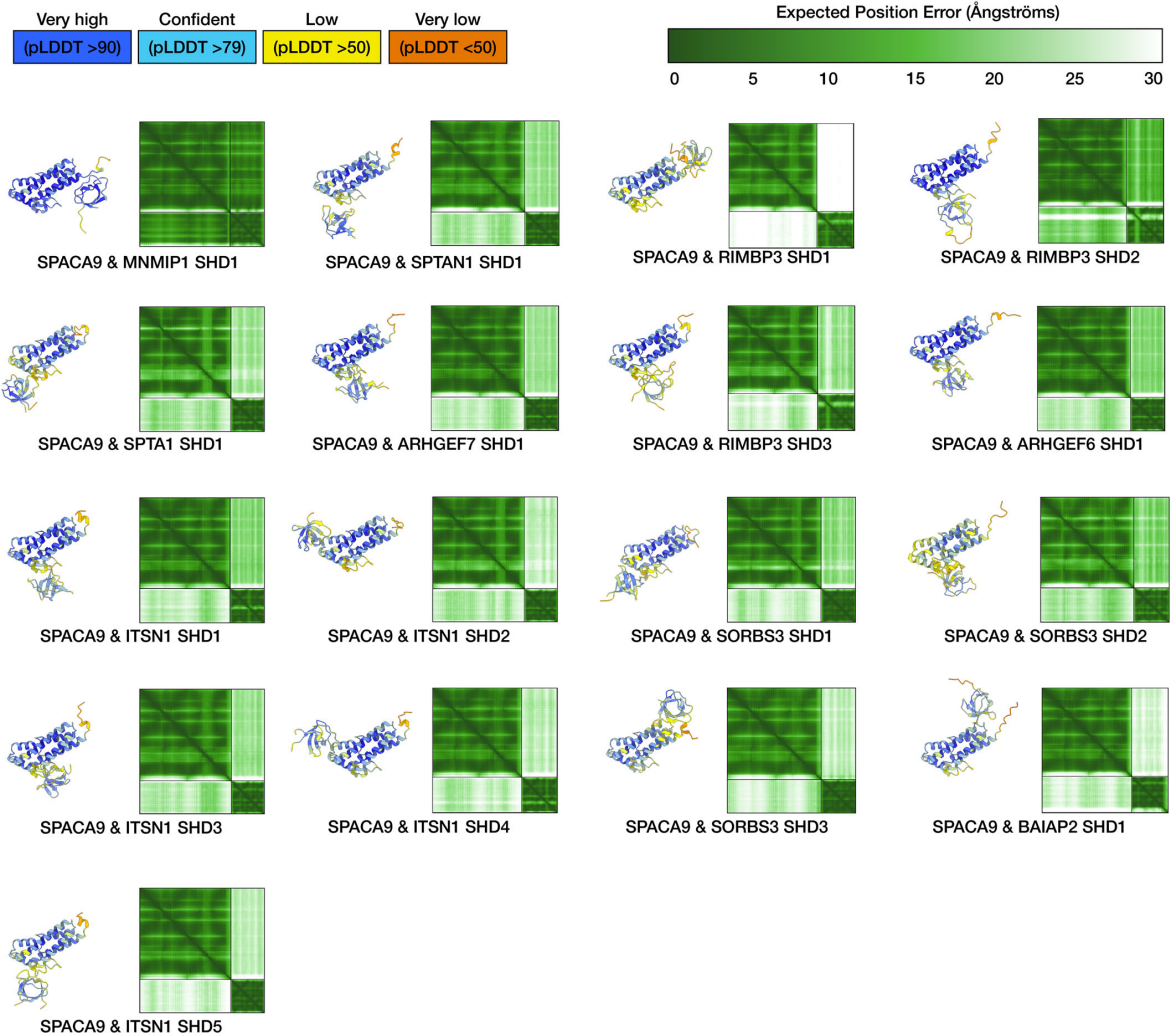
plDDT scores and PAE plots for AlphaFold3 predictions of SPACA9 and SH3 domains containing proteins in the proteomics data set of purified rat manchettes (Judernatz et al, 2025).

Cryogenic electron tomography (Cryo-ET) of the manchette MTs revealed densities of various shapes and sizes, non-uniformly distributed across the lumen of the MTs (Fig. 3A, yellow arrowheads). In addition, we found a density regularly binding the lattice with a periodicity of 8 nm (Fig. 3A, orange arrowheads). To resolve the regularly spaced densities, we used cryo-EM single particle analysis (for details see “Methods” and Figs. EV1 and EV2). The resulting map at 3.2 Å overall resolution revealed a protein density bridging the MT seam diagonally (between protofilaments 1 and 13) every 8 nm (Fig. 3B). Using the AI-based software ModelAngelo (Jamali et al, 2024), which is based on side-chain probabilities, we identified SPACA9 (local resolution 3–5 Å) as corresponding to part of the density bound along protofilament 1 (PF1) (Fig. 3C and EV2). To identify the protein density binding between PF1 and PF13 (local resolution 4–6 Å), we used a domain docking-based approach. We first compiled a library of individual domains (Gao et al, 2024) of AlphaFold-predicted structures (Abramson et al, 2024) of the top 1000 proteins in the isolated rat manchette proteome (Judernatz et al, 2025). Each domain was then docked into the cryo-EM density map, followed by scoring (for details see “Methods”) (Gao et al, 2024). This approach identified the second density as a Src homology 3 (SH3) domain (Fig. EV3C). In the manchette proteome (Judernatz et al, 2025), there are nine SH3 domain-containing proteins, with SH3 domain-containing protein 21 (SH3D21) being the most abundant (Fig. EV3B), and the SH3 domain of SH3D21 gave the best fit parameters (Fig. EV3C). We then used AlphaFold3 to predict a complex between SPACA9 and all SH3 domains identified in the manchette proteome. Only the predicted SPACA9- SH3D21 complex agreed with our experimental cryo-EM density (Fig. EV3D). The AF3 prediction of SPACA9- SH3D21 also generated the highest predicted local distance difference test (pLDDT) confidence and predicted aligned error (PAE) plots (Fig. EV4). We further used IF and subsequent stimulated emission depletion (STED) microscopy on fractions enriched for elongating mouse spermatids to confirm localization of SPACA9 and SH3D21 to the manchette (Fig. 3D; Movie EV4). Following this newly established role of SH3D21, its name has been changed to manchette MT inner protein 1 (MNMIP1).

**Figure 4:**
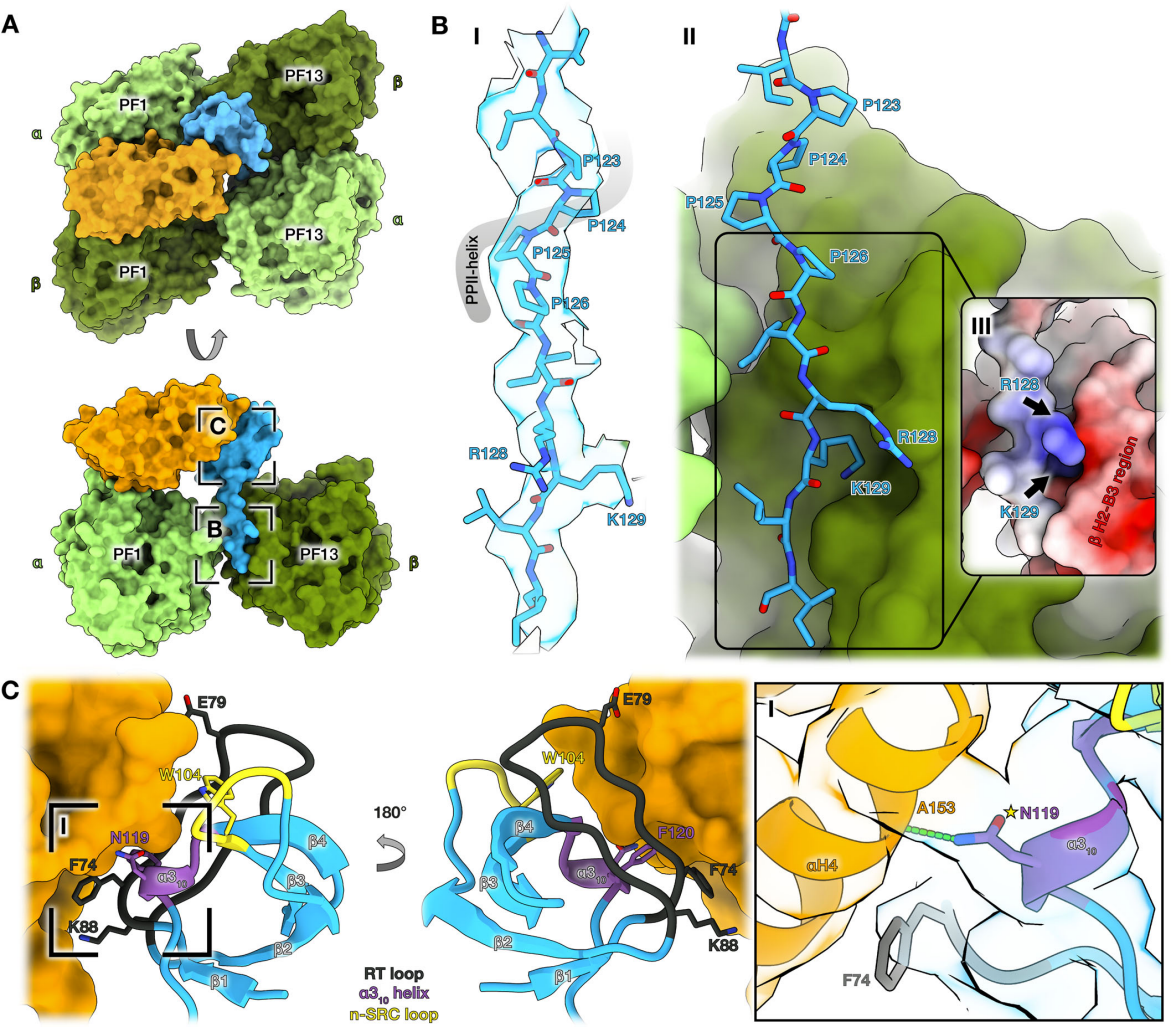
MNMIP1 threads the microtubule lattice at the seam. (**A**) Orthogonal views showing the mode of binding of SPACA9 (orange) and MNMIP1 (blue) to tubulin heterodimers (green). (**B**) MNMIP1 binds to PF1 with residues 122–131 extending between PF1 and PF13. The extension is structurally supported by a conserved stretch of prolines (residues 123–126) (I and II), and an interaction of the negatively charged residues in the H2-B3 region of β-tubulin with conserved positively charged residues in positions 128 and 129 of MNMIP1 (III). (**C**) The interaction interface between MNMIP1 and SPACA9 (view rotated −30° compared to (**A**)). The interaction is mediated by the SH3 domain RT loop (black), α3_10_ helix (violet), and n-SRC loop (yellow). I, a zoom-in on the highly conserved residue N119 in α3_10_ helix, which forms a hydrogen bond with alpha chain backbone of helix αH4 in SPACA9. Source data are available online for this figure.

**Figure EV5:**
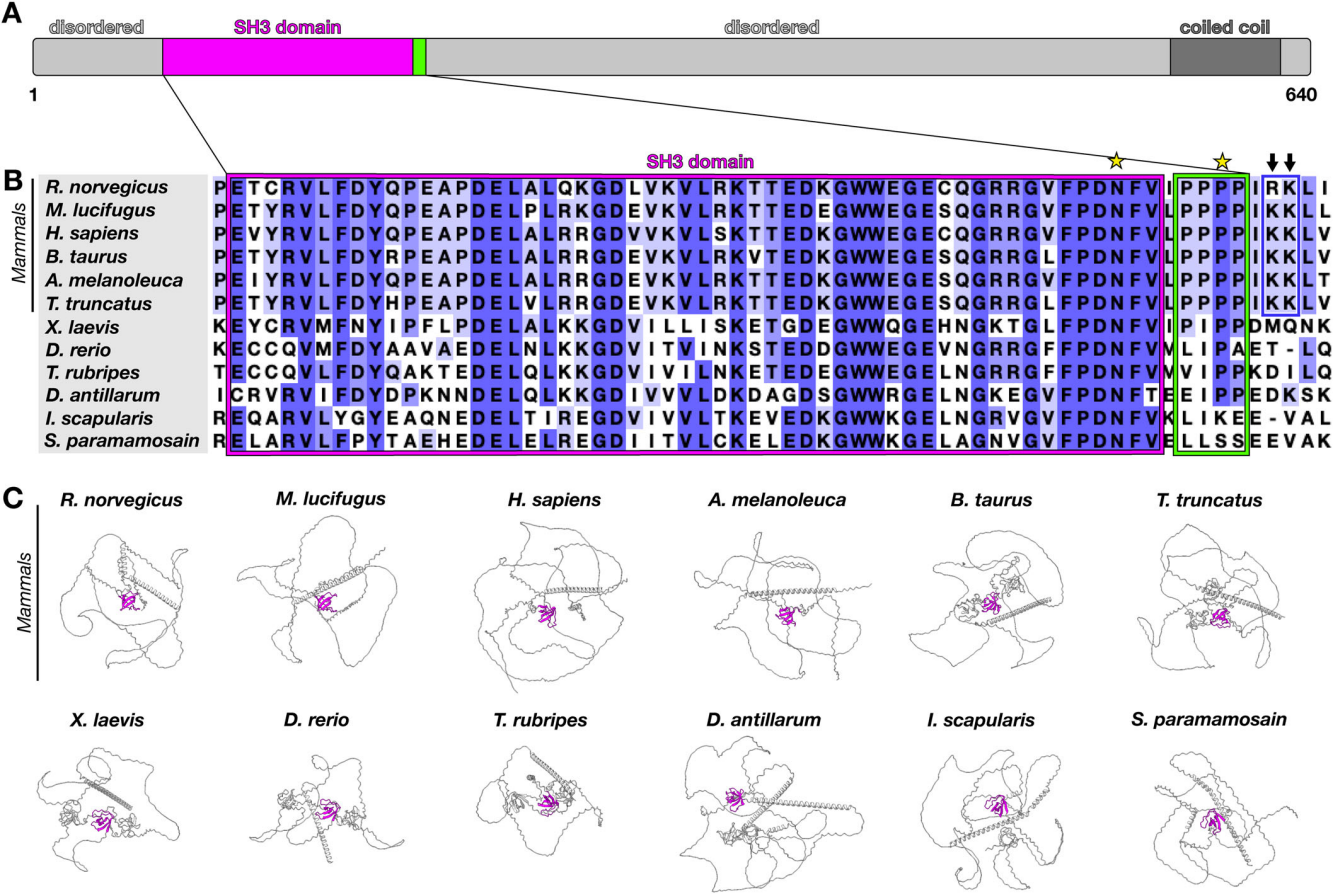
MNMIP1 SH3 domain is highly conserved. (**A**) Scheme of domain organization of MNMIP1. (**B**) Sequence alignment of MNMIP1 from several species reveals high conservation of the SH3 domain. Blue color indicates the grade of amino acid conservation. The SH3 domain is marked in a magenta box. The region of the polyproline-II helix is marked in a green box. (**C**) AlphaFold3 models of full-length MNMIP1 from the same species in (**B**) reveal the presence of up to three SH3 domains. Magenta marks the SH3 domain identified via sequence alignment, as shown in (**B**).

To analyze how SPACA9 and MNMIP1 bridge the MT seam, we modeled them into the density map (Figs. 3C and 4A,B) (see “Methods” for details). The model revealed that SPACA9 binds to both α- and β-tubulin of PF1 but does not interact directly with PF13 (Fig. 4A), while MNMIP1 binds to the β-tubulin in PF13 with residues 122–131 threaded between PF1 and PF13 (Fig. 4A,B). The region of amino acids 122–131 of MNMIP1 is characterized by a conserved repeat of four prolines forming a left-handed polyproline (PP) II helix (Figs. 4B and EV5). The PPII helix orients the following amino acids perpendicular to the MT lattice, which allows two conserved positively charged amino acids (R128 and K129 of MNMIP1) to interact with the negatively charged residues in the H2-B3 region of β-tubulin (Fig. 4B). The SH3 domain of MNMIP1 is on the luminal side of the MT and is composed of four beta sheets (β1–4) that form a small beta barrel domain, a short helix (α3_10_), and two loops (RT loop and n-SRC loop). Here, MNMIP1 interaction with SPACA9 is mediated through the RT and n-SRC loops as well as the α3_10_ helix. The beta sheets face away from SPACA9 (Fig. 4C). A closer look at the interaction site revealed a density suggesting a hydrogen bond between N119 (α3_10_ in MNMIP1) and the alpha carbon of A153 in helix αH4 (SPACA9) (Fig. 4C). Sequence alignment of MNMIP1 indicated that N119 is conserved (Fig. EV5A,B), suggesting it may be required for the interaction with SPACA9. The remaining portion of MNMIP1 is unresolved in our structure, with the N-terminus likely residing in the lumen of the MT and the C-terminus on the outside of the MT.

**Figure EV6:**
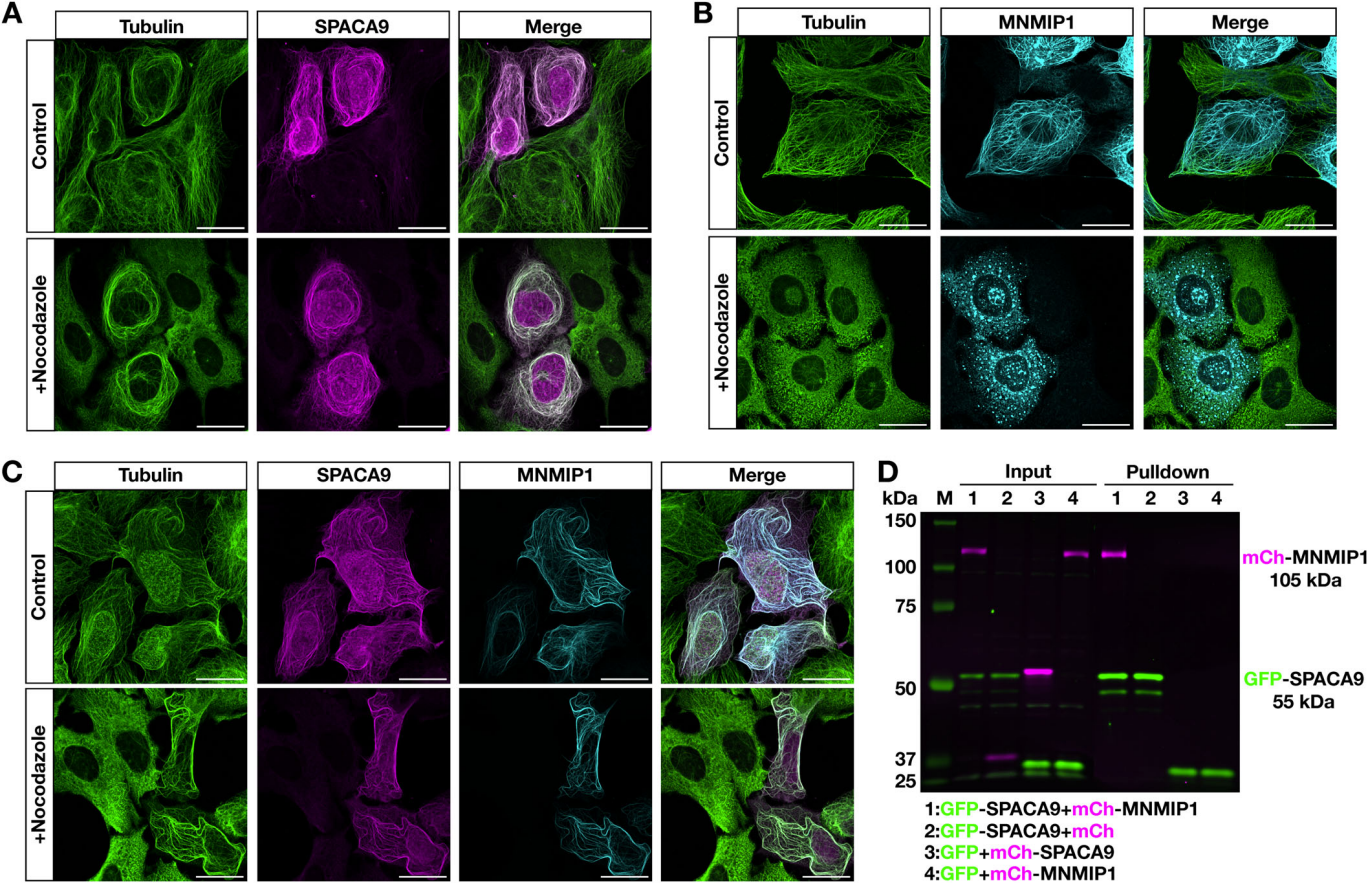
SPACA9 and MNMIP1 interact in vitro and stabilize MT. (**A**) Overexpressed mCherry-SPACA9 in U2OS cells colocalizes with microtubules and increases microtubule stability against treatment with 10 μM nocodazole for 15 min. Scale bars, 25 µM. (**B**) Overexpressed EGFP-MNMIP1 in U2OS cells colocalizes with microtubules but does not increase microtubule stability against treatment with 10 μM nocodazole for 15 min. Scale bars, 25 µM. (**C**) Co-expression of mCherry-SPACA9 and EGFP-MNMIP1 in U2OS cells shows colocalization of both proteins on microtubules and increases microtubule stability against treatment with 10 μM nocodazole for 15 min. Scale bars, 25 µM. (**D**) Co-immunoprecipitation of MNMIP1 and SPACA9 confirms the interaction between the proteins in heterologous cells.

To confirm that SPACA9 and MNMIP1 can bind to MTs also in-vitro, we tagged them with GFP and mCherry and overexpressed them in transiently transfected U2OS cells. We found that they both colocalize with MTs, either individually or when co-expressed (Fig. EV6). Interestingly, SPACA9 induced MT bundle formation when overexpressed (Fig. EV6A). Furthermore, co-expression of both SPACA9 and MNMIP1 resulted in colocalization on MT bundles (Fig. EV6C). To test whether the proteins increase MT stability, cells were treated with the MT depolymerizing compound nocodazole. Upon nocodazole treatment, MTs bound by either SPACA9 alone or by SPACA9 and MNMIP1 remained stable while undecorated MTs depolymerized (Fig. EV6A–C). This suggests that SPACA9, either alone or together with MNMIP1, increases MT stability, whereas MNMIP1 by itself does not stabilize MTs. To demonstrate direct binding between SPACA9 and MNMIP1, we performed pull-down assays. Using an antibody against GFP, we could pull mCherry-MNMIP1 from cells where it was co-expressed with GFP-tagged SPACA9 but not GFP alone (Fig. EV6D).

**Figure 5:**
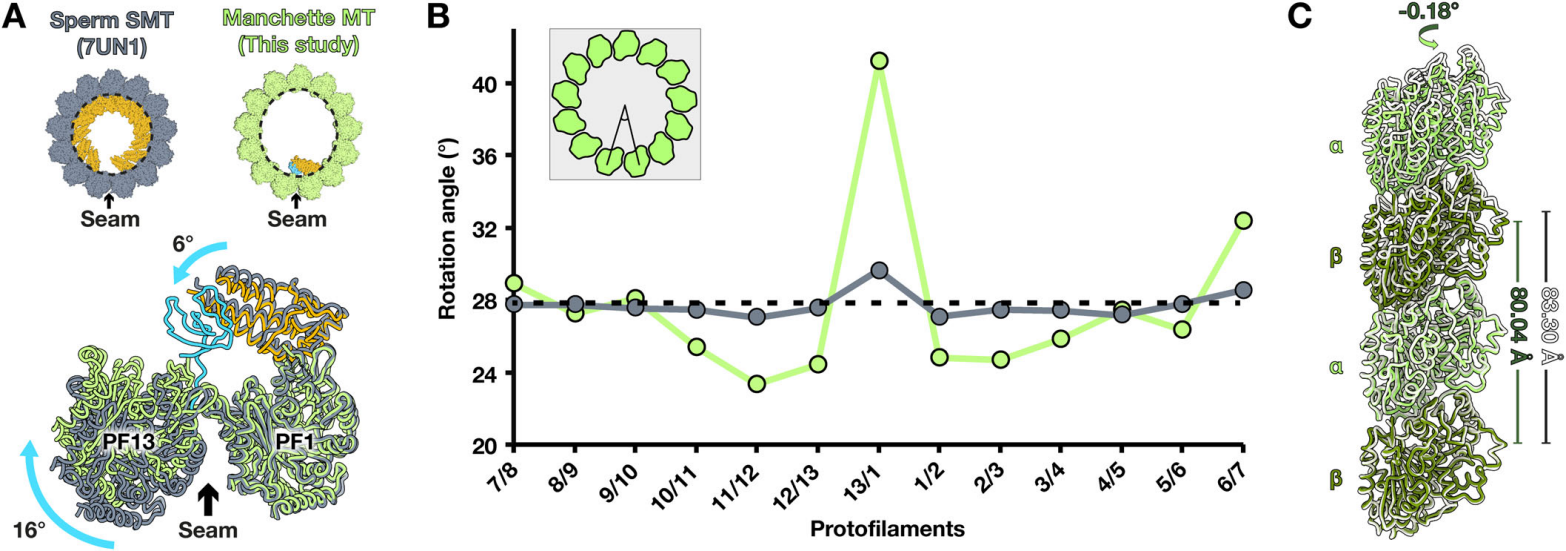
Manchette microtubules are compacted and slightly elliptical. (**A**) Comparison between SPACA9-bound microtubule from sperm endpiece (pdb:7UN1; gray) and manchette microtubule (green) with SPACA9 (orange) and MNMIP1 (blue) shows that the binding of SPACA9 and MNMIP1 at the seam brings protofilament 1 (PF1) and 13 (PF13) closer. (**B**) Plot of rotation angles between adjacent protofilaments for manchette microtubules (green) and sperm singlet microtubules (pdb:7UN1; gray). The dashed line indicates the angle of a canonical 13 PF microtubule of 27.69°. The rotation angle between PF1 and PF13 (seam) in the manchette microtubule is >40º. (**C**) Comparison of the microtubule lattice spacing between manchette microtubules (green) and in vitro polymerized 13 PF microtubules (pdb:6WVR, white). Source data are available online for this figure.

SPACA9 has been previously identified as a MIP of axonemal MTs, where it forms spiral assemblies that exclude the seam, and involves stabilizer of axonemal MTs (SAXO) proteins (Gui et al, 2022a). The binding interface of SPACA9 in axonemal MTs is similar to the one we resolved on PF1, which raises the question of why it does not bind to other protofilaments in the manchette MTs. Comparison between the MT ellipticity of manchette MTs and axonemal MTs with SPACA9 revealed that manchette MTs are more elliptical (Fig. 5A). Specifically, PF13 is rotated by ~16° relative to axonemal MTs, and SPACA9 is rotated by ~6°. Measurements of rotation angles between all neighboring PFs for manchette MTs showed that in 12 out of 13 positions, rotation angles remained between 24° and 32° (Fig. 5B), agreeing with the canonical 27.69° angle of a 13 PF MT(Lacey et al, 2019). However, the rotation angle between PF1 and PF13 was >40° (Fig. 5B). We further found that manchette MTs lattice is compacted with a lattice spacing of 80.04 Å (Fig. 5C). This is surprising, as reported paclitaxel-stabilized MT lattice spacing is typically >82 Å (Alushin et al, 2014; de Jager et al, 2025; Zhang et al, 2018) while the binding mode of paclitaxel is similar to the previously reported one (Fig. EV2D) (Prota et al, 2023). Furthermore, manchette MTs show a negative protofilament twist of −0.18° (Fig. 5C). Taken together, the binding of SPACA9 and MNMIP1 seems to bring PF1 and PF13 closer, causing the observed local rotation and increased ellipticity for manchette MTs. This elliptic MT is likely incompatible with the binding of additional SPACA9 and prevents the formation of spiral assemblies like those in axonemal MTs.

## Discussion

The manchette is a unique MT-based structure of critical importance for shaping the sperm head (Dunleavy et al, 2019a; Rattner and Brinkley, 1972; Russell et al, 1991; O’Donnell and O’Bryan, 2014). Throughout spermiogenesis, the manchette and its MTs persist for several days (Mochida et al, 1998). This transient stability places it between highly dynamic cytosolic MTs and more stable axonemal MTs. The data presented here suggest two potential molecular mechanisms for manchette MT regulation, one at the ends and one along the lattice.

At both manchette ends, we found tapered MTs ends with extending protofilaments. Such end structures have been suggested to result from MTs rescued from catastrophe by CLASPs (Aher et al, 2018; Lawrence and Zanic, 2019), which can bind to both MT plus and minus ends in vitro (Lawrence et al, 2018). In agreement, we showed that a CLASP2 antibody localizes to the entire manchette. Many MT end-binders can bind along the MT lattice, an effect more pronounced at higher protein concentrations, and this is well-established for CLASPs (Foster et al, 2022; Aher et al, 2018; Lawrence and Zanic, 2019). As CLASP2 binds to both plus and minus ends, and can associate with the MT lattice (albeit more weakly), the signal is across the entire manchette. Our results suggest CLASP2 as a crucial component of the manchette. The protein likely stabilizes manchette MTs at their plus and minus ends and regulates MT dynamics across the entire manchette. Characterization of manchettes in CLASP2 knock-out mice will help to further understand CLASP2 function in the manchette and during spermiogenesis.

Along the manchette lattice, we revealed that the MT seam is bridged by one copy of SPACA9 and one copy of MNMIP1, every 8 nm. To the best of our knowledge, this is the first observation of a seam binding MIP outside axonemal MTs. Both SPACA9 and MNMIP1 are highly enriched during spermiogenesis and have been studied in the context of sperm formation. Yet, a single knockout of either SPACA9 or MNMIP1 does not seem to affect fertility in mice (Castaneda et al, 2021; Nguyen et al, 2024). However, the effect on the ultrastructure of manchette and its MTs was not investigated in these KO studies and should be revisited in the future, together with double KO of both proteins. We demonstrated that MNMIP1 threads the MT lattice via a PPII helix (Fig. 4B). A substantial portion of the protein thus might be on the outside of the MT, but we were not able to resolve that part, and AlphaFold3 predictions of full-length MNMIP1 reveal highly disordered regions following the SH3 domain (Fig. EV5). Future studies on the interaction of the external region of MNMIP1 with other proteins or the disordered tails of MT with cross-linking mass spectrometry can clarify its role. Furthermore, while in many mammals MNMIP1 is predicted to contain only one SH3 domain, in ungulates and most other metazoans the protein contains two to three SH3 domains instead (Fig. EV5C). Interestingly, humans have multiple isoforms, with one predicted to have three SH3 domains. Hence, it will be valuable to investigate whether the seam bridge has a different organization in the manchette MTs of non-mammalian organisms and investigate the potential role of spermatid-specific splicing isoforms in humans.

It has been shown that hydrolysis of GTP to GDP considerably destabilizes the seam (LaFrance et al, 2022; Zhang et al, 2018), making it a weak point relevant for MT disassembly (Zhang et al, 2015; Zhang and Nogales, 2015; LaFrance et al, 2022; Zhang et al, 2018; Debs et al, 2020). With the continuous binding periodicity, it is plausible that SPACA9 and MNMIP1 function as a ‘molecular staple’. Thus, the function of the seam bridge formed by SPACA9 and MNMIP1 could stop the seam opening and prevent the initiation of MT depolymerization. In line with this, expression of tagged proteins in U2OS cells increased MT stability against treatment with the MT depolymerizing agent nocodazole. A similar role for MT seam stabilization was suggested for SPEF1, a MT-associated protein that binds the seam in between the axonemal central pair (Han et al, 2022; Gui et al, 2022b; Legal et al, 2025). Related, the high local rotation observed might be a result of rescuing a destabilized seam from opening, as rotational deviations have been reported to be strongest at the seam and might underlie seam opening (Debs et al, 2020). Intriguingly, we observed that the manchette MT lattice is compacted. Specificity to particular MT lattice spacing was suggested to play a role in regulating MT-associated protein binding (Ettinger et al, 2016; Zhang et al, 2017; de Jager et al, 2025; Prakash et al, 2025). Further studies are required to determine the extent to which SPACA9 and MNMIP1 require the observed compacted lattice and negative protofilament twist to bind the seam.

In summary, during spermiogenesis, the manchette MTs require active regulation to maintain the manchette’s function. Our data indicate two distinct but complementary mechanisms for manchette MT regulation: Stabilization of MT ends by CLASP2 and bridging of the MT seam by a SPACA9-MNMIP1 complex to prevent seam opening and maintain lattice integrity. While SPACA9 is a well-established MT-binding protein in axonemal MTs, we present MNMIP1 as a previously unidentified MT-binding protein that, when in complex with SPACA9, is able to bind to the seam of manchette MTs, marking the first observation of a seam-binding protein complex beyond axonemal MTs.

## Methods

**Table:** Reagents and tools table

Reagent/resource	Reference or source	Identifier or catalog number
**Experimental models**		
Wistar (*Rattus norvegicus*)	Instantie voor Dierenwelzijn (IvD) Utrecht	RGD_13508588
Crl:CD(SD) (*Rattus norvegicus*)	Instantie voor Dierenwelzijn (IvD) Utrecht	RGD_737903
RJHan:WI (*Rattus norvegicus*)	Instantie voor Dierenwelzijn (IvD) Utrecht	RGD_38676310
Crl:LIS (*Rattus norvegicus*)	Instantie voor Dierenwelzijn (IvD) Utrecht	RGD_2312466
C57BL/6J	PMID 38773322	
C57Bl6	PMID 27539564	
HEK293T cells	ATCC	CRL-3216
U2OS cells	ATCC	HTB-96
**Recombinant DNA**		
Human SH3D21/MNMIP1	GenScript	A4FU49-1
Human SPACA9	GenScript	Q96E40-1
**Antibodies**		
Mouse anti-α-tubulin monoclonal (B-5-1-2)	Invitrogen	Cat# 32-2500
HRP-conjugated Goat anti-Mouse IgG (H + L)	Invitrogen	Cat# 31430
Mouse anti-GFP	Roche	Cat# 11814460001
Chicken anti-GFP	Aves labs	Cat# GFP-1020
Chicken anti-mCherry	Abcam	Cat# ab205402
goat anti-mouse–and chicken IgG Alexa Fluor 488	Invitrogen	Cat# A-21200
goat anti-mouse–and chicken IgG Alexa Fluor 594	Invitrogen	Cat# A-21468
goat anti-mouse–and chicken IgG Alexa Fluor 647	Invitrogen	Cat# A-21449
Rabbit anti-RFP	Rockland	Cat# 600-401-379
donkey anti-mouse IRDye 680CW	LI-COR	Cat# NC0250903
donkey anti-rabbit IRDye 800CW	LI-COR	Cat# NC0964679
Rabbit anti-CLIP1	Invitrogen	Cat# PA5-30946
Rabbit anti-C1orf113	Proteintech	Cat# 25767-1-AP
Rabbit anti-SPACA9	Proteintech	Cat# 26034-1-AP
Rabbit anti-CAMSAP1	Proteintech	Cat# 83864-1-RR
Rat anti-CLASP2	Abcam	Cat# ab95373
Donkey anti-rabbit Alexa Fluor 594	Invitrogen	Cat# A-21207
Goat anti-mouse Atto 647 N	Sigma-Aldrich	Cat# 50185-1ML-F
Goat anti-rat Alexa Fluor 594	Invitrogen	Cat# A-11007
**Oligonucleotides and other sequence-based reagents**		
PCR primer (FW1_EGFP/mCh-SH3D21_EcoR1OH)	This study	5’- TATTCGAATTCTGGTGGAATGGAAGTGCTTGTGCTCG -3’
PCR primer (REV1_EGFP/mCh-SH3D21_BamH1OH)	This study	5’- TATATGGATCCTTAATATGTTTGGGTTTGAGTATGAATTAC 3’
**Chemicals, enzymes, and other reagents**		
Paclitaxel	Invitrogen	Cat# P3456
Fugene6	Promega	Cat# E2693
Nocodazole	Sigma-Aldrich	Cat# M1404
Formaldehyde 4%	Invitrogen	Cat# FB002
HEPES	Merck	Cat# H4034-500G
KCl	Merck	Cat# 60128-1KG-F
NaCl	Merck	Cat# 71376-1KG
Tris-HCl	Merck	Cat# 93363-500 G
Glycine	Merck	Cat# G8790-1KG
MgSO4	Merck	Cat# M2773-500G
EGTA	Merck	Cat# E3885-25G
EDTA	Merck	Cat# 03677-100 G
DTT	Merck	Cat# 11583786001
GTP	Thermo Fisher Scientific	Cat# R0461
Triton X-100	Merck	Cat# X100
IGEPAL CA-630	Sigma-Aldrich	Cat# I8896
SDS	Merck	Cat# 71725-100 G
Penicillin-streptomycin	Invitrogen	Cat# 15140-122
Polyethylenimine	Polysciences	Cat# 23966-100
cOmplete protease inhibitor	Roche	Cat# 11697498001
Sucrose	Merck	Cat# S0389-1KG
PBS	Corning	Cat# 21-031-CV
Collagenase	Sigma-Aldrich	Cat# 9001-12-1
DNaseI	Sigma-Aldrich	Cat# 9003-98-9
DMEM	Invitrogen	Cat# 61965-059
DMSO	Merck	Cat# D2650-100ML
BSA	Merck	Cat# A7030-100G
Tween-20	Merck	Cat# P1379-1L
Poly-L-lysine	Sigma-Aldrich	Cat# P4832
DAPI	Thermo Fisher Scientific	Cat# 62248
Mounting medium Prolong Diamond	Invitrogen	Cat# P36965
Methanol	Baker	Cat# 61008045.2500
Ethanol	Avantor	Cat# 83813.360
ChromoTek GFP-Trap magnetic beads M-270	Proteintech	Cat# AB_2827592
AURION Gold Tracers BSA conjugated 10 nM	AURION	Cat# 110.122
**Software**		
cryoSPARC v4	https://cryosparc.com	
RELION v5	https://relion.readthedocs.io/en/release-5.0/#	
FREALIGN	https://grigoriefflab.umassmed.edu/frealign	
MotionCor2 1.2.1	https://emcore.ucsf.edu/ucsf-software	
IMOD4.9	https://bio3d.colorado.edu/imod/	
AreTomo v2	https://github.com/czimaginginstitute/AreTomo2	
Cylindra	https://hanjinliu.github.io/cylindra/	
CryoCare v0.3	https://github.com/juglab/cryoCARE_pip/releases	
ModelAngelo	https://github.com/3dem/model-angelo	
DomainFit	https://github.com/builab/DomainFit	
ChimeraX	https://www.cgl.ucsf.edu/chimerax/	
Coot	https://www2.mrc-lmb.cam.ac.uk/personal/pemsley/coot/	
PHENIX	https://phenix-online.org/download	
iMSPECTOR	https://abberior.rocks/superresolution-confocal-systems/imspector/	
**Other**		
Titan Krios 3 CryoTEM	Thermo Fisher Scientific	
Talos Arctica CryoTEM	Thermo Fisher Scientific	
Beckman Coulter Optima XPN-80 ultracentrifuge	Beckman Coulter	
Gel Doc XR system	Bio-Rad	
ECL western blot detection reagent	Cytiva Amersham	
IX83	Olympus	
Infinity Line easy3D STED	Abberior	
Axio Imager Z2 LSM900	Zeiss	
Odyssey CLx Infrared Imaging System	LI-COR	

### Manchette isolation

Isolation of manchettes from rat testes was based on a previously published protocol (Mochida et al, 1998). Briefly, testes were dissected from freshly asphyxiated rats of reproductive age (Wistar, Crl:CD(SD), RJHan:WI, Lister Hooded). Gonadal fat tissue and the epididymides were carefully removed using sharp scissors. The testes were then transferred to a culture dish containing PBS on ice, and the tunica albuginea was removed using tweezers. The seminiferous tubules were briefly washed and then transferred into 10 ml of MT-stabilizing buffer (25 mM HEPES, pH 7.4, 2.5 mM MgSO_4_, 2.5 mM EGTA, 15 mM KCl, 5 mM DTT, 0.1 mM GTP, 20 μM paclitaxel, 1% Triton X-100, 1 Tablet of cOmplete mini-Protease Inhibitor). Next, the seminiferous tubules were chopped using a razor blade and then repeatedly pipetted with a 1-mL pipette to homogenize the tissue and separate the manchettes. The suspension was filtered through 100 µm, 30 µm, and 10 µm mesh-sized cell strainers and collected in a 50 mL Falcon tube. The suspension was further filtered through 7.5 g of 212–300-μm glass beads packed in a 20 mL syringe and prewashed with ice-cold PBS (pH 7.4). After filtration, 6 mL of the suspension was added to 44 mL cold 2.5 M sucrose. The resulting 50 mL suspension was split equally into two open-top thin-wall ultra-centrifugation tubes (Beckmann Coulter #344058). The mixture was layered with 6 mL of cold 2.05 M sucrose followed by 6 mL of cold 1 M sucrose and centrifuged at 85,000 × *g* for 110 min at 4 °C using a SW32 rotor in a Beckman Coulter Optima™ XPN-80 ultracentrifuge. Approximately 3 mL of intact manchettes were harvested from each tube at the interface between the 1 M and 2.05 M sucrose and transferred into 12 mL cold PBS. The mixture was then centrifuged at 1000 × *g* for 30 min at 4 °C, the supernatant was discarded, and the pellet was resuspended in 500 μL PBS. The manchette-containing pellets were washed three times by resuspension in cold PBS and finally used for further analysis.

### Spermatid enrichment

Testes were dissected from freshly asphyxiated rats or mice. After removing the tunica albuginea, the seminiferous tubules were dissociated and mechanically cut into small pieces with a sharp razor blade. The tissue of each testis was pelleted at 220 × *g* for 10 min, the supernatant was carefully discarded, and the pellet was resuspended in a mixture of 8 mL of collagenase (0.5 mg/mL, Sigma #9001-12-1), and DNaseI (0.5 mg/mL, Sigma #9003-98-9). Enzymatic digestion was performed at 37 °C for 20 min to remove connective tissue and interstitial cells. The suspension was then centrifuged for 10 min at 220 × *g* at room temperature. The pellet was thoroughly resuspended in 10 mL DMEM and repeatedly pipetted to dissociate the cells mechanically. The cell suspension was filtered through a 30-μm nylon sieve to remove residual cell clumps and undigested tissue. The filtered cell suspension was loaded onto a modified STAPUT gradient of 100 mL 2–4% w/v bovine serum albumin/DMEM gradient as described previously (Dunleavy et al, 2019b; Cafe et al, 2020). Germ cell stages were fractionated based on size and density via velocity sedimentation for 3.5 h at room temperature. Seven fractions were collected: 10 mL for fractions 1–3 and 5–7, and 15 mL for fraction 4. Cells from fractions 2 (spermatocytes), 5 (round spermatids), 6 (round and elongating spermatids), and 7 (elongating spermatids and spermatozoa) were harvested at 220 × *g* for 10 min at room temperature and washed once with DMEM.

For cryopreservation, a freezing medium was prepared by mixing 20% dimethyl sulfoxide (DMSO) in fetal bovine serum (FBS), sterile-filtered through a 0.22-µm membrane, and stored at −20 °C. Purified spermatid suspensions were combined 1:1 with the freezing medium (e.g., 300 µL cell suspension with 300 µL freezing medium), resulting in a final composition of 10% DMSO, 50% FBS, 39.5% DMEM, and 0.5% penicillin–streptomycin. The mixture was transferred into cryovials, placed in a Mr. Frosty isopropanol-based freezing container, and stored at −80 °C for 24 h. The vials were transferred to cryostorage boxes and stored at −80 °C. For thawing, cryovials were incubated in a 37 °C water bath for 1 min and immediately diluted into 1 mL of pre-warmed DMEM supplemented with 10% FBS and 1% penicillin–streptomycin. Cells were centrifuged at 220 × g for 10 min, the supernatant was discarded, and the pellet was resuspended in fresh medium for further processing.

### Immunoblotting

Purified manchettes in suspension were denatured in SDS sample buffer (50 mM Tris-HCl, pH 6.8, 2.5% β-mercapto-ethanol, 2% SDS, 0.02% bromophenol blue, 10% glycerol) and boiled for 5 min. Samples were loaded on precast Mini-PROTEAN TGX Gel (Bio-Rad) (5% stacking gel, 12% running gel) and run for 40 min at 180 V. The gel was blotted on a 0.2-µm PVDF membrane for 7 min using a Trans-Blot Turbo Transfer System (Bio-Rad). The membrane was blocked with 3% BSA in PBST (PBS supplemented with 0.05% Tween-20) for 1 h and then incubated with a mix of 1:1000 diluted α-tubulin monoclonal antibody (Invitrogen, #32-2500) in 1% BSA in PBST overnight at 4 °C. The following day, the blot was washed three times in PBST for 20 min and then incubated for 1 h at room temperature with 1:5000 HRP-conjugated Goat anti-Mouse IgG (H + L) secondary antibody (Invitrogen, #31430) in 1% BSA PBST. The blot was then washed with PBST for 1 h with three buffer exchanges every 20 min. The blot was developed using a peroxide-based ECL western blot detection reagent (Cytiva Amersham) and imaged on a Gel Doc XR system (Bio-Rad).

### Immunostainings for STED imaging

For immunofluorescence (IF) staining of mouse spermatids, #1.5 coverslips (CARL ROTH, 53586) were pre-coated with 200 µL of poly-L-lysine (Sigma-Aldrich, P4832) and incubated for 15 min before removing excess liquid and allowing them to dry. Mouse spermatid samples were thawed and washed, after which 100–200 µL of the cell suspension was pipetted onto the coated coverslips and incubated for 30 min to 1 h to allow cell adhesion. Excess liquid was then removed, and fixation was performed by adding 200 µL of 4% formaldehyde (Invitrogen, FB002), incubating for 15 min at room temperature (RT) under a fume hood, followed by two washes with 400 µL of DPBS. Permeabilization was carried out by incubating the coverslips in 300 µL of DPBS containing 0.5% Triton X-100 for 5 min at RT, followed by three washes with 400 µL of DPBS. Blocking was performed by incubating the coverslips in 400 µL of blocking solution (DPBS with 5% goat serum) for 1 h at RT or overnight at 4 °C. Primary antibody staining was conducted by incubating the coverslips overnight at 4 °C in 300 µL of blocking solution containing the following primary antibodies: rabbit C1orf113 (MNMIP1) polyclonal antibody (Proteintech, 25767-1-AP) at 1:50, SPACA9 rabbit polyclonal antibody (Proteintech, 26034-1-AP) at 1:50, primary conjugated Alexa 488 alpha tubulin monoclonal antibody (B-5-1-2) (Invitrogen, 32-2500) at 1:250, CAMSAP1 recombinant monoclonal antibody (Proteintech, 83864-1-RR) at 1:50, CLIP1 rabbit polyclonal antibody (Thermo Fisher Scientific, PA5-30946) at 1:50, anti-CLASP2 rat antibody [KT68] (Abcam, ab95373) at 1:50, alpha tubulin monoclonal antibody (B-5-1-2) (Invitrogen, 32-2500) at 1:200. After primary antibody incubation, the coverslips were washed three times for 5 min each with 400 µL of washing solution (DPBS + 0.05% Tween-20) on a gentle shaker. Subsequently, coverslips were incubated for 1 h at RT with gentle shaking in 200 µL of secondary antibody solution, which included anti-rabbit Alexa 594 (Invitrogen, A-21207) at 1:250, anti-mouse IgG-Atto 647 N (Sigma, 50185-1ML-F) at 1:250, anti-Rat IgG (H + L) Alexa 594 (Invitrogen, A-11007), and DAPI (Thermo Fisher Scientific, #62248) at 1:1000. After incubation, coverslips were washed three times for 5 min each with 400 µL of washing solution to ensure thorough removal of unbound antibodies. Coverslips were then carefully lifted and allowed to dry for a few minutes before mounting. A drop of mounting medium (Invitrogen, P36965) was applied to a glass slide, and the dried coverslip was placed onto it, with the sample facing the mounting medium. Mounted samples were stored at 4 °C for 24 h before imaging.

### Abberior STED microscope setup

All confocal and STED imaging were performed on a commercial Abberior Infinity Line easy3D STED system built on an Olympus IX83 inverted microscope with autofocus. Samples were imaged using a ×60 1.42 NA UPLXAPO60XO oil immersion objective. A 405 nm continuous-wave laser provided excitation for DAPI. For Alexa Fluor 488 and Alexa Fluor 594, excitation was provided by pulsed lasers at 488 nm and 561 nm, respectively, both operating at 40 MHz. For ATTO 647N, excitation was provided by a pulsed 640 nm laser operating at 40 MHz. STED imaging used a pulsed 775 nm depletion laser (2.75 W, 40 MHz). Fluorescence was detected with a spectral detection unit comprising a MATRIX detector and avalanche photodiodes, using appropriate emission windows for each dye. iMSPECTOR software (Abberior Instruments) was used to control the setup and acquire images.

### Abberior STED imaging conditions

For all fixed cell experiments, DAPI and Alexa Fluor 488 were recorded in confocal mode using 405 nm continuous wave and 488 nm pulsed excitation, whereas Alexa Fluor 594 was recorded in STED mode using 561 nm pulsed excitation together with the 775 nm depletion laser. ATTO 647 N was recorded in STED mode using 640 nm pulsed excitation together with the 775 nm depletion laser. Emission was collected between 415 and 478 nm for DAPI, 498 and 551 nm for Alexa Fluor 488, 580, and 630 nm or 588 and 698 nm for Alexa Fluor 594, and 650 and 755 nm for ATTO 647N. Time-gated detection was applied to the STED channel with a gate delay of 0.75 ns and a gate width of 8 ns. The pinhole was set to 1 Airy unit for all channels. Excitation powers were 1–5% (405 nm), 3–8% (488 nm), and 9–16% (561 nm), with 6.9–18.4% used for Alexa Fluor 594 and 1.1–3.1% for 640 nm excitation of ATTO 647N, with depletion at 7–15% of the maximum output; powers were empirically adjusted to balance signal and photobleaching and held constant within each experiment. Images were acquired as two-dimensional optical sections or three-dimensional stacks with pixel sizes of 10–50 nm in the xy plane, and 50 nm z steps for volumetric datasets, pixel dwell times of 15–80 µs, and one to five line accumulations, with higher line accumulation used for the STED channel. Image acquisition was performed with iMSPECTOR, and images were exported as TIFF files.

### Cryo-ET sample preparation and data collection

Freshly purified manchettes were mixed with BSA-conjugated 10 nm gold beads (Aurion) in a 4:1 ratio, and 3.5-4 μL of the mixture was applied to glow-discharged Quantifoil R 2/1 200-mesh holey carbon grids. The grids were manually blotted from the back for 3-4 s with Whatman 1 filter paper, then plunged into liquid ethane using a manual plunger (MPI Martinsried, Germany). Cryo-ET data of manchettes were collected on a 200 kV Talos Arctica (Thermo Fisher Scientific) at the Utrecht University (UU) Electron Microscopy Centre with a pixel size of 2.17 Å or on a 300 kV Titan Krios at the Netherlands Centre for Electron Nanoscopy (NeCEN) with a pixel size of either 6.32 Å (medium magnification) or 1.36 Å (high magnification). A total of 3 datasets were collected from a total of 6 grids from 3 separate manchette preparations, making a total of 120 tilt series. Tilt series were collected using SerialEM (Mastronarde, 2003) at a target defocus of between −2 and −4 µm. Tilt series were typically recorded using strict dose-symmetric schemes, spanning ± 51° in 3° increments, with the total dose limited to ∼100 e-/Å^2^.

### Tomogram reconstruction

Motion between individual frames was corrected using MotionCor2 1.2.1 (Zheng et al, 2017). Tomogram reconstruction was performed in either IMOD (Mastronarde and Held, 2017) or AreTomo (Zheng et al, 2022). In IMOD, four times binned tomograms were reconstructed using weighted back-projection, with a SIRT-like filter applied for visualization/segmentation. In AreTomo, four-time-binned tomograms were reconstructed using weighted-back projection. AreTomo reconstructed tomograms were denoised with CryoCare for visualization purposes.

### Determination of MT polarity, protofilament number, and end structures

In 25 thin tomograms with a good signal-to-noise ratio, individual MTs were manually traced in IMOD, and model points were imported into the software package Cylindra (Liu et al, 2024). In Cylindra, the protofilament number and slew were determined for each MT. Based on preceding literature (Foster et al, 2022), we assumed a clockwise protofilament slew is minus to plus end direction and a counterclockwise slew is plus to minus end direction. MT end structures were assigned based on previous literature (Gudimchuk and McIntosh, 2021). In short, blunt ends are straight ends with all PFs roughly the same length. Flared MT ends are where PFs are about the same length but not straight. Tapered ends have a long extension of a few PFs.

### Cryo-EM single particle analysis data collection and processing

Cryo-EM data of purified manchettes were collected on a 300 kV Titan Krios at the Netherlands Centre for Electron Nanoscopy (NeCEN). Three datasets were collected from three grids from three manchette preparations. Imaging conditions are summarized in Table EV1. Acquisition areas were identified manually, and images were collected semi-automatically in SerialEM. Frames were motion-corrected on the fly using Warp (Tegunov and Cramer, 2019) to monitor data quality during collection. Movies were drift-corrected and dose-weighted using “*Patch Motion Correction*” in cryoSPARC (Punjani et al, 2017). CTF parameters were estimated using “*Patch CTF Estimation”* in cryoSPARC. Manchette MTs were automatically traced from a total of 7598 micrographs using “*Filament Tracer”* in cryoSPARC. MT particles were then extracted from the micrographs (Fourier binning from 500 pixels to 200 pixels) using overlapping boxes with an 82.5 Å step size, corresponding to the length of an α/β-tubulin heterodimer. Two rounds of 2D classifications were used to discard junk particles and off-centered MT particles.

Next, *‘heterogeneous refinement’* of the remaining MT particles was performed using two reference models (13-protofilament (PF) and 14-PF MTs). Approximately 87% of the particles were classified as 13-PF MTs, and the 3D reconstruction revealed an oval-shaped MT. Only the 13-PF MTs were used for subsequent data processing, carried out in four major steps. (I) A 3D reconstruction was obtained using ‘helical refinement’ applying pseudo-helical symmetry (rise: 83.0 Å, twist: 0°). The result was further polished by ‘local refinement’. (II) Using pyem (DOI: 10.5281/zenodo.3576630) the alignment parameters were converted from CryoSPARC to RELION format, and then to FREALIGN format using customized python scripts (https://github.com/rui--zhang/Microtubule) (Zhang and Nogales, 2015; Valdez et al, 2024). (III) We used a previously described FREALIGN (Grigorieff, 2007) based procedure to determine the seam location for each MT particle (Zhang and Nogales, 2015; Valdez et al, 2024). During this step, alignment errors of the MT particles were minimized using the geometric relationship among neighboring MT particles. (IV) The refined particle set, featuring the correct seam assignment and α,β-tubulin register, was imported back to CryoSPARC and subjected to local refinement. The 3D reconstruction revealed an apparent MIP density between PF1 and PF13.

Four additional steps were carried out to further improve the quality of the MIP density. (I) *‘3D Classification’* was performed in CryoSPARC (6 classes, filter resolution 8 Å) using a cylindrical mask (diameter 50 Å, length 344 Å) covering MIP densities. A subset of around 20% of the MT particles with strong MIP density was selected for further processing. (II) Next, the MT particles were re-extracted (Fourier binning from 500 pixels to 400 pixels) with an 82.5 Å step size, followed by a local refinement, local CTF refinement, and another round of local refinement. This process yielded a MT C1 reconstruction at 3.2 Å resolution (FSC cutoff = 0.143) from 971,980 MT particles. (III) Another *‘3D Classification’* was performed using CryoSPARC (6 classes, filter resolution 6 Å), applying a soft-edged mask that covered only one MIP density within the 8-nm repeat length. The 3D classification identified two classes with MIP densities that indicate relative movement between the MIP and the MT wall. (IV) The two classes were further locally refined using a short cylindrical mask (diameter 110 Å, length 120 Å) covering one MIP and two protofilaments, resulting in a local resolution of around 3.2 Å.

### Protein identification and model building

The SPACA9 protein was identified using ModelAngelo (Jamali et al, 2024), via an “hmm_search” against the entire rat proteome (UP000002494_10116.fasta). This assignment was validated through careful examination of the correspondence between the atomic model and side-chain densities (for density examples, see Fig. EV2C).

The MNMIP1 was identified using a docking-based approach. We first generated a library of individual domains from AlphaFold-predicted structures (Abramson et al, 2024; Jumper et al, 2021) corresponding to the top 1000 proteins identified in M/S analysis of isolated manchettes (Judernatz et al, 2025), using scripts from the DomainFit package (Gao et al, 2024). Each domain was docked into the cryo-EM density using ChimeraX (Goddard et al, 2018), and the fits were quantitatively evaluated (Gao et al, 2024).

The positions of tubulin dimers, SPACA9, and MNMIP1 were manually adjusted by rigid-body fitting in ChimeraX, followed by real-space refinement in Coot (Emsley et al, 2010) and PHENIX (Afonine et al, 2018). The binding interfaces were analyzed on the PISA website (Krissinel and Henrick, 2007).

### SPACA9 and MNMIP1 cloning and expression in tissue culture cells

Synthetic genes for MNMIP1 and SPACA9 (GenScript) were cloned into pEGFP-C1 and pmCherry-C1 vectors, modified with a StrepII tag, through conventional molecular cloning and PCR-based cloning. U2OS cells were cultured in Dulbecco’s modified Eagle medium supplemented with 10% fetal calf serum and 1% (v/v)penicillin/streptomycin. The cells were routinely tested for mycoplasma contamination using the MycoAlert TM Mycoplasma Detection Kit (Lonza). For overexpression of the EGFP-MNMIP1 and mCherry-SPACA9 constructs, cells were transiently transfected using Fugene6 (Promega) for 24 h. For the nocodazole sensitivity assay, the cells were treated with 10 μM nocodazole (Sigma-Aldrich) for 15 min. The cells were seeded on glass coverslips and were fixed post-transfection with pre-cooled methanol (−20 °C) for 10 min. Subsequently, the cells were washed 2× with PBS for 5 min and incubated in blocking buffer (2% bovine serum albumin and 0.05% Tween-20 in PBS) for 45 min. Following, cells were incubated with primary antibodies diluted in blocking buffer (mouse anti-GFP (Roche) 1:300, chicken anti-GFP (Aves labs) 1:300, chicken anti-mCherry (Abcam) 1:300, mouse anti-α-tubulin (Sigma-Aldrich) 1:500) for 1 h at room temperature. After three subsequent washes of 5 min with 0.05% Tween-20 in PBS, cells were incubated with secondary antibodies diluted in blocking buffer (goat anti-mouse – and chicken IgG Alexa Fluor -488, -594, -647 (Invitrogen) 1:300 dilution) for 1 h at room temperature, followed by another 3 × 5 min washes with 0.05% Tween-20 in PBS. Finally, cells were dried in 96% ethanol and mounted on glass slides with ProLong Diamond Antifade mounting medium (Thermo Fisher Scientific). Fixed and stained U2OS cells were imaged using an upright Axio Imager Z2 LSM900 microscope (Zeiss), equipped with a Plan-Apochromat 63×/1.4 oil objective and two multialkali PMT detectors. The microscope was controlled using the ZEN Blue v3.7 software.

### Pull-down assay

HEK293T cells were transiently transfected with the constructs of SPACA9 and MNMIP1 using polyethylenimine (Polysciences). Following a 24-h incubation, cells were collected in ice-cold 1× PBS and lysed on ice for 30 min in lysis buffer (10 mM Tris-HCl pH 7.5, 150 mM NaCl, 0.5 mM EDTA, and 0.5% IGEPAL CA-630) supplemented with PhosSTOP and EDTA-free protease inhibitors (Roche). The resulting lysates were cleared by centrifugation at 17,000 × g for 20 min at 4 °C and diluted with 300 μL dilution buffer (10 mM Tris-HCl pH 7.5, 150 mM NaCl, and 0.5 mM EDTA) supplemented with the same inhibitors. Co-immunoprecipitation was performed with ChromoTek GFP-Trap magnetic beads M-270 (Proteintech) by following the manufacturer’s instructions and using buffers advised by the company. Diluted lysate was incubated rotating end-over-end for 45 min at 4 °C with wash buffer equilibrated beads. Subsequently, beads were washed four times with 500 μL of wash buffer (10 mM Tris-HCl pH 7.5, 150 mM NaCl, 0.5 mM EDTA, and 0.05% IGEPAL CA-630). The bound complexes were eluted by heating at 95 °C for 5 min in 2× Laemmli sample buffer (0.125 M Tris-HCl pH 7.5, 20% glycerol, 10% 2-mercaptoethanol, 0.02% bromophenol blue, and 0.2 M DTT). Proteins were separated on an 8% SDS-PAGE and transferred to Amersham Protran Premium 0.45-μm NC nitrocellulose membrane (Cytiva) by wet transfer at 30 V constant overnight at 4 °C in transfer buffer (0.2 M Tris-HCl, 2 M glycine, 20% methanol, and 0.01% SDS). PageRuler Plus Prestained Protein Ladder (Thermo Fisher Scientific) was used as the molecular weight marker. Membranes were blocked in blocking buffer (2% BSA diluted in 1× PBS and 0.05% Tween-20) for 90 min at room temperature. The membranes were then incubated with primary antibodies (rabbit anti-RFP, 1:2000, Rockland; mouse anti-GFP, 1:2000, Sigma-Aldrich) for 1 h, followed by secondary antibodies (donkey anti-mouse IRDye 680CW and donkey anti-rabbit IRDye 800CW, 1:10,000, LI-COR) for an additional hour. Both antibody steps were performed with constant rotation at room temperature. Membranes were thoroughly washed in between and after incubation with antibodies with wash buffer (1× PBS and 0.05% Tween-20) and imaged with an Odyssey CLx Infrared Imaging System (LI-COR).

## Supplementary information

Table EV1

Peer Review File

Movie EV1

Movie EV2

Movie EV3

Movie EV4

Source data Fig. 1

Source data Fig. 2

Source data Fig. 3

Source data Fig. 4

Source data Fig. 5

Expanded View Figures

## Data Availability

The cryo-EM map generated during this study has been deposited in the Electron Microscopy Data Bank (EMDB; https://wwwdev.ebi.ac.uk/pdbe/emdb) with accession code EMD-54641. The model generated during this study has been deposited in the Protein Data Bank (PDB; http://www.wwpdb.org) with accession code PDB ID 9S7G. The source data of this paper are collected in the following database record: biostudies:S-SCDT-10_1038-S44318-026-00833-w.
