# Supplementary material for: SPACA9 and MNMIP1 bridge the seam of spermatid manchette microtubules
Source: EMBO J. 2026 Jun 12;45(14):5024–45. doi: 10.1038/s44318-026-00833-w (PMC13373224; doi:10.1038/s44318-026-00833-w)
Supplement: Supplementary file 1 — Table EV1 [file 44318_2026_833_MOESM1_ESM.docx]

| **Microscope** | **Titan Krios** |
| --- | --- |
| Voltage (kV) | 300 |
| Nominal magnification | 79000 X |
| Detector | Gatan K3 |
| Energy filter | BioQuantum |
| Slit width (eV) | 20 |
| Imaging mode | super-resolution |
| Pixel size (Å) | 1.06 |
| Cumulative exposure (e^-^/Å^2^) | 50 |
| Exposure per frame (e^-^/Å^2^) | ~1 |
| Frames | 50 |
| Defocus range (μm) | -0.75 to -2.5 |
| Movies collected | 7598 |
| Particles (initial) | 1900466 |
| Particles (final) | 173602 |
| Symmetry | C1 |
| Map resolution (Å) | 3.2 |

**Table EV1: SPA data collection and data processing**
