# Supplementary material for: SPACA9 and MNMIP1 bridge the seam of spermatid manchette microtubules
Source: EMBO J. 2026 Jun 12;45(14):5024–45. doi: 10.1038/s44318-026-00833-w (PMC13373224; doi:10.1038/s44318-026-00833-w)
Supplement: Supplementary file 4 — Movie EV2 [file 44318_2026_833_MOESM4_ESM.zip › Movie EV2/Expanded View Movie 2.docx]

***Movie EV2.*** *Three-dimensional STED reconstruction of a mouse spermatid. The cell is labeled for the nucleus (DAPI, blue), microtubules (green), and CAMSAP1 (orange). Scale bar represents 2 µm.*
