# Supplementary figures and images for: SPACA9 and MNMIP1 bridge the seam of spermatid manchette microtubules
Source: EMBO J. 2026 Jun 12;45(14):5024–45. doi: 10.1038/s44318-026-00833-w (PMC13373224; doi:10.1038/s44318-026-00833-w)

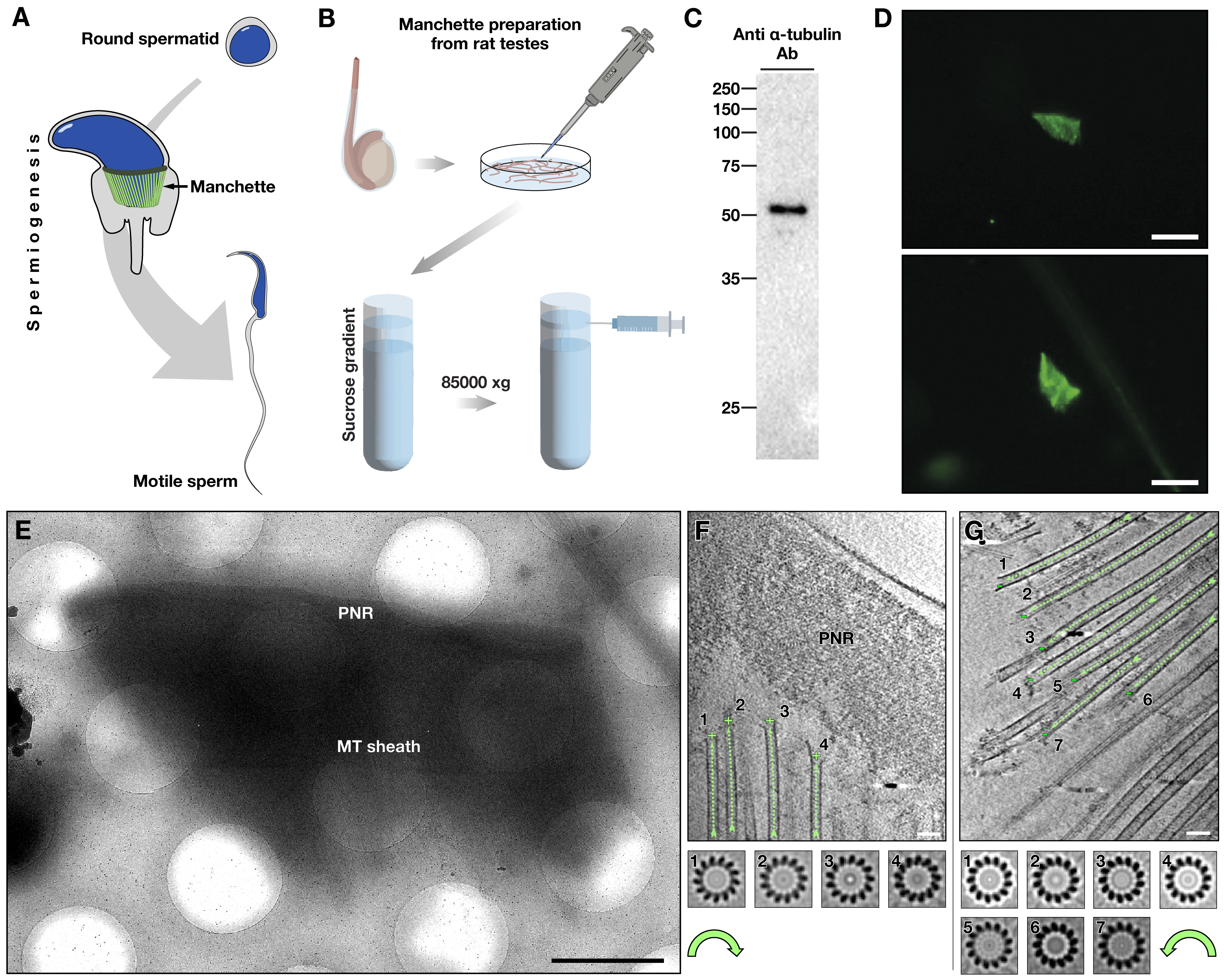

Supplement: Supplementary file 7 — Source data Fig. 1 [file 44318_2026_833_MOESM7_ESM.zip › Fig1/Figure 1.tif]

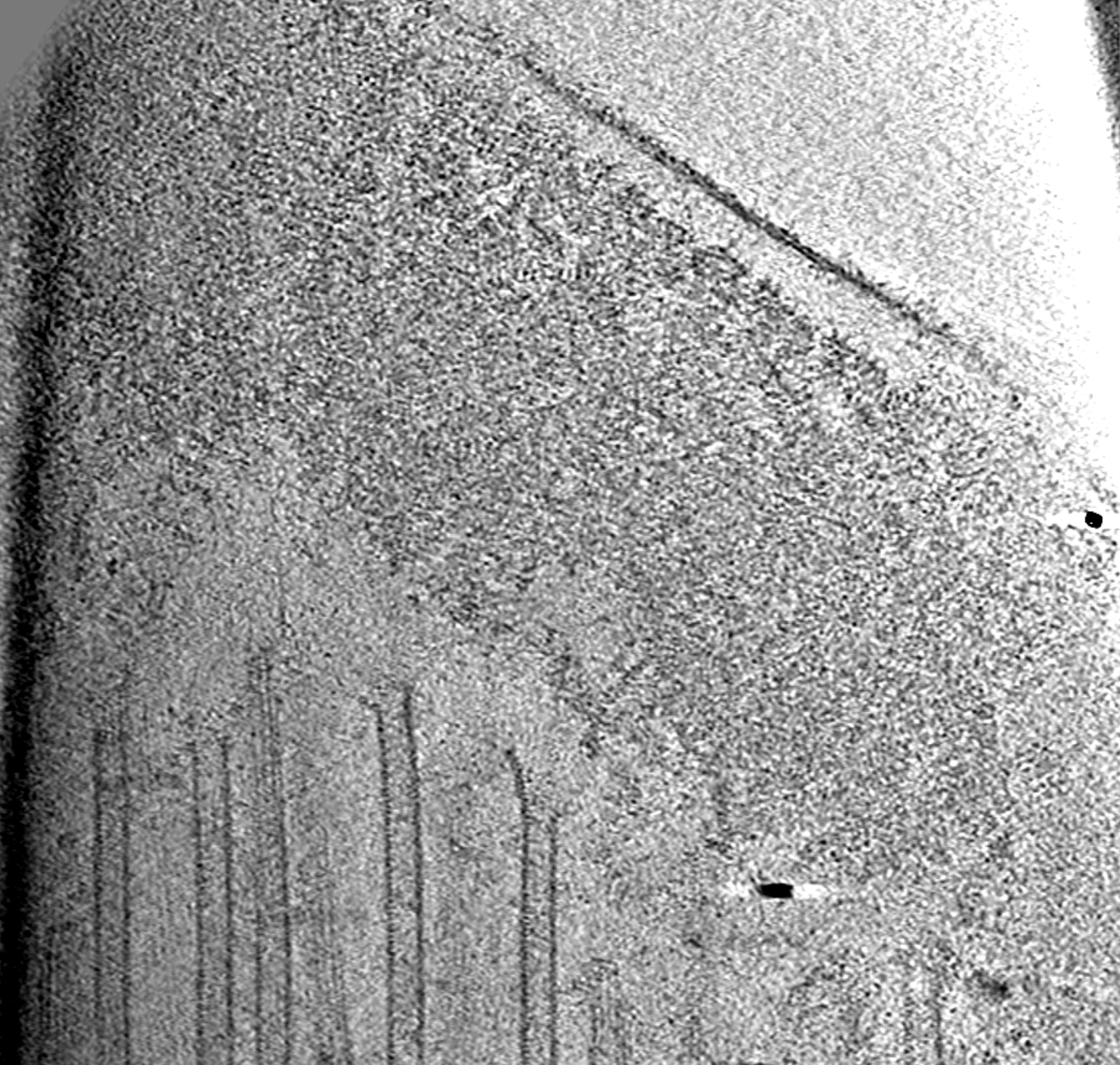

Supplement: Supplementary file 7 — Source data Fig. 1 [file 44318_2026_833_MOESM7_ESM.zip › Fig1/PanelF/Tomo123_slice.png]

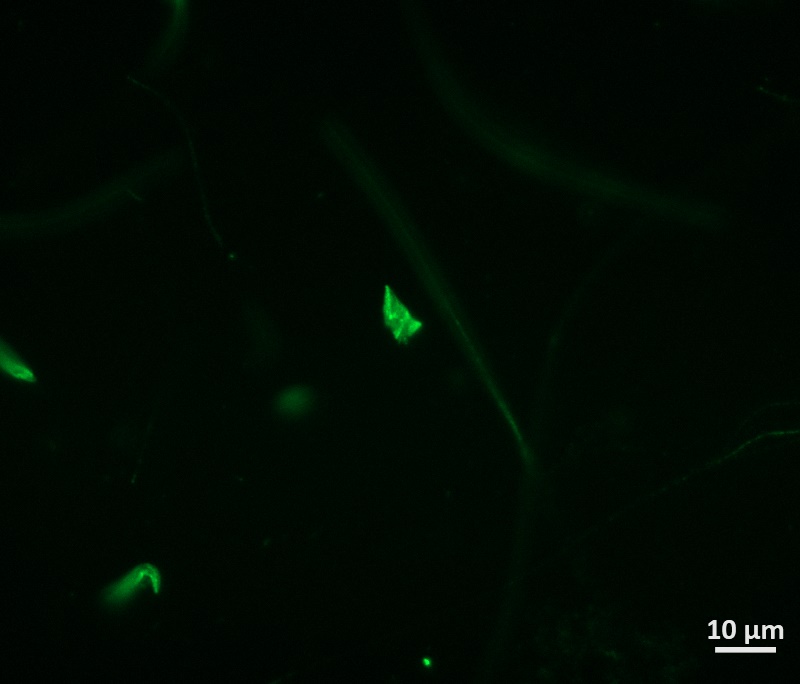

Supplement: Supplementary file 7 — Source data Fig. 1 [file 44318_2026_833_MOESM7_ESM.zip › Fig1/PanelD/Snap-690.jpg]

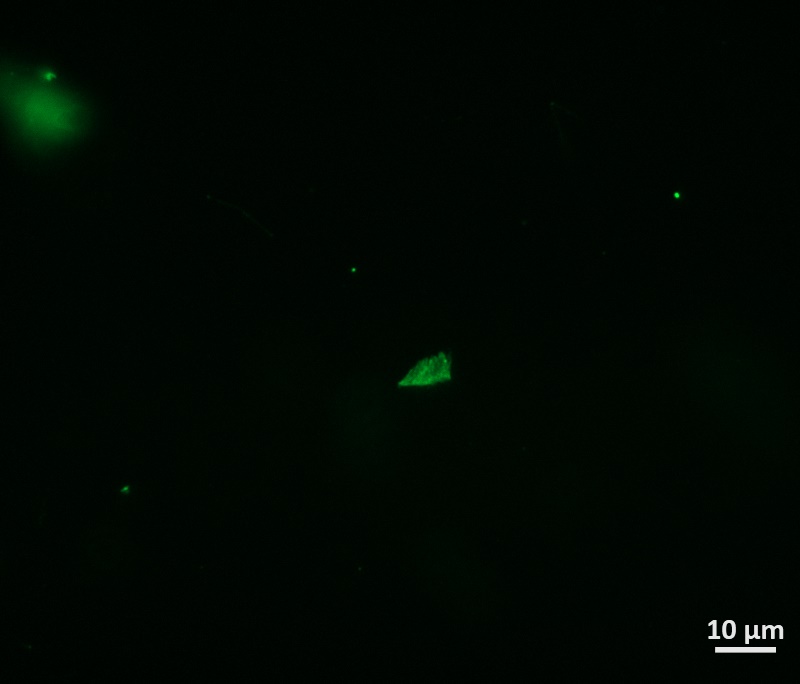

Supplement: Supplementary file 7 — Source data Fig. 1 [file 44318_2026_833_MOESM7_ESM.zip › Fig1/PanelD/Snap-688.jpg]

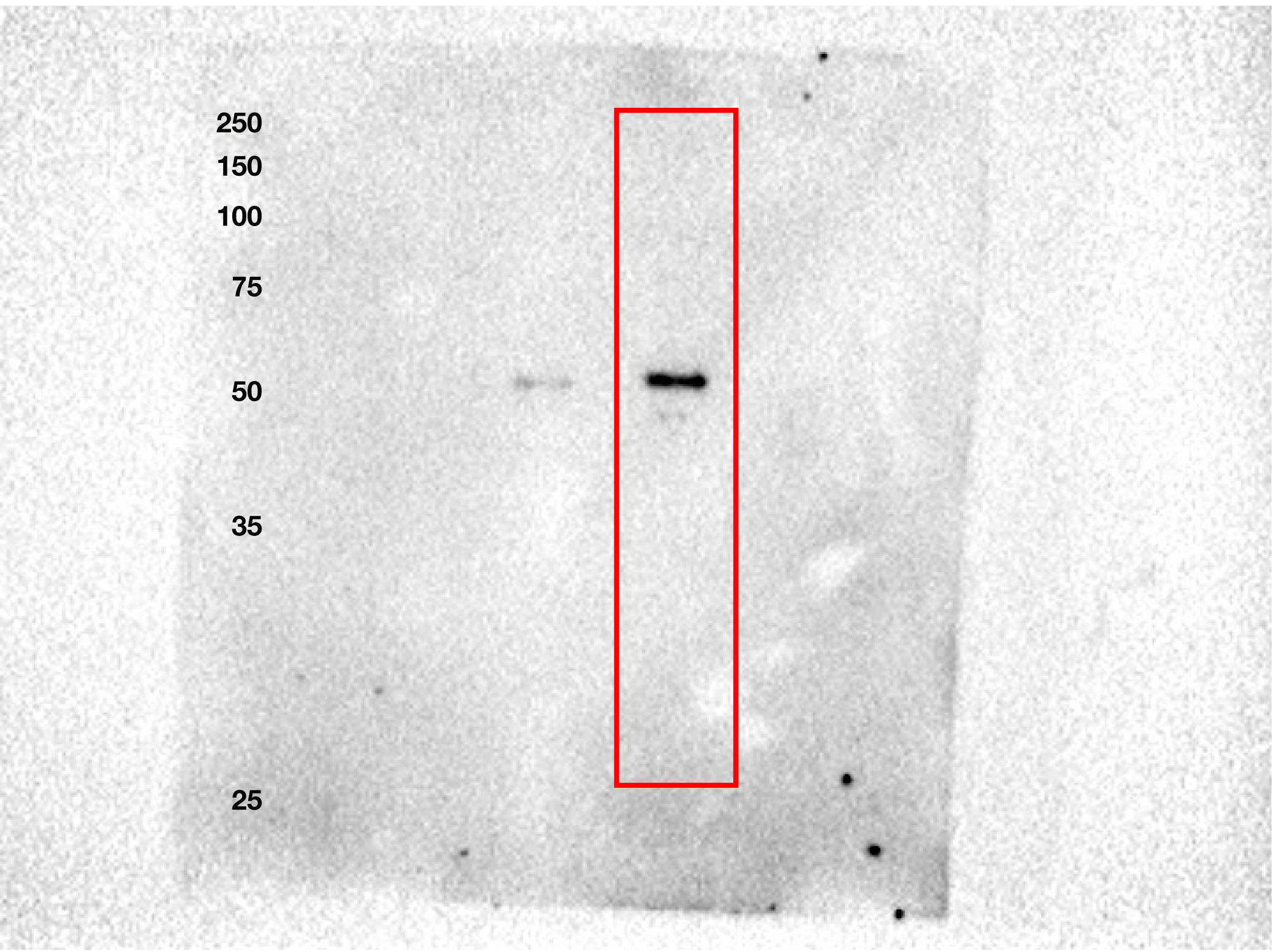

Supplement: Supplementary file 7 — Source data Fig. 1 [file 44318_2026_833_MOESM7_ESM.zip › Fig1/PanelC/Blot.tif]

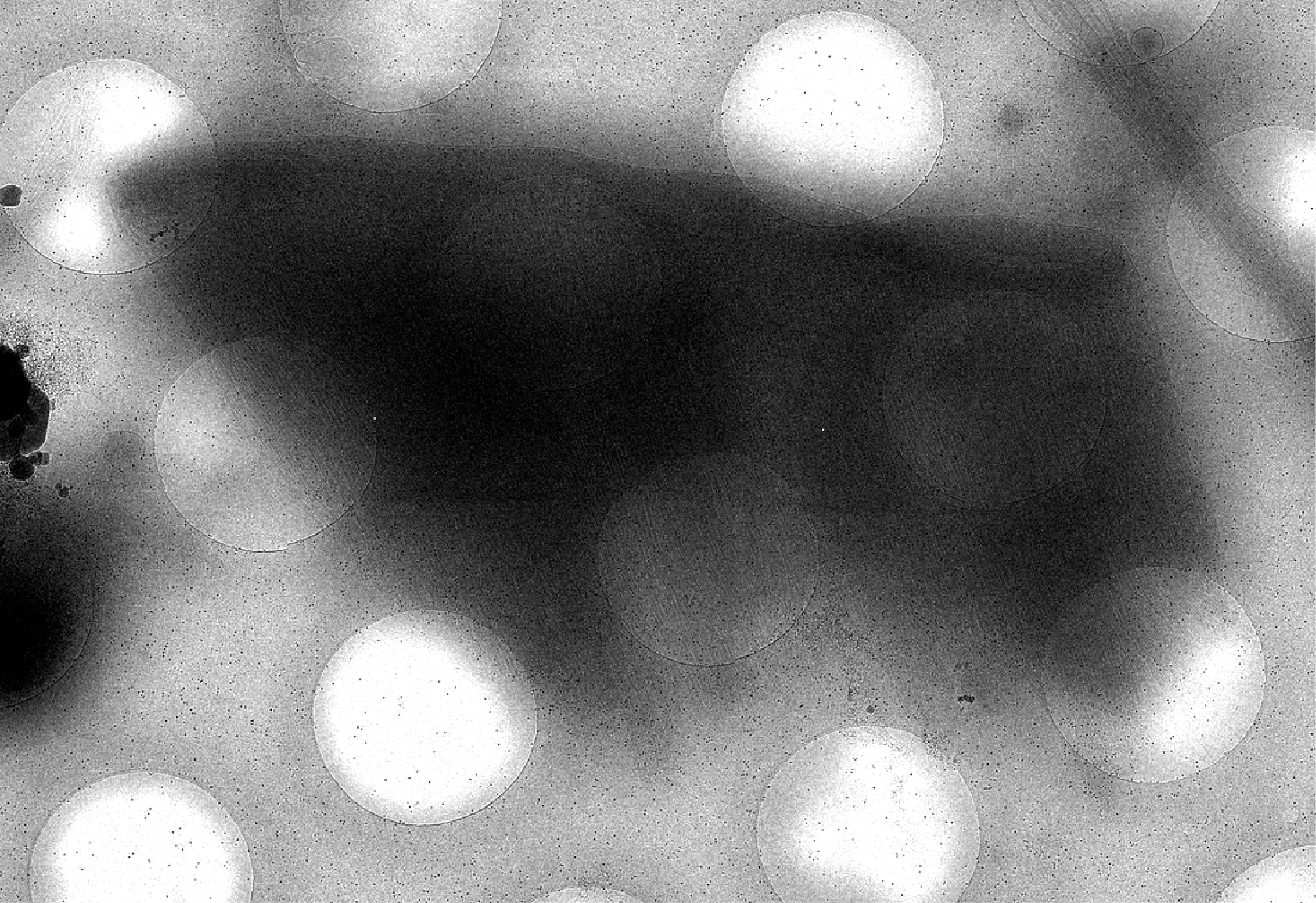

Supplement: Supplementary file 7 — Source data Fig. 1 [file 44318_2026_833_MOESM7_ESM.zip › Fig1/PanelE/Manchette_Overview.tif]

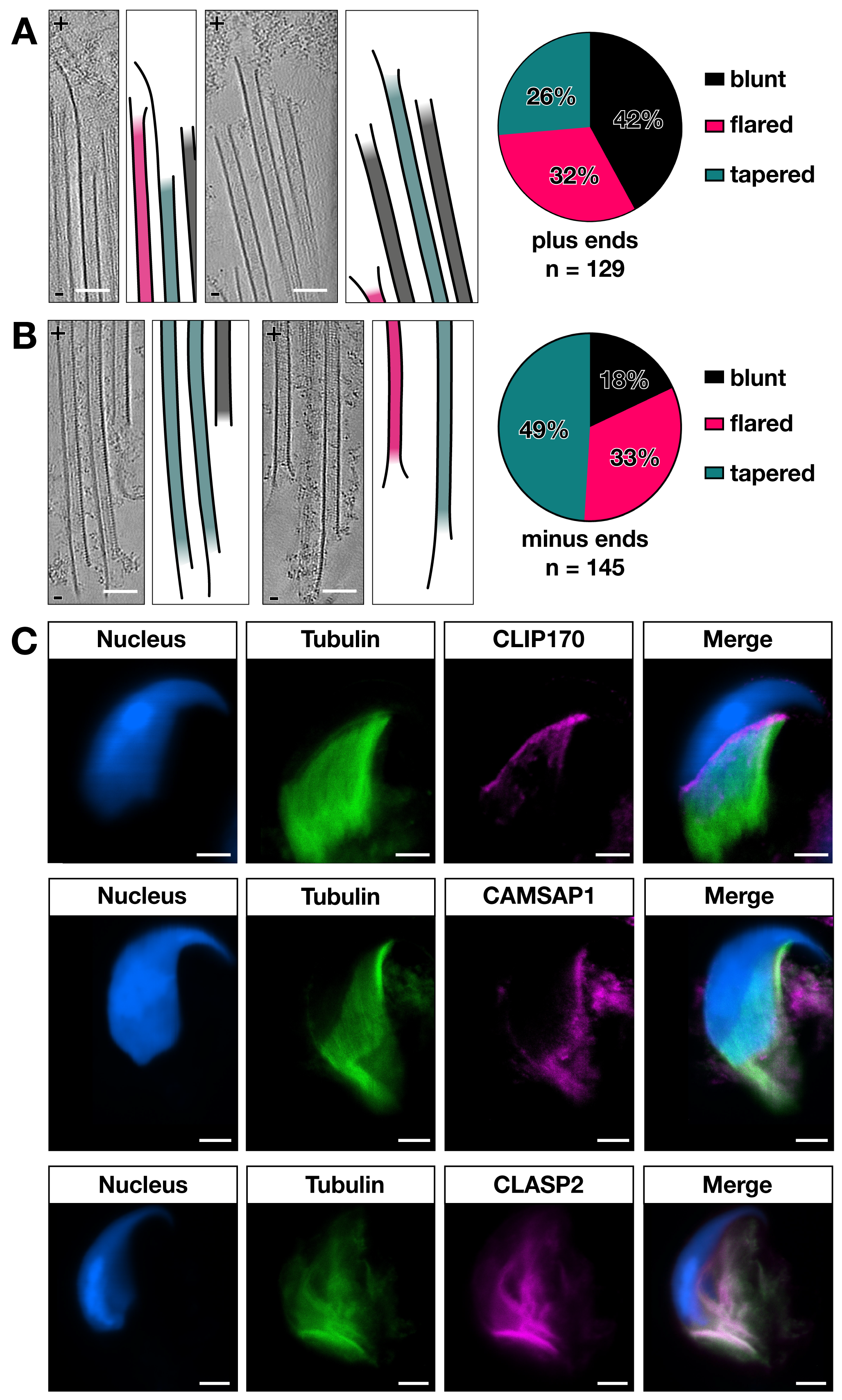

Supplement: Supplementary file 8 — Source data Fig. 2 [file 44318_2026_833_MOESM8_ESM.zip › Fig2/Figure 2.tif]

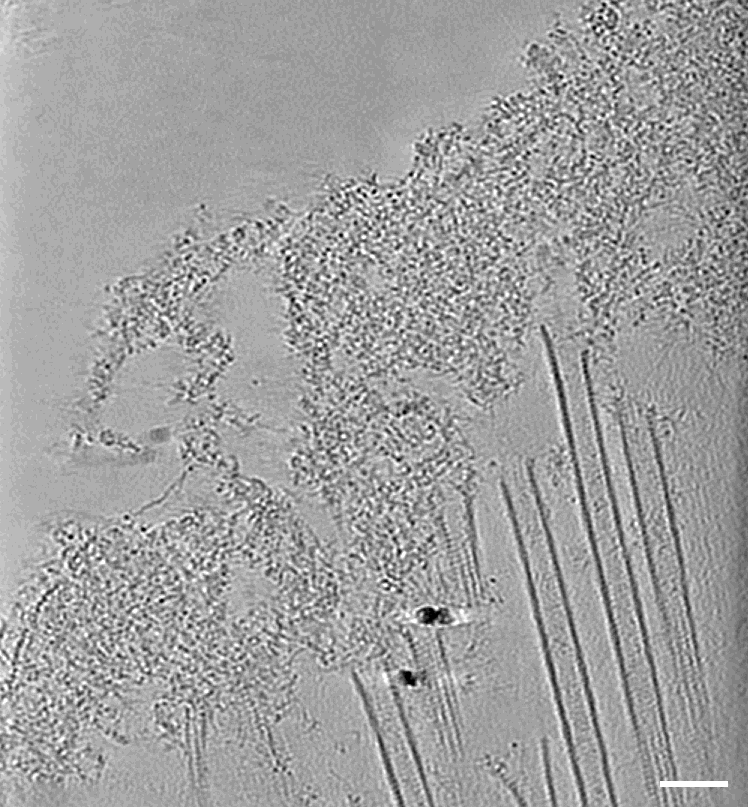

Supplement: Supplementary file 8 — Source data Fig. 2 [file 44318_2026_833_MOESM8_ESM.zip › Fig2/PanelA/Tomo104_slice.tif]

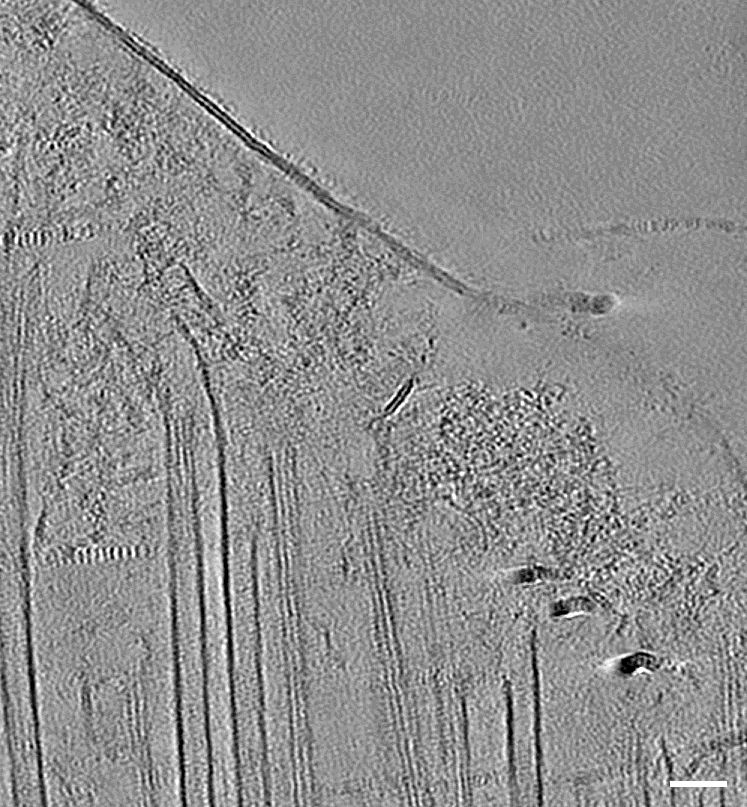

Supplement: Supplementary file 8 — Source data Fig. 2 [file 44318_2026_833_MOESM8_ESM.zip › Fig2/PanelA/Tomo072_slice.tif]

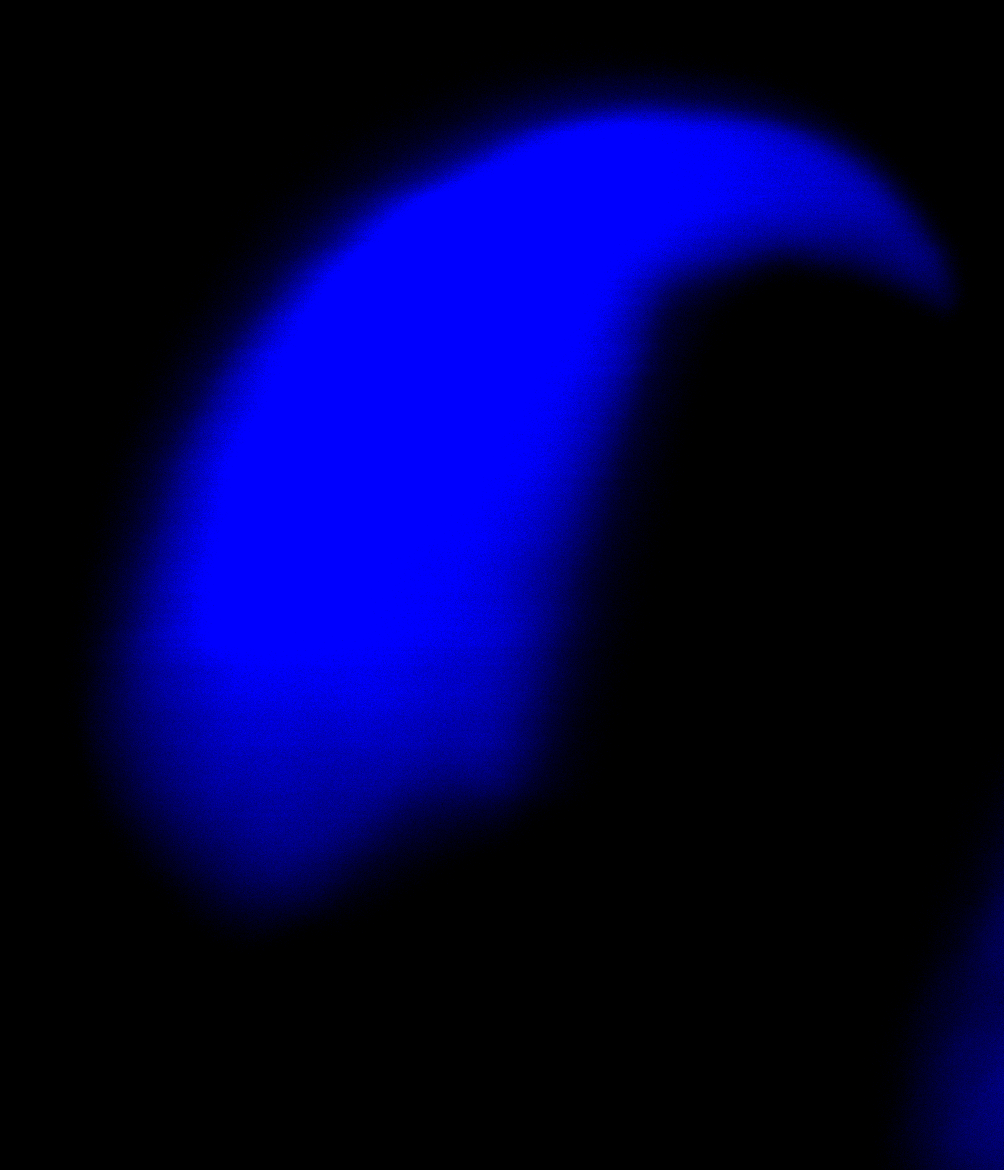

Supplement: Supplementary file 8 — Source data Fig. 2 [file 44318_2026_833_MOESM8_ESM.zip › Fig2/PanelC/Raw_STED_Images/CLIP1/Clip1_Blue_Nucleus.tif]

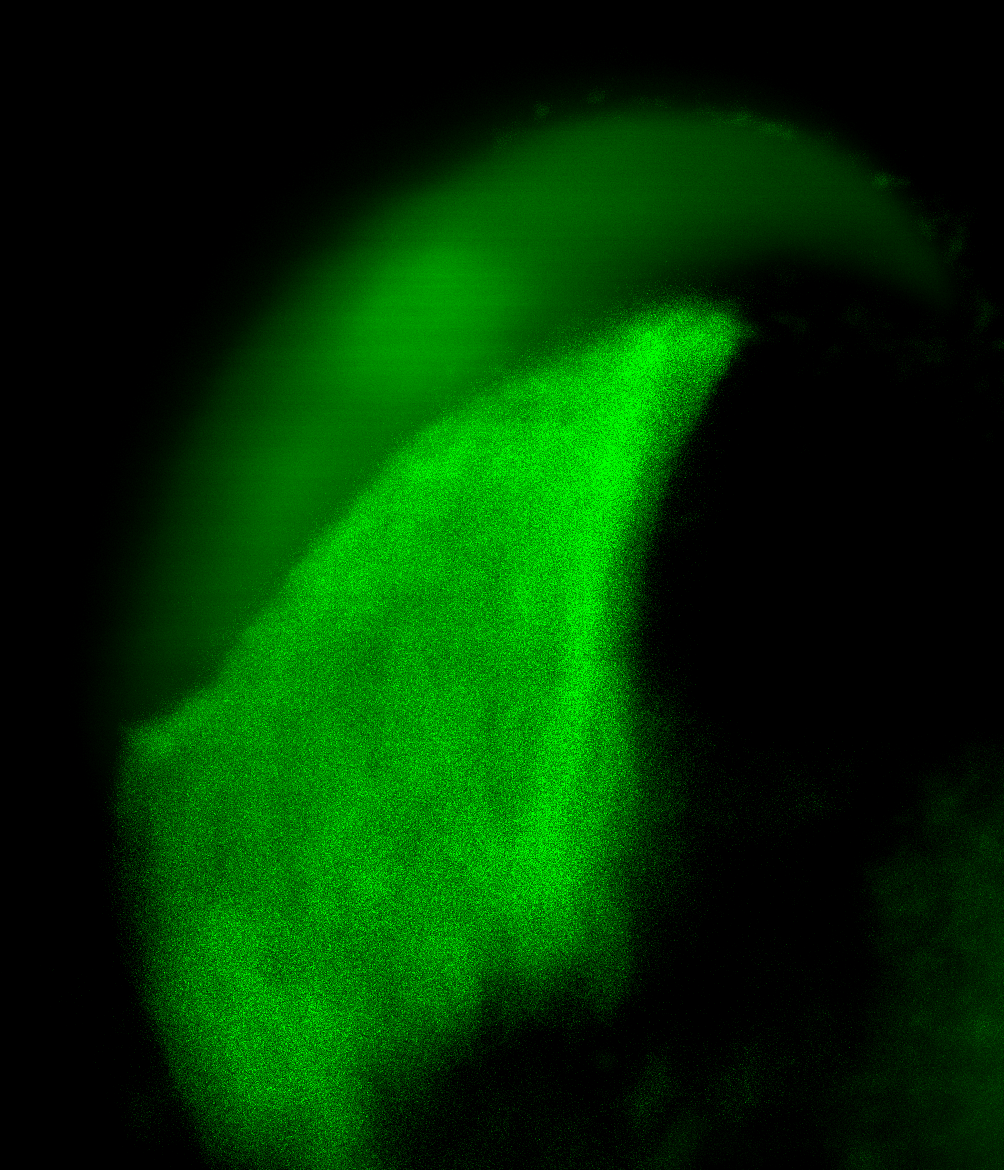

Supplement: Supplementary file 8 — Source data Fig. 2 [file 44318_2026_833_MOESM8_ESM.zip › Fig2/PanelC/Raw_STED_Images/CLIP1/Clip1_Green_Tub.tif]

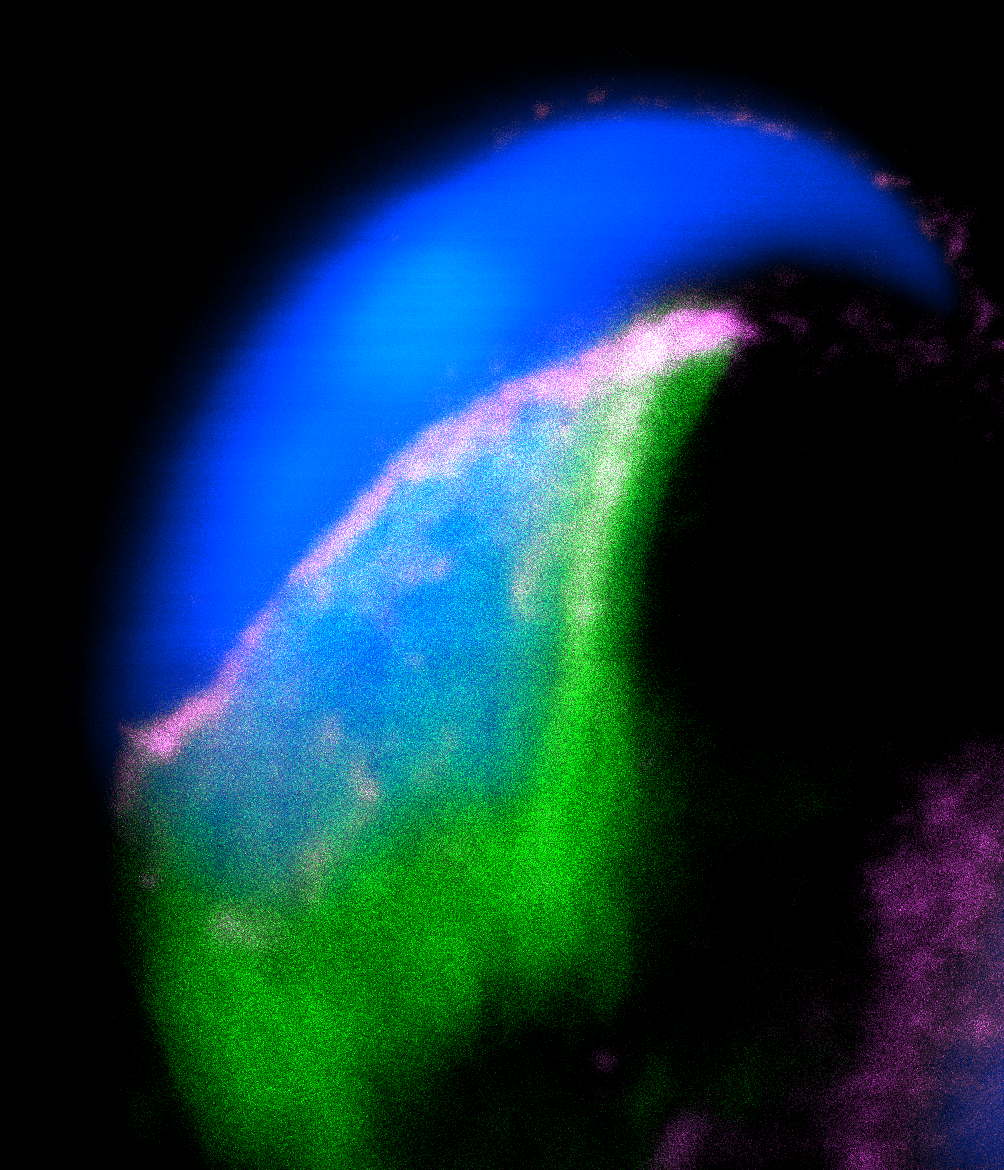

Supplement: Supplementary file 8 — Source data Fig. 2 [file 44318_2026_833_MOESM8_ESM.zip › Fig2/PanelC/Raw_STED_Images/CLIP1/Clip1_merge.tif]

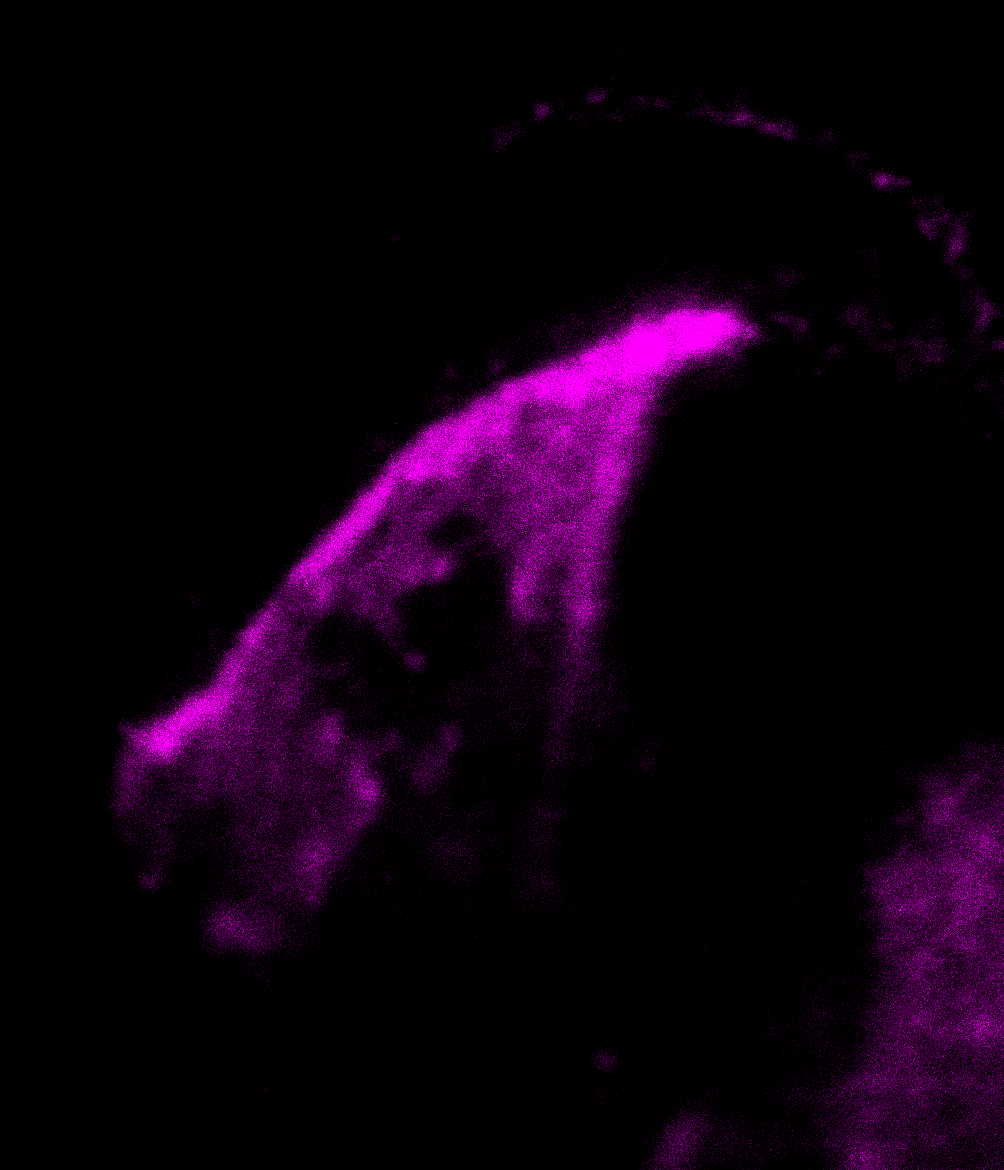

Supplement: Supplementary file 8 — Source data Fig. 2 [file 44318_2026_833_MOESM8_ESM.zip › Fig2/PanelC/Raw_STED_Images/CLIP1/Clip1_Magenta_Clip1.tif]

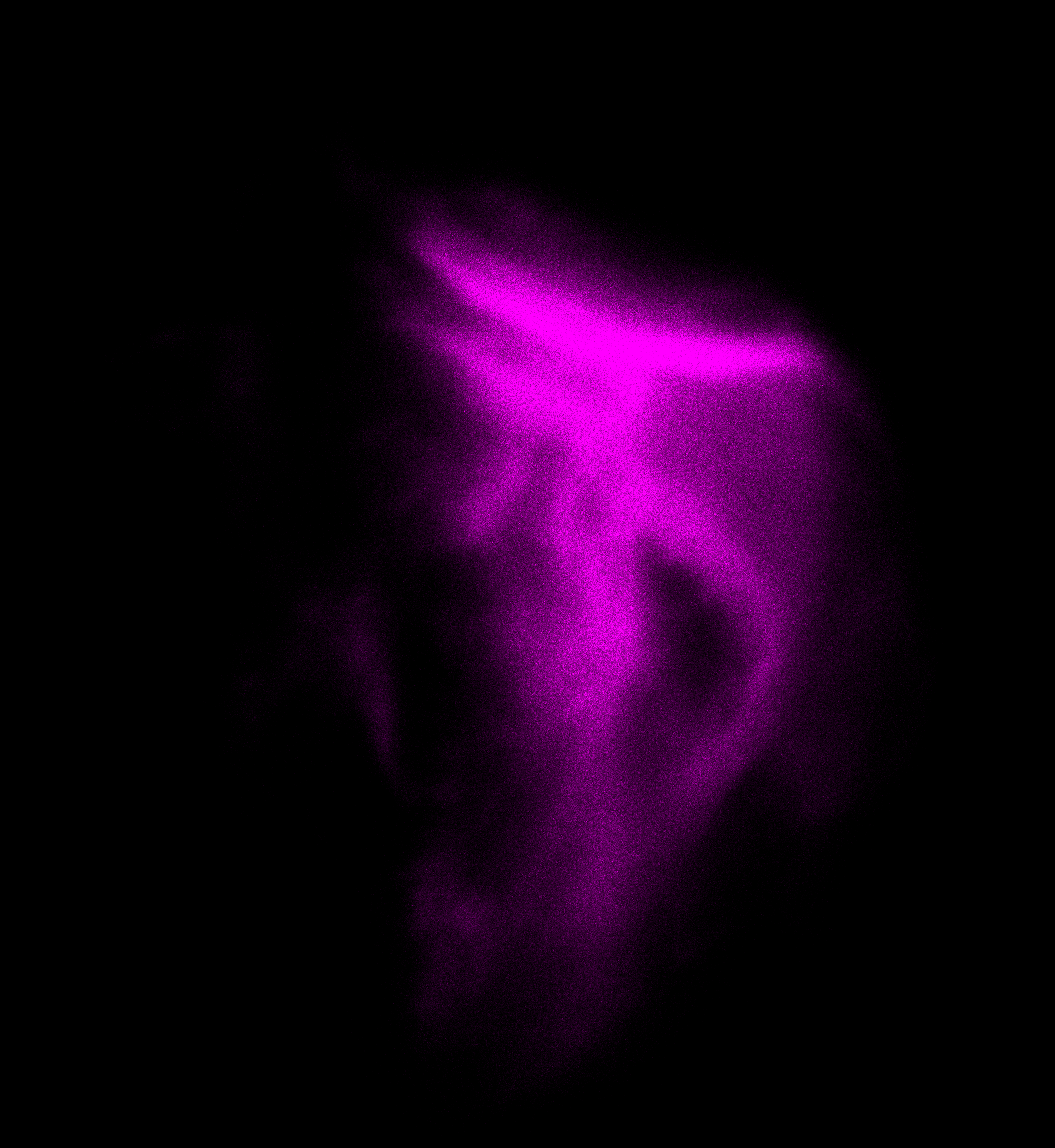

Supplement: Supplementary file 8 — Source data Fig. 2 [file 44318_2026_833_MOESM8_ESM.zip › Fig2/PanelC/Raw_STED_Images/CLASP2/CLASP2_Magenta_CLASP2.tif]

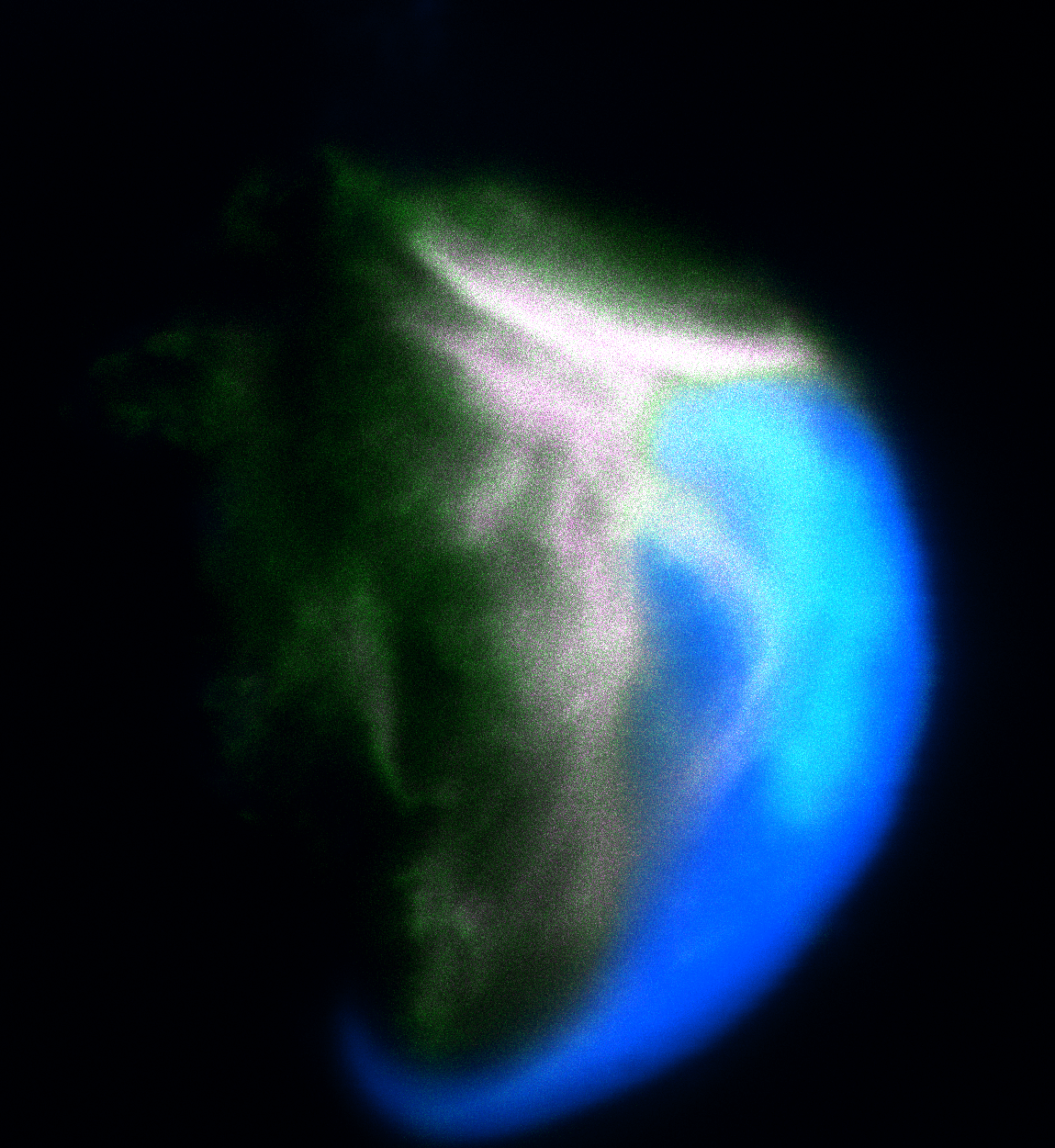

Supplement: Supplementary file 8 — Source data Fig. 2 [file 44318_2026_833_MOESM8_ESM.zip › Fig2/PanelC/Raw_STED_Images/CLASP2/CLASP2_merge.tif]

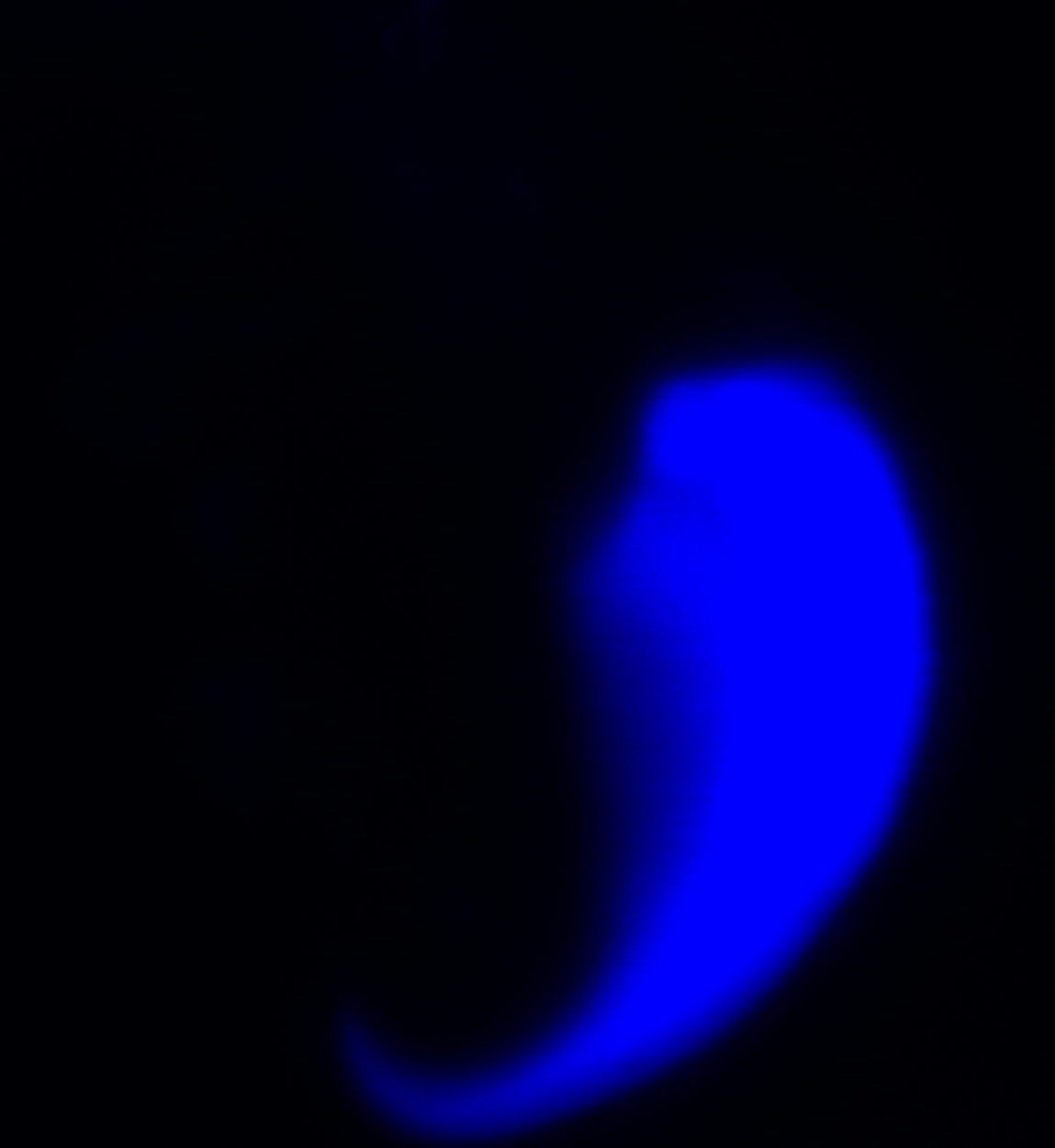

Supplement: Supplementary file 8 — Source data Fig. 2 [file 44318_2026_833_MOESM8_ESM.zip › Fig2/PanelC/Raw_STED_Images/CLASP2/CLASP2_Blue_Nucleus.tif]

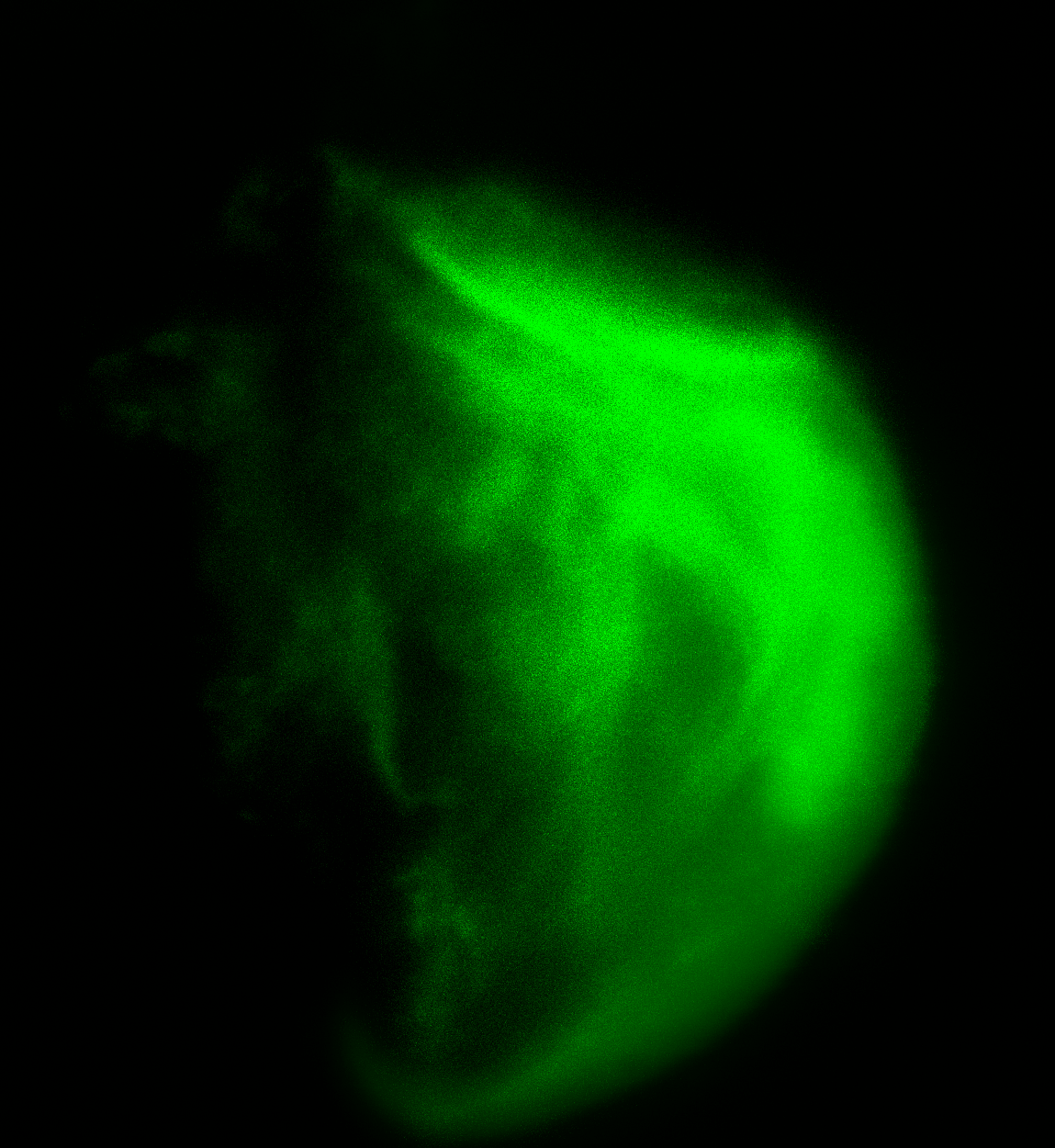

Supplement: Supplementary file 8 — Source data Fig. 2 [file 44318_2026_833_MOESM8_ESM.zip › Fig2/PanelC/Raw_STED_Images/CLASP2/CLASP2_Green_Tub.tif]

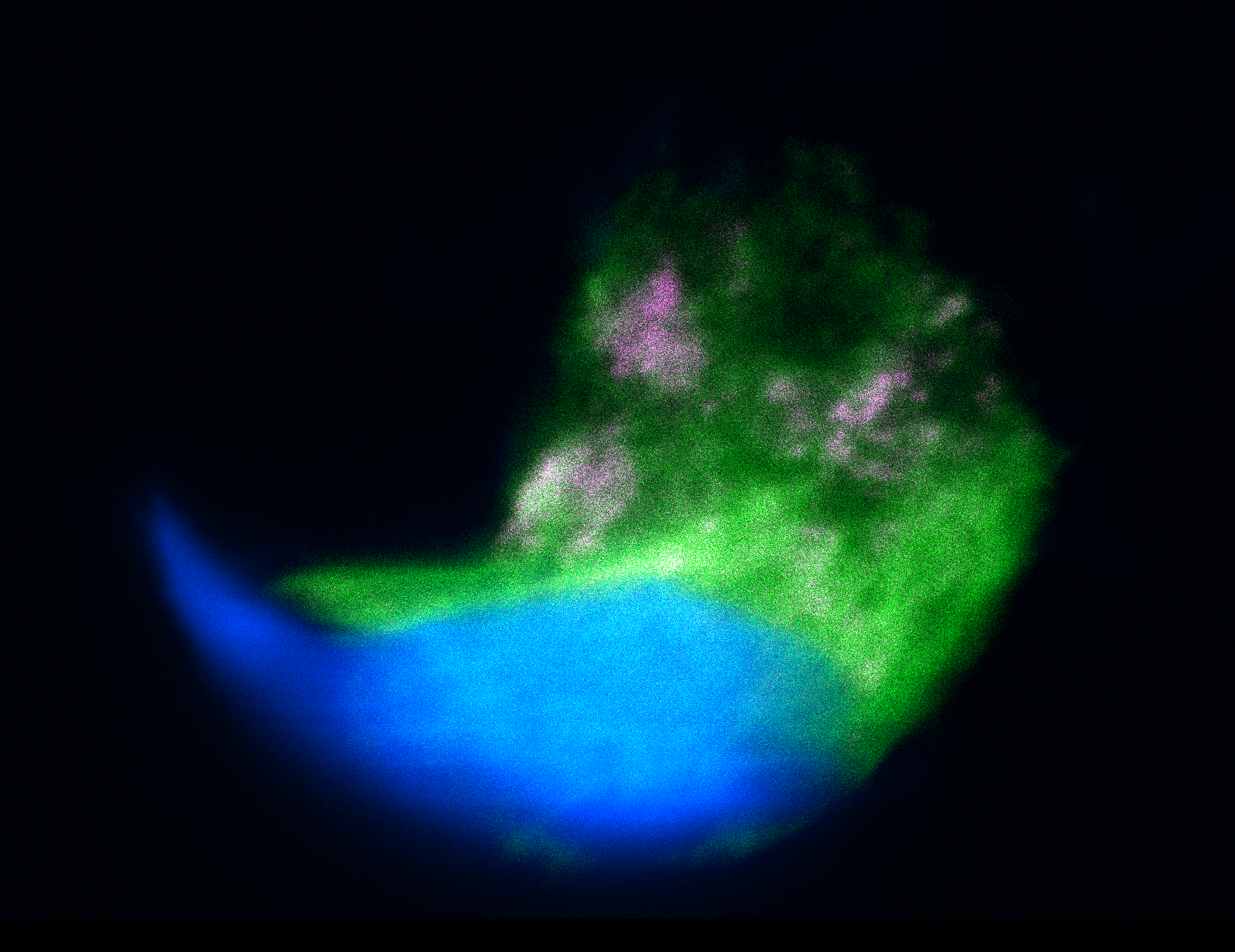

Supplement: Supplementary file 8 — Source data Fig. 2 [file 44318_2026_833_MOESM8_ESM.zip › Fig2/PanelC/Raw_STED_Images/CAMSAP1/CAMSAP1_merge.tif]

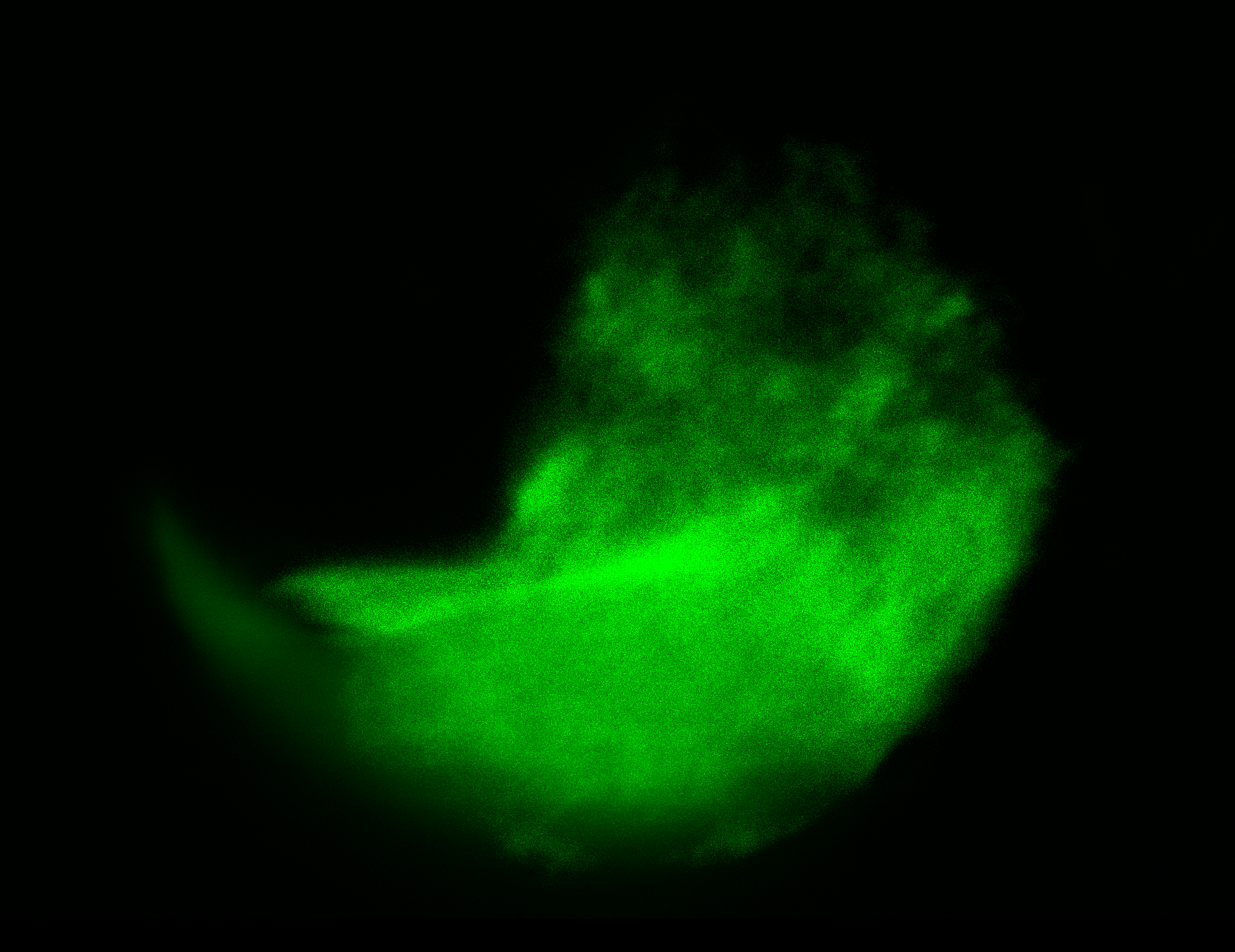

Supplement: Supplementary file 8 — Source data Fig. 2 [file 44318_2026_833_MOESM8_ESM.zip › Fig2/PanelC/Raw_STED_Images/CAMSAP1/CAMSAP1_Green_Tub.tif]

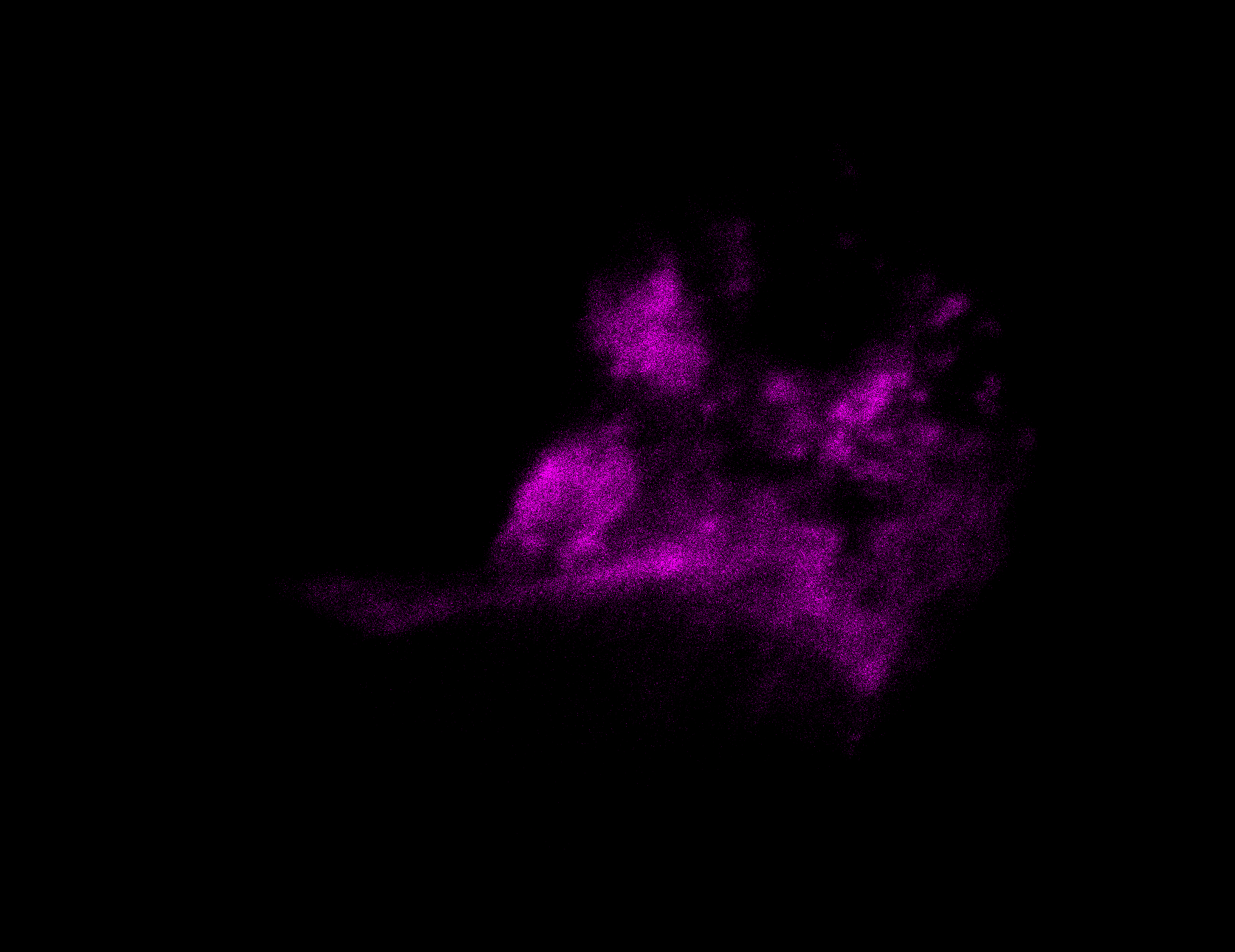

Supplement: Supplementary file 8 — Source data Fig. 2 [file 44318_2026_833_MOESM8_ESM.zip › Fig2/PanelC/Raw_STED_Images/CAMSAP1/CAMSAP1_Magenta_CAMSAP1.tif]

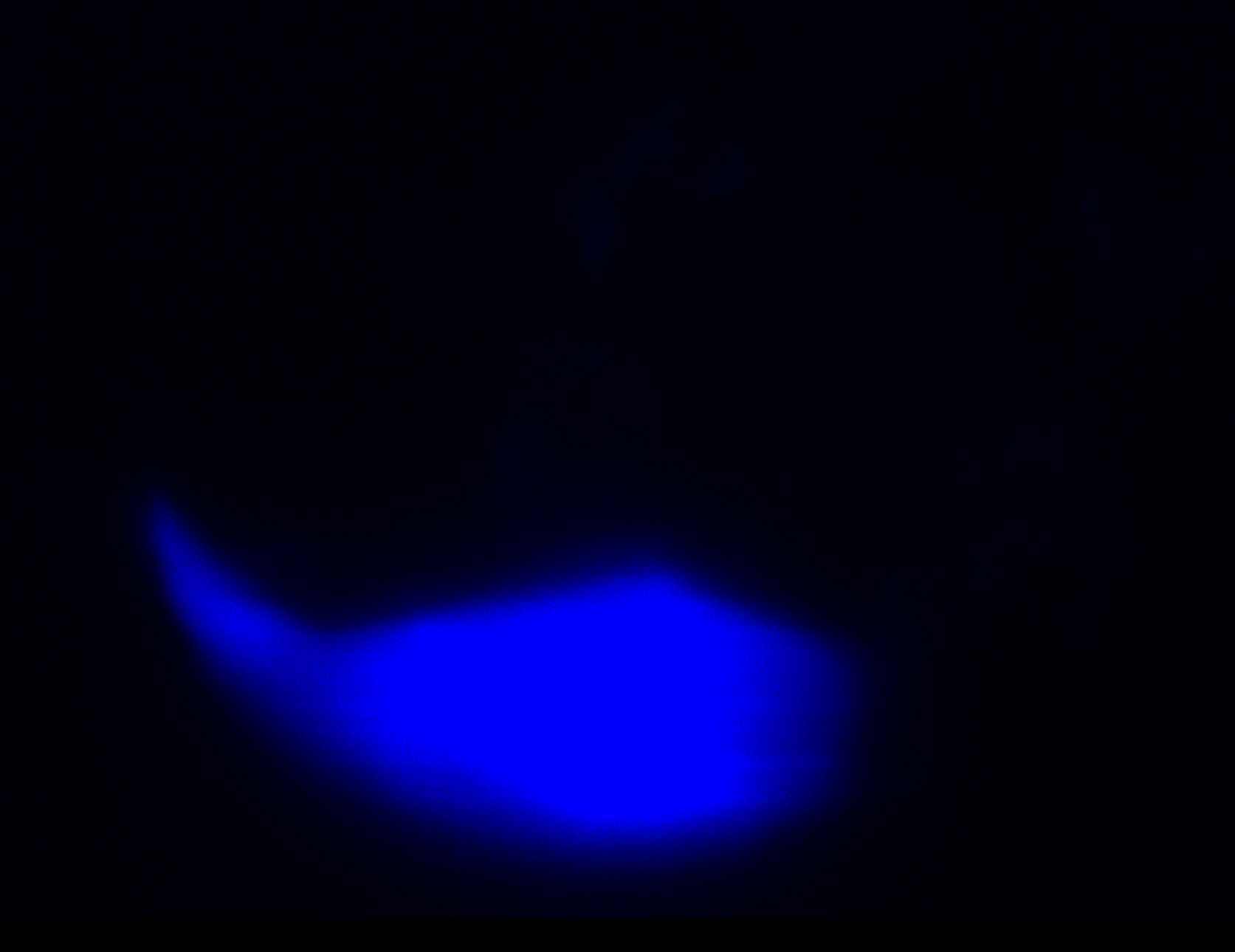

Supplement: Supplementary file 8 — Source data Fig. 2 [file 44318_2026_833_MOESM8_ESM.zip › Fig2/PanelC/Raw_STED_Images/CAMSAP1/CAMSAP1_Blue_Nuc.tif]

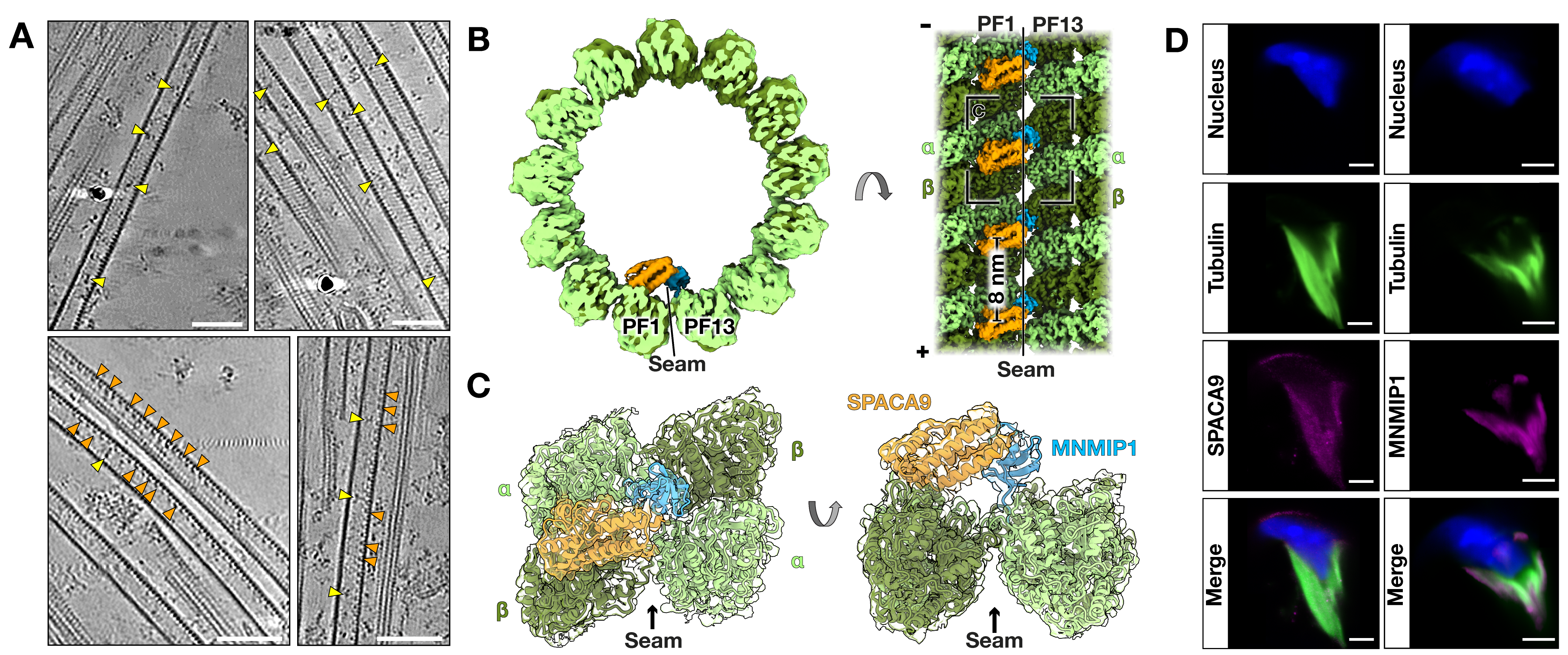

Supplement: Supplementary file 9 — Source data Fig. 3 [file 44318_2026_833_MOESM9_ESM.zip › Fig3/Figure 3.tif]

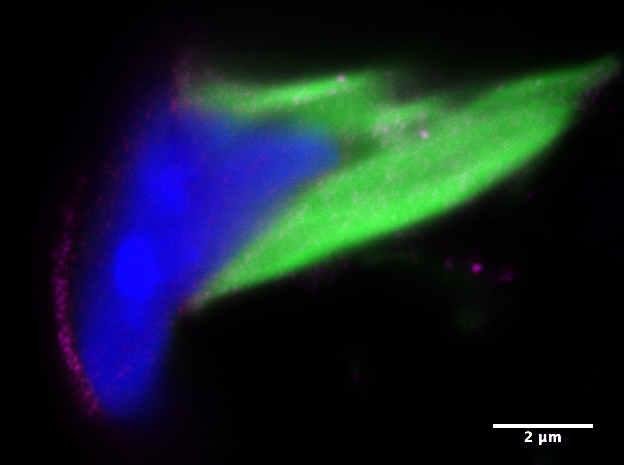

Supplement: Supplementary file 9 — Source data Fig. 3 [file 44318_2026_833_MOESM9_ESM.zip › Fig3/PanelD/SPACA9/20250617_MouseSperm_SPACA9magent_MERGED.tif]

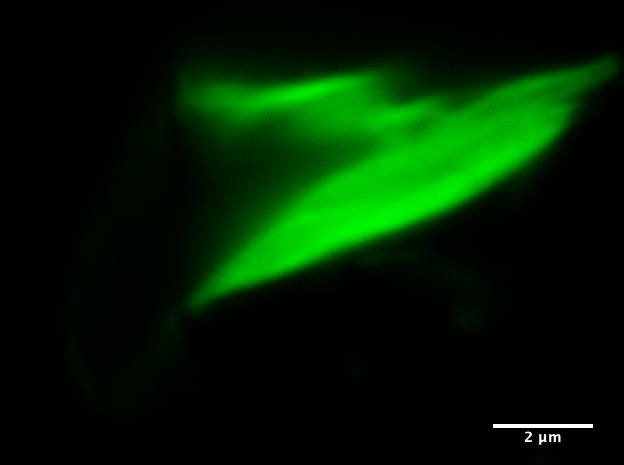

Supplement: Supplementary file 9 — Source data Fig. 3 [file 44318_2026_833_MOESM9_ESM.zip › Fig3/PanelD/SPACA9/20250617_MouseSperm_SPACA9_Alexa 488.tif]

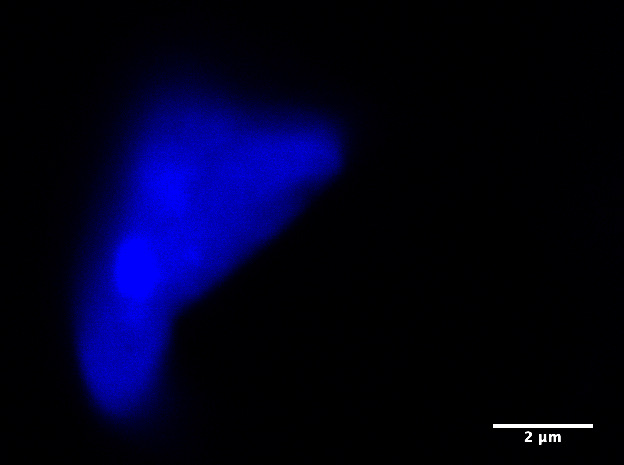

Supplement: Supplementary file 9 — Source data Fig. 3 [file 44318_2026_833_MOESM9_ESM.zip › Fig3/PanelD/SPACA9/20250617_MouseSperm_SPACA9_DAPI.tif]

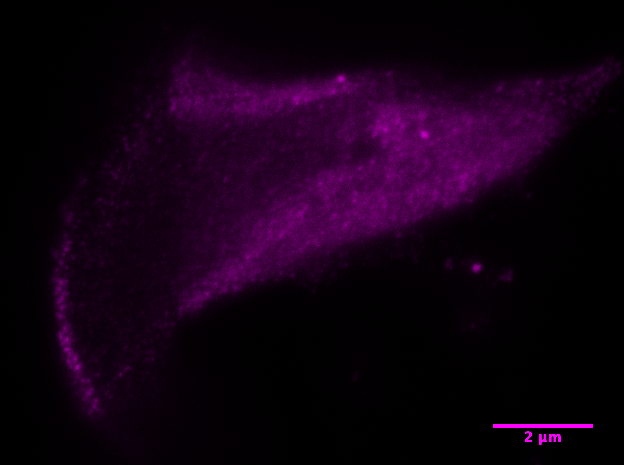

Supplement: Supplementary file 9 — Source data Fig. 3 [file 44318_2026_833_MOESM9_ESM.zip › Fig3/PanelD/SPACA9/20250617_MouseSperm_SPACA9magent_Alexa 594-STED.tif]

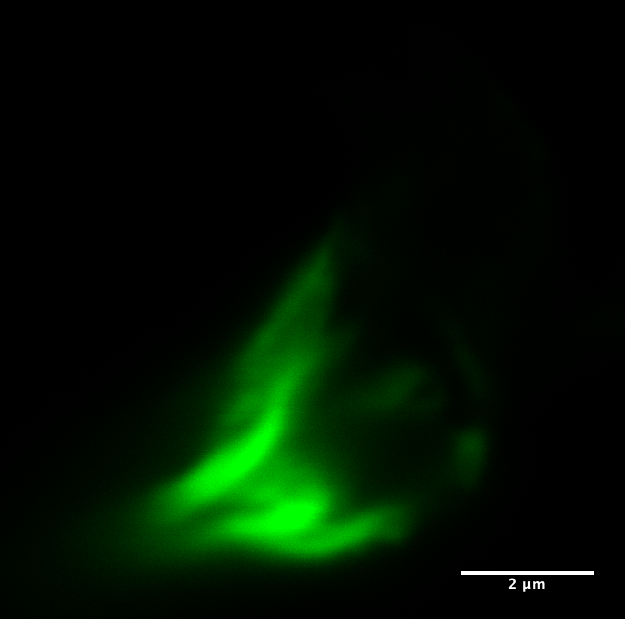

Supplement: Supplementary file 9 — Source data Fig. 3 [file 44318_2026_833_MOESM9_ESM.zip › Fig3/PanelD/SH3D21/20250617_MouseSperm_SH3D21_Alexa488.tif]

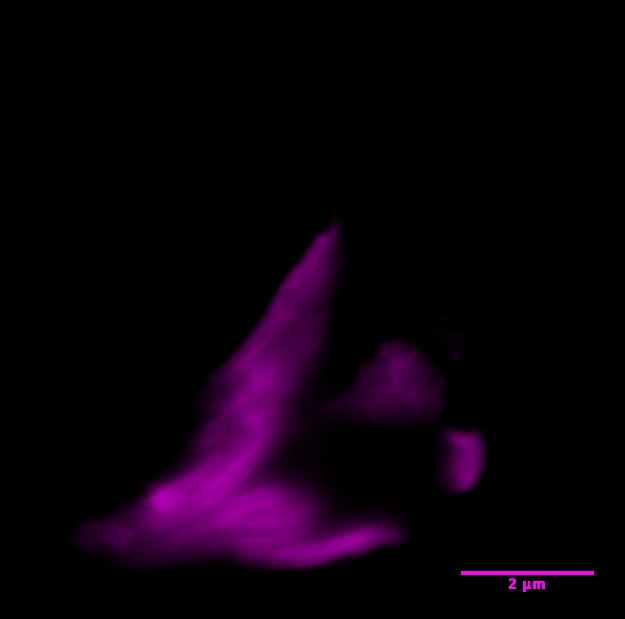

Supplement: Supplementary file 9 — Source data Fig. 3 [file 44318_2026_833_MOESM9_ESM.zip › Fig3/PanelD/SH3D21/20250617_MouseSperm_SH3D21magent_Alexa594_STED.tif]

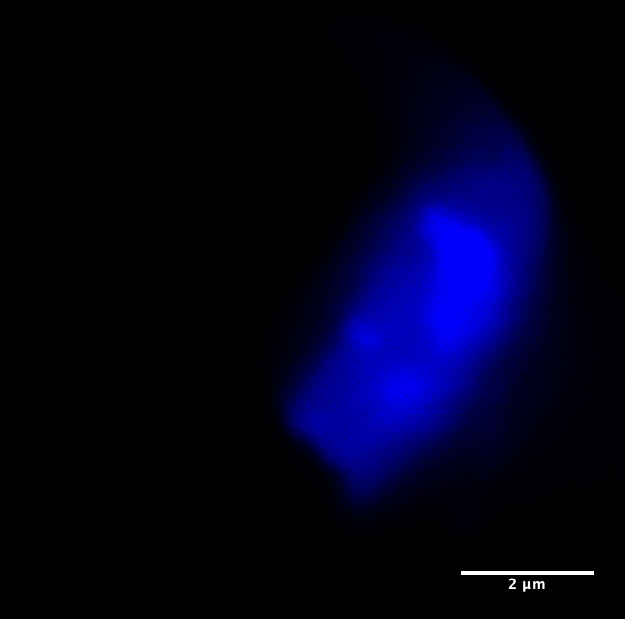

Supplement: Supplementary file 9 — Source data Fig. 3 [file 44318_2026_833_MOESM9_ESM.zip › Fig3/PanelD/SH3D21/20250617_MouseSperm_SH3D21_DAPI.tif]

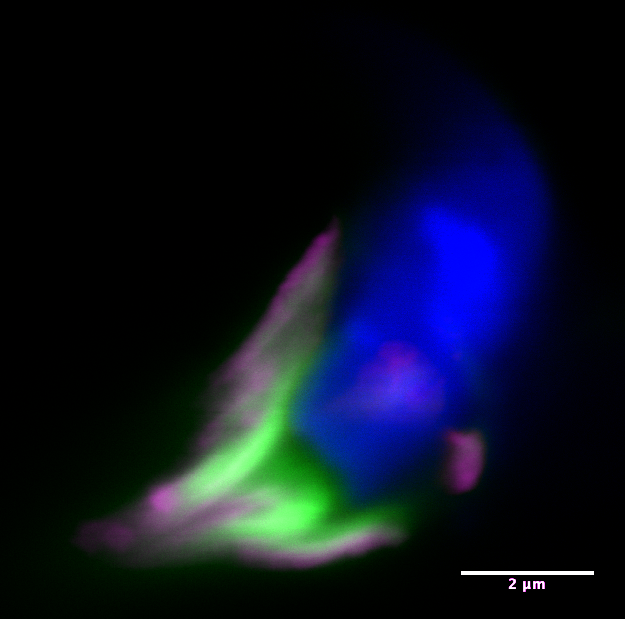

Supplement: Supplementary file 9 — Source data Fig. 3 [file 44318_2026_833_MOESM9_ESM.zip › Fig3/PanelD/SH3D21/20250617_MouseSperm_SH3D21magent_Merged.tif]

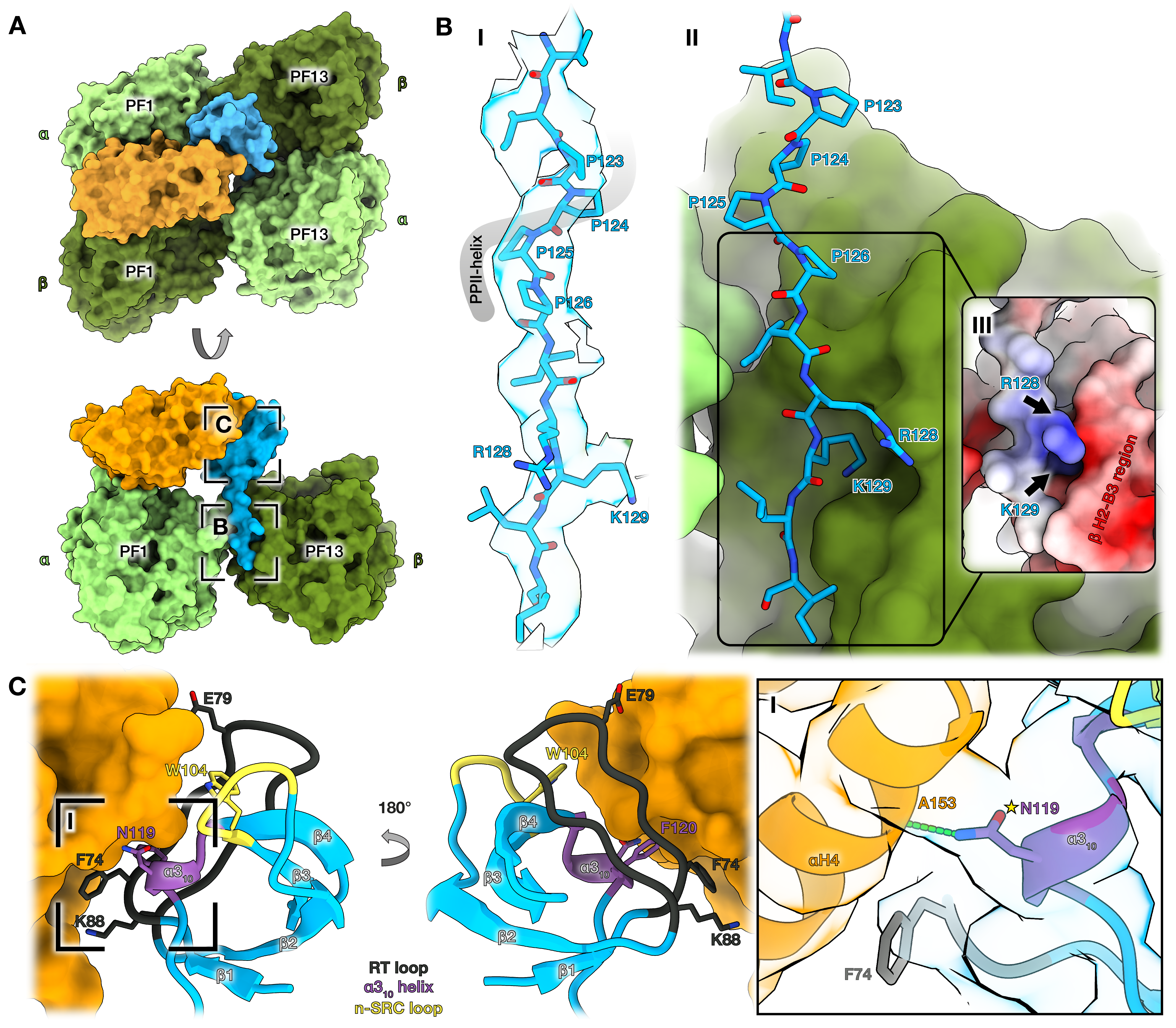

Supplement: Supplementary file 10 — Source data Fig. 4 [file 44318_2026_833_MOESM10_ESM.zip › Fig4/Figure 4.tif]

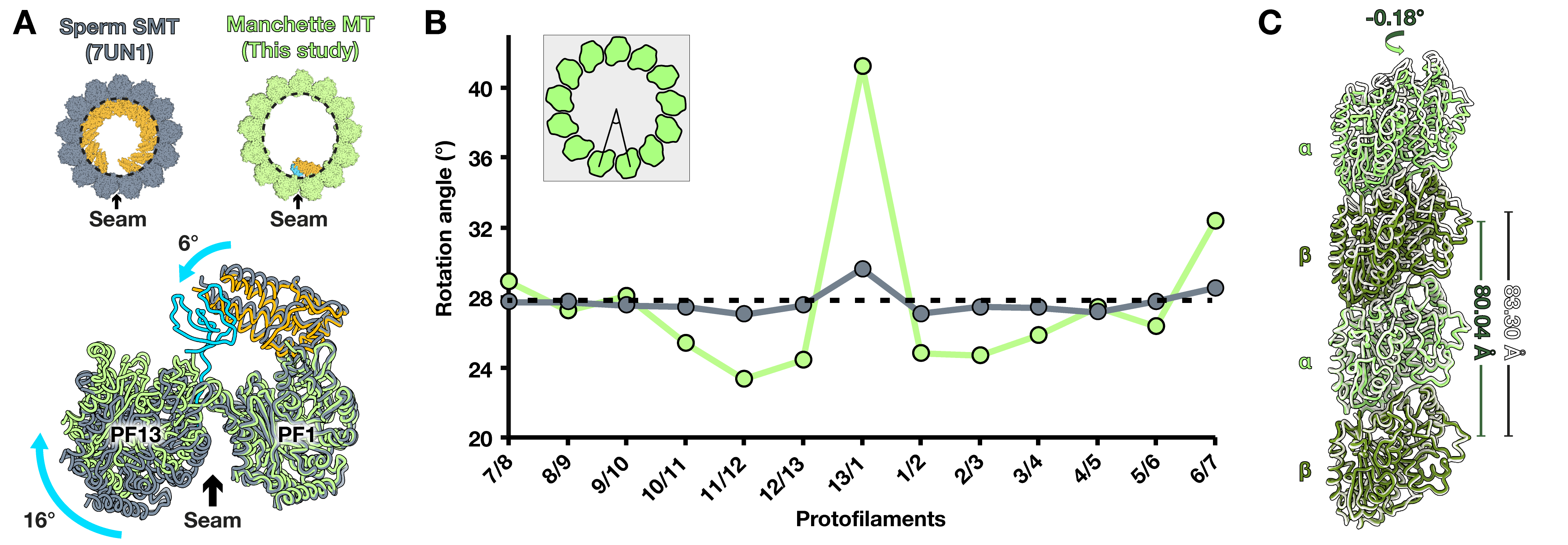

Supplement: Supplementary file 11 — Source data Fig. 5 [file 44318_2026_833_MOESM11_ESM.zip › Fig5/Figure 5.tif]
