# Supplementary material for: SPACA9 and MNMIP1 bridge the seam of spermatid manchette microtubules
Source: EMBO J. 2026 Jun 12;45(14):5024–45. doi: 10.1038/s44318-026-00833-w (PMC13373224; doi:10.1038/s44318-026-00833-w)
Supplement: Supplementary file 12 — Expanded View Figures [file 44318_2026_833_MOESM12_ESM.pdf]

Expanded View Figures

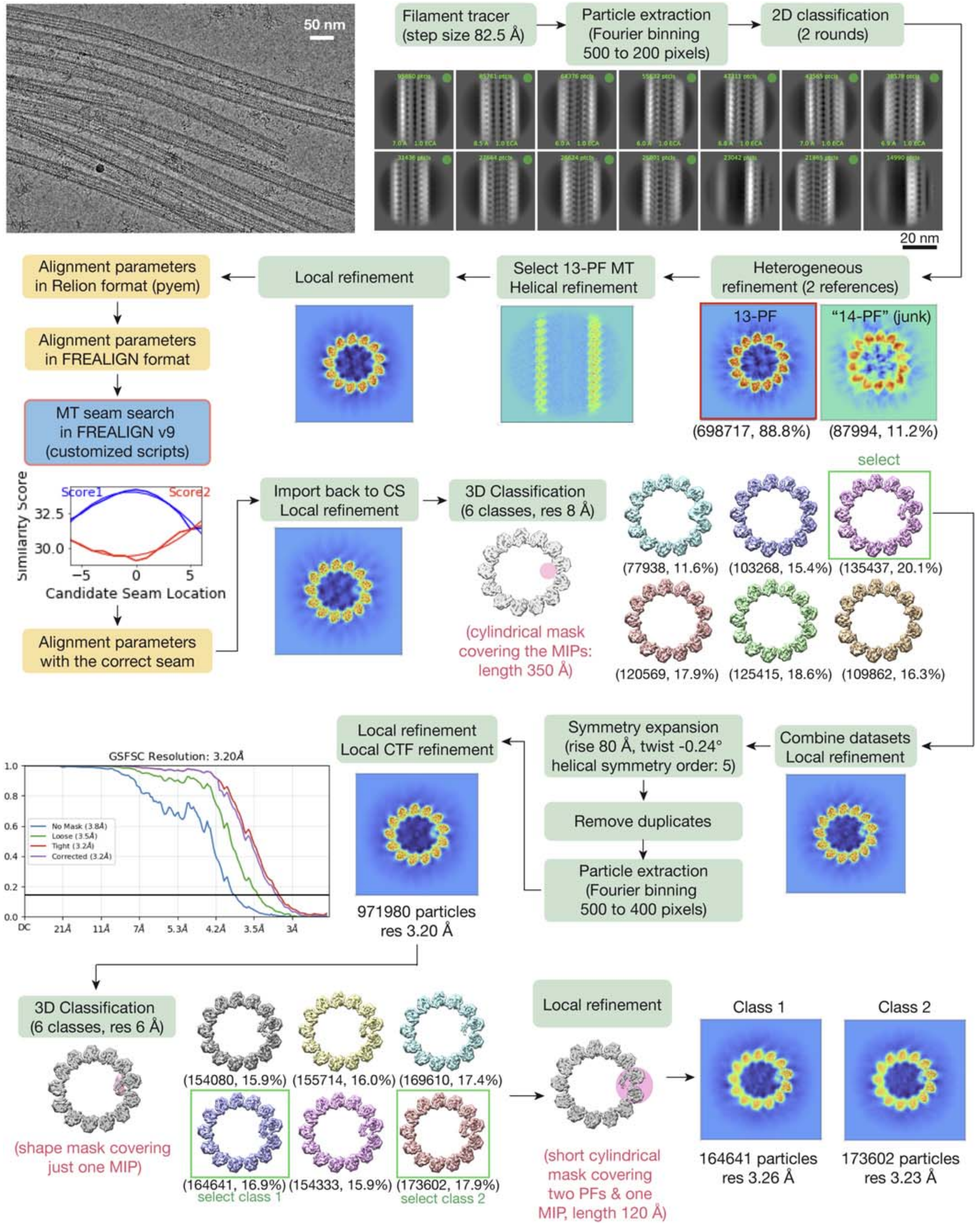

Figure EV1. Cryo-EM single particle analysis workflow of the 8 nm manchette microtubule repeat.

© The Author(s)

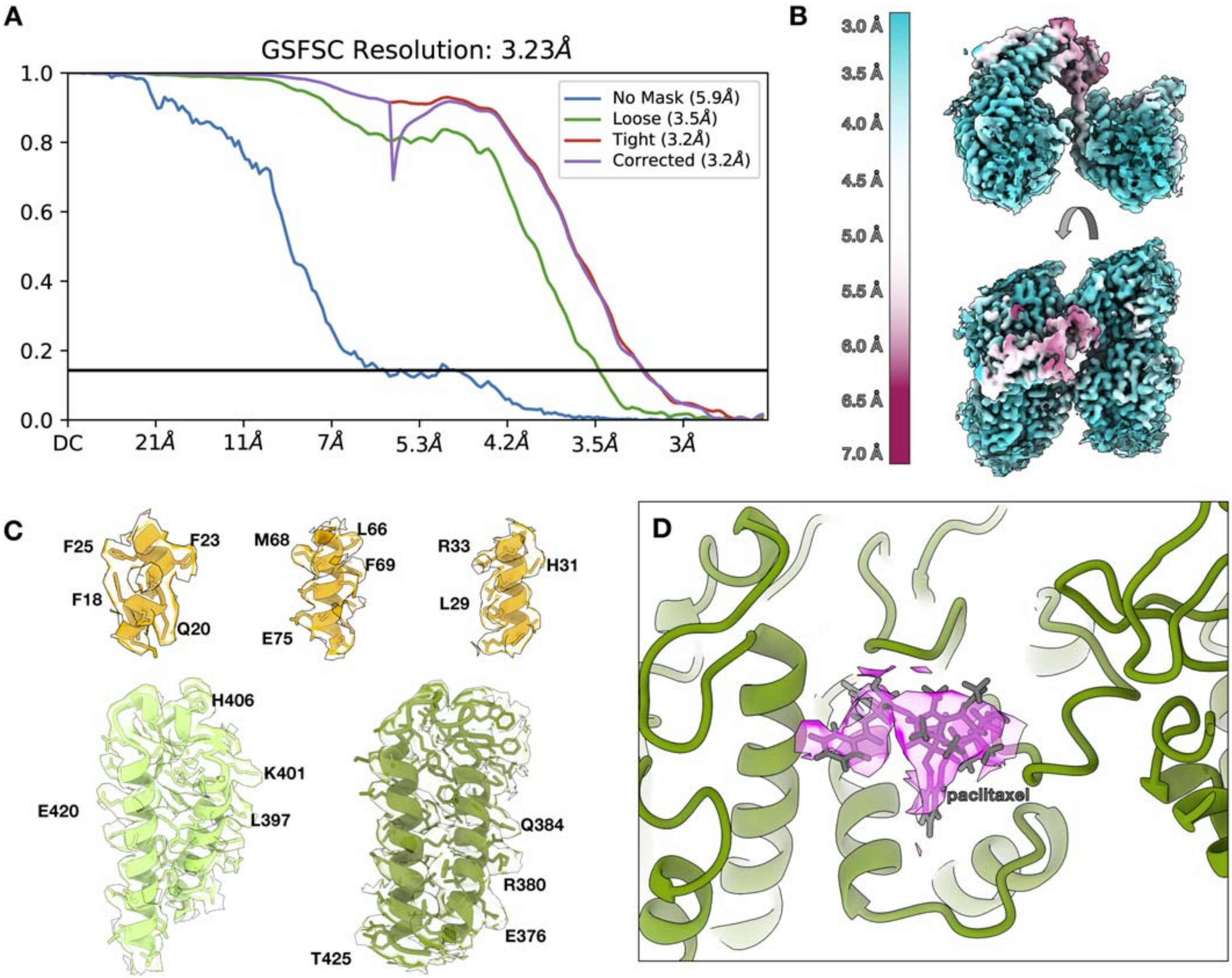

Figure EV2. Resolution estimation of the cryo-EM SPA map.

(**A**) FSC curve of the SPA map of the 8 nm repeat of a manchette MT. (**B**) Local resolution map of the seam region. (**C**) Fitting of the build model of SPACA9 (orange), α-tubulin (light green), and β-tubulin (dark green) into the cryo-EM density map. (**D**) Cryo-EM density for paclitaxel bound to manchette MTs.

 © The Author(s)

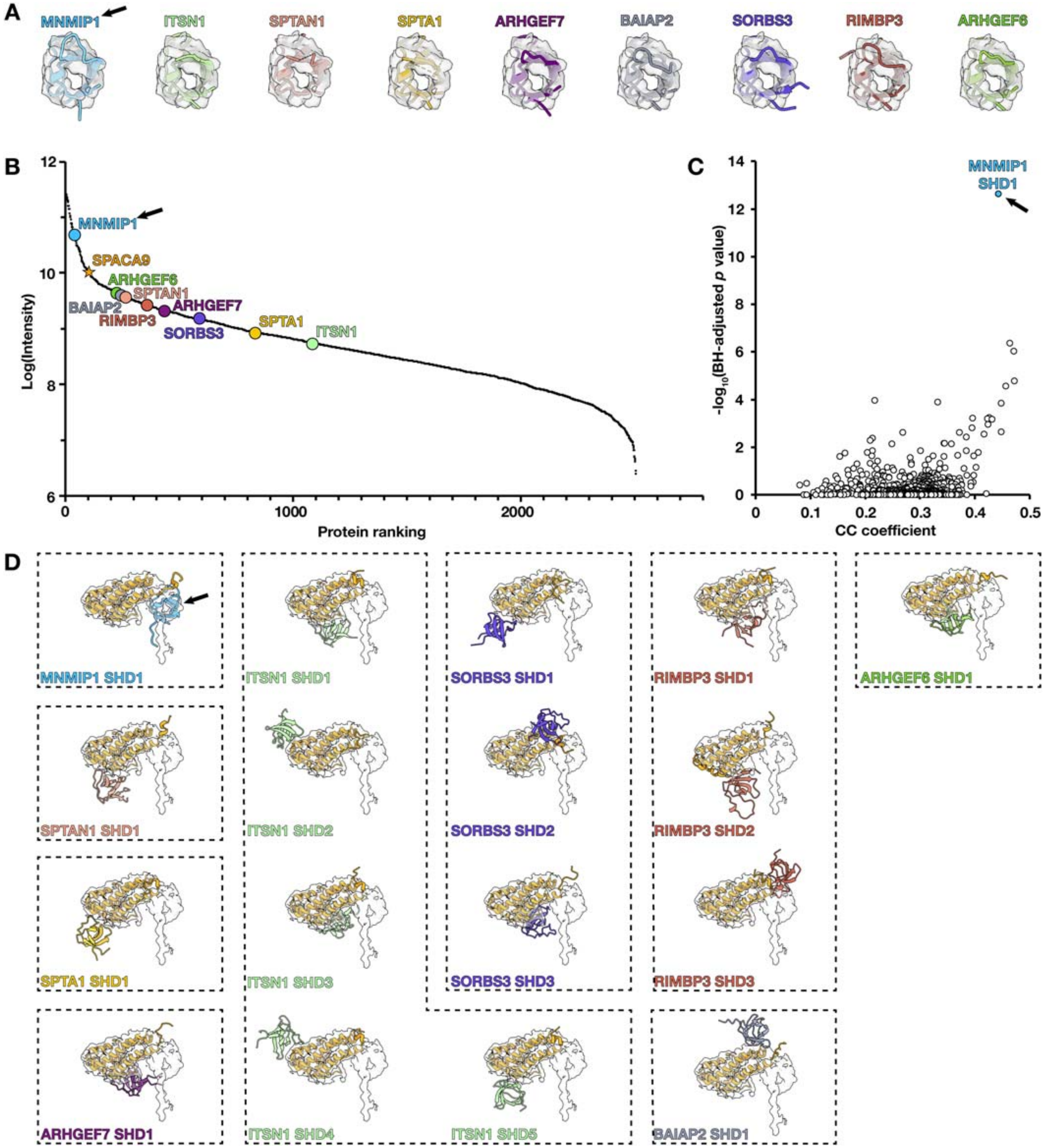

Figure EV3. Identification of MNMIP1.

(**A**) Fit of all SH3 domain proteins in the manchette proteome into the cryo-EM map. (**B**) Relative abundance of SH3 domain-containing proteins in the proteomics of isolated rat manchettes. (**C**) Scatterplot of the DomainSeeker results showing the negative log value of the BH-adjusted p value versus the cross-correlation coefficient of candidates from proteomics data of purified manchettes (Judernatz et al, 2025) into the EM density. The point of MNMIP1 SH domain 1(SHD1) is well separated from the rest of the points cloud, indicating a well-matched fit into the density. (**D**) AlphaFold3 predictions of all SH3 domains in complex with SPACA9 show the SH3 domain of MNMIP1 in the correct position.

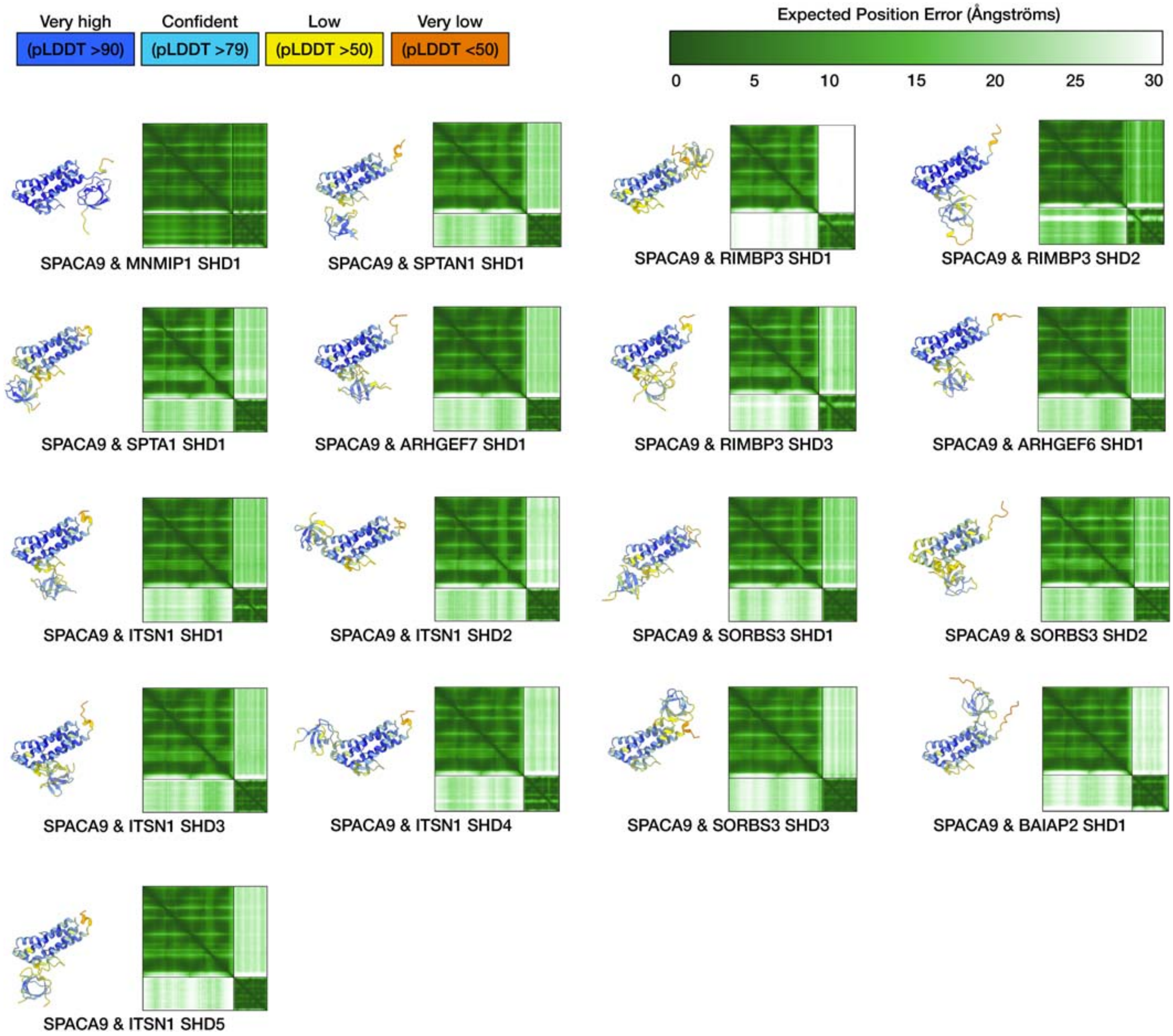

Figure EV4. plDDT scores and PAE plots for AlphaFold3 predictions of SPACA9 and SH3 domains containing proteins in the proteomics data set of purified rat manchettes (Judernatz et al, 2025).

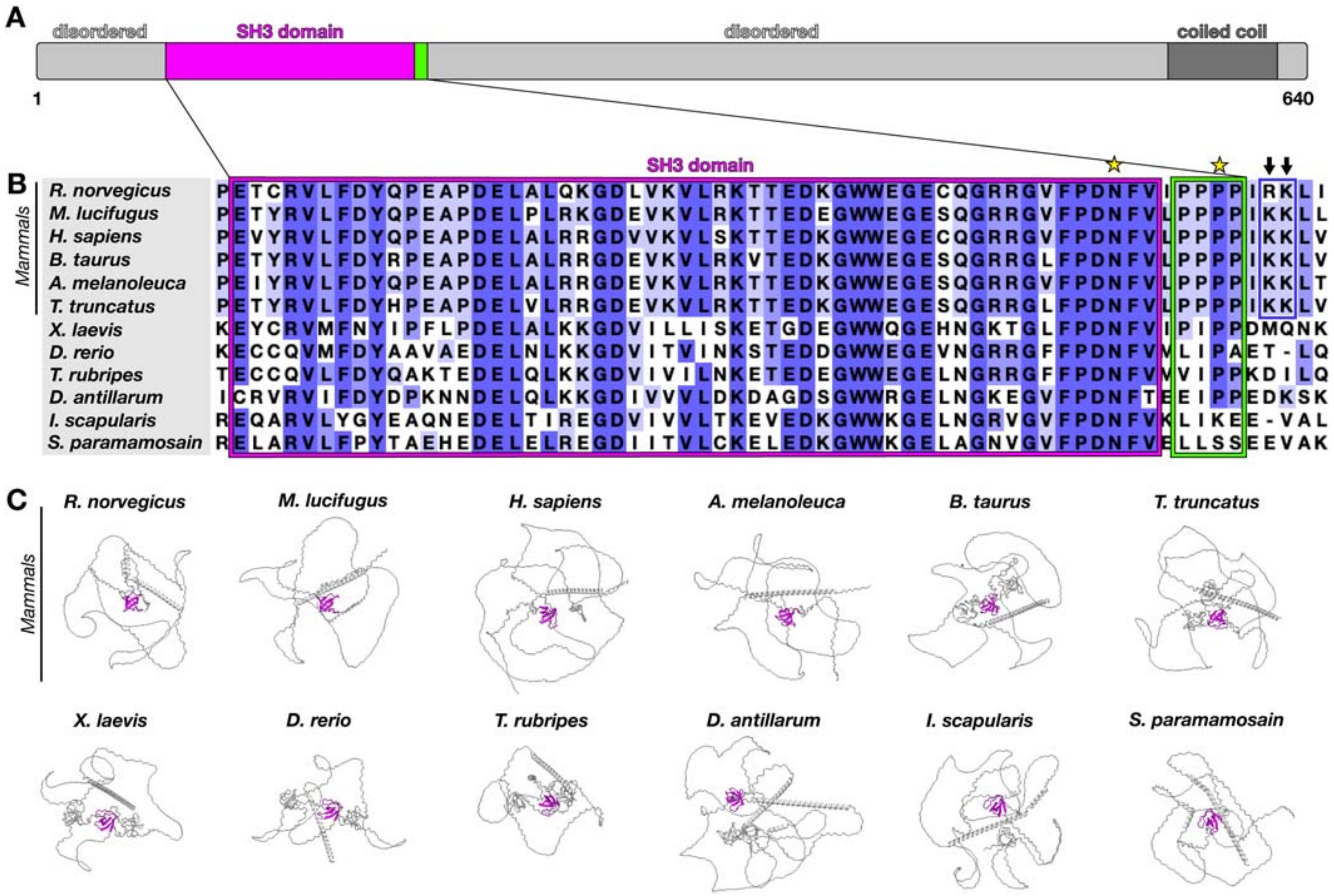

Figure EV5. MNMIP1 SH3 domain is highly conserved.

(**A**) Scheme of domain organization of MNMIP1. (**B**) Sequence alignment of MNMIP1 from several species reveals high conservation of the SH3 domain. Blue color indicates the grade of amino acid conservation. The SH3 domain is marked in a magenta box. The region of the polyproline-II helix is marked in a green box. (**C**) AlphaFold3 models of full-length MNMIP1 from the same species in (**B**) reveal the presence of up to three SH3 domains. Magenta marks the SH3 domain identified via sequence alignment, as shown in (**B**).

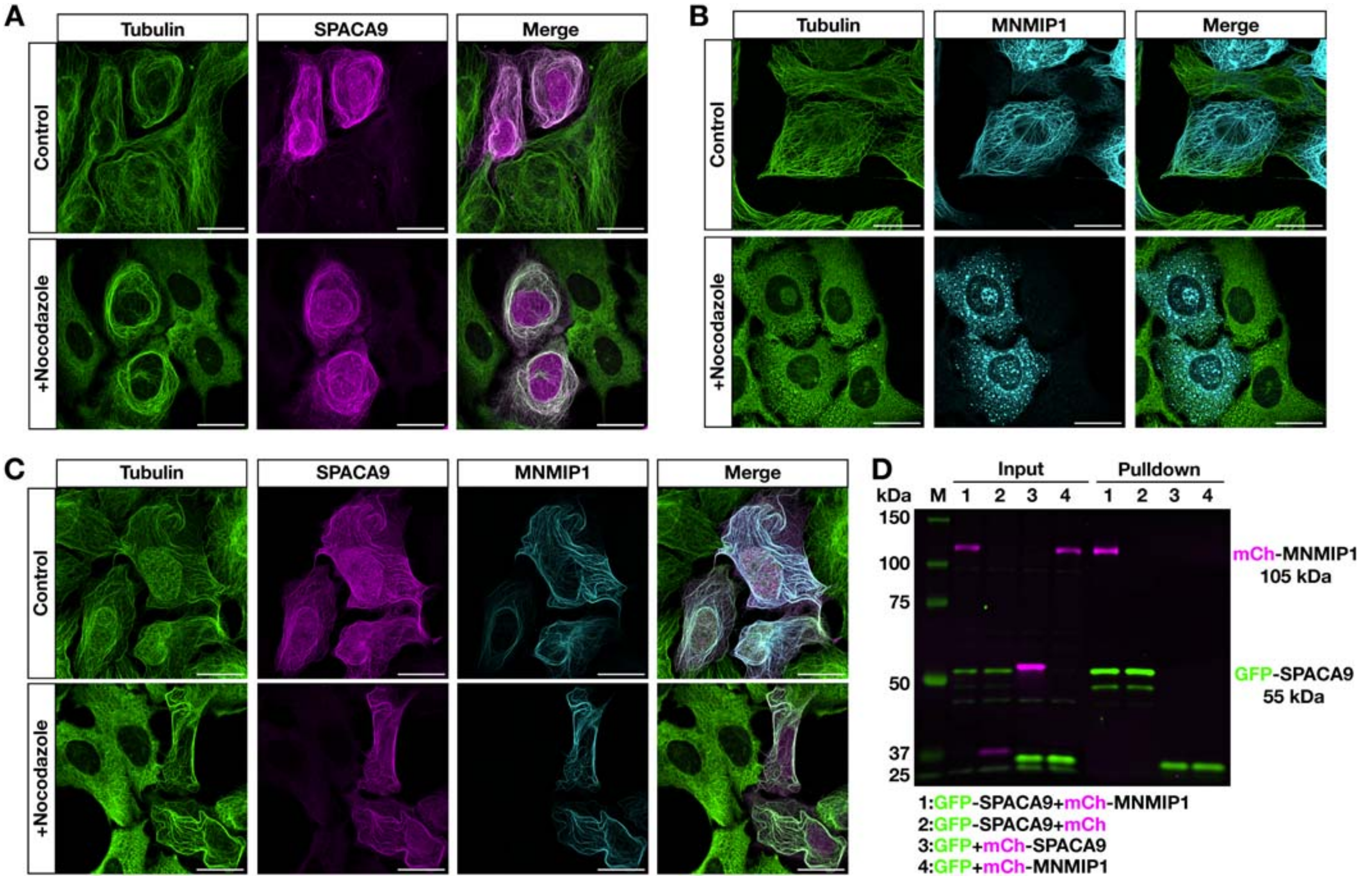

Figure EV6. SPACA9 and MNMIP1 interact in vitro and stabilize MT.

(**A**) Overexpressed mCherry-SPACA9 in U2OS cells colocalizes with microtubules and increases microtubule stability against treatment with 10 µM nocodazole for 15 min. Scale bars, 25 µM. (**B**) Overexpressed EGFP-MNMIP1 in U2OS cells colocalizes with microtubules but does not increase microtubule stability against treatment with 10 µM nocodazole for 15 min. Scale bars, 25 µM. (**C**) Co-expression of mCherry-SPACA9 and EGFP-MNMIP1 in U2OS cells shows colocalization of both proteins on microtubules and increases microtubule stability against treatment with 10 µM nocodazole for 15 min. Scale bars, 25 µM. (**D**) Co-immunoprecipitation of MNMIP1 and SPACA9 confirms the interaction between the proteins in heterologous cells.

 © The Author(s)
