# Supplementary material for: SPACA9 and MNMIP1 bridge the seam of spermatid manchette microtubules
Source: EMBO J. 2026 Jun 12;45(14):5024–45. doi: 10.1038/s44318-026-00833-w (PMC13373224; doi:10.1038/s44318-026-00833-w)
Supplement: Supplementary file 2 — Peer Review File [file 44318_2026_833_MOESM2_ESM.pdf]

THE EMBO JOURNAL

SPACA9 and MNMIP1 bridge the seam of spermatid manchette microtubules

Jo Judernatz, Svetlana Doroshev, Robin Hoogebeen, Sven Jonkers, Donna Schweizer, Molly Gravett, Elizabeth Bromfield, Stuart Howes, Anna Akhmanova, Rui Zhang, and Tzviya Zeev-Ben-Mordehai

Corresponding author: Tzviya Zeev-Ben-Mordehai (z.zeev@uu.nl)

Review Timeline:

Submission Date:	27th Feb 26
Editorial Decision:	22nd Apr 26
Revision Received:	7th May 26
Accepted:	1st Jun 26

Editor: Ieva Gailite

Transaction Report:

Please note that the manuscript was previously reviewed at another journal and the reports were taken into account in the decision making process at The EMBO Journal. As EMBO Press has a transfer agreement with that journal, revision was invited based on the reports from that previous submission.

(Note: With the exception of the correction of typographical or spelling errors that could be a source of ambiguity, letters and reports are not edited. Depending on transfer agreements, referee reports obtained elsewhere may or may not be included in this compilation. Referee reports are anonymous unless the Referee chooses to sign their reports.)

Reviewer #1 (Remarks to the Author):

We thank the reviewer for the constructive comments. Our response and changes to the manuscript are detailed below in blue.

1. The authors propose that SPAC9 and SH3D21 may endow manchette MTs with the unique MT features observed and may be the mechanism for the stability of the manchette, which is uniquely stable for several days during spermatogenesis. However, <u>the authors do not test these ideas</u>, and a couple of simple, feasible experiments come to mind that can address this indirectly without the need for a mouse knockout. For example, the authors could exogenously co-express SPAC9 and SH3D21 in tissue culture cells and test whether MTs are increasingly more resistant to MT depolymerizers. To test a change in lattice spacing upon SPAC9 and SH3D21 expression, the kinesin-1 rigor construct StableMARK can be used to assess whether lattice spacing has changed (see PMID 39387699). If the expression of these proteins does not alter MT structure, then it is likely that additional manchette-binding proteins are required for the unique stability and structure of the manchette.
Following this reviewer's suggestions, we expressed tagged versions of SPACA9 and SH3D21, both separately and together, in U2OS cells. We have used IF staining to check their colocalization with MTs and nocodazole treatment for assaying MT stabilization. We found that SPACA9 and SH3D21 indeed colocalize with MT, and SPACA9 alone and the SPACA9-SH3D21 complex increase microtubule stability. These results are now included in the revised version (lines 202-213 and new supplementary figure S6).

2. SPAC9 usually decorates the MT lumen and not the seam alone. The authors suggest that SH3D21 may confine SPAC9 to the seam. The authors could test this indirectly without additional structural work (or generating a mouse knockout). The authors could exogenously express SPAC9 with and without SH3D21 in tissue culture cells that don't normally express SH3D21 or express it at very low levels, such as HEK293 cells. MTs can be extracted from tissue culture cells (ex: PMID 284344) and then probed for SPAC9 by immunoblot. Presumably, if SH3D21 limits the binding sites of SPAC9, less SPAC9 would be present in the MT extract when co-expressed with SH3D21.
We thank the reviewer for this suggested experiment. However, it is in fact not possible to extract microtubules from tissue culture cells because these are very long cytoskeletal filaments spanning the whole cell. What the paper by Bulinski and Borisy (PMID 284344) mentioned by the reviewer refers to, is extraction of tubulin dimers from cells (the first sentence of the Abstract reads: "Microtubule protein from HeLa cell extracts was purified by multiple cycles of polymerization and depolymerization"). This procedure is based on the fact that mammalian microtubules depolymerize upon chilling to 4℃, and extracted tubulin can be polymerized into microtubules. Indeed, microtubules can be reconstituted from free tubulin in vitro, either in cell extracts or in a system with purified components, but performing such experiments with two previously biochemically uncharacterized proteins is challenging and goes beyond the scope of this study.

3. In the abstract's last sentence, the authors' wording is overstated, in my opinion. The work does not provide the mechanism for MT stability, yet it offers a plausible explanation. To truly demonstrate this, the authors would have to either knock out these components and show that manchette stability disappears (beyond the scope of this work) or attempt to demonstrate that SPAC9 and SH3D21 endow MTs with increased stability using tissue culture cells or an in vitro system. Given the number of MAPs associated with the manchette, SPAC9 and SH3D21 may not be solely responsible for manchette stability.
We have followed the reviewer's suggestion and have performed the experiment in tissue culture cells with nocodazole treatment (new Fig S6 also mentioned in Point 1). The nocodazole experiments show that SPAC9 and SH3D21 increase MT stability. Nevertheless, we agree with the reviewer that there could be other factors that are needed for manchette stability and thus we toned down the last sentence of the abstract. It now reads: 'Our study reveals a novel seam microtubule inner protein complex with a unique binding mode that could provide one plausible explanation for MT regulation that maintains manchette integrity over an extended period, crucial for sperm development.'

4. It's unclear why the authors opted for STED in some cases, as for SPAC9, but not in other cases, such as for CLASP2. Please clarify.
For consistency, we have now imaged all the manchette IF stainings with STED microscopy (Revised Fig. 2).

5. Line 160: Please state what the positively charged residues are.
This information was added: (R128 and K129 of SH3D21) line 175.

6. Line 179: Please define "SAXO" protein.

SAXO was defined in the revised version: stabilizer of axonemal microtubules line 187.

7. Line 236: Another reference that can be included here is a recent preprint: https://www.biorxiv.org/content/10.1101/2025.03.12.642813v2.
We thank the reviewer for suggesting this additional reference. We have added it to the sentence.

8. The last sentence in the discussion, lines 242-243, is unclear as to what the authors are suggesting. Are the authors assuming that these proteins are removed for the disassembly of the manchette to take place? That's a possibility, but not necessarily true. This sentence implies quite a bit that the authors could expand upon or reword.
We agree that our data does not provide information regarding the disassembly of the manchette. Accordingly, we have reworded our statement and removed the term "removal" of proteins. The last sentence now reads: 'While SPACA9 is a well-established MT-binding protein in axonemal MTs, we present SH3D21 as a previously unidentified MT-binding protein that, when in complex with SPACA9, can bind to the seam of manchette MTs, marking the first observation of a seam-binding protein complex outside axonemal MTs.' Line 262-265.

Reviewer #2 (Remarks to the Author):

In this manuscript by Judernatz et al., the authors present a structural and immunofluorescence analysis of some of the proteins found in manchette microtubules in developing rat spermatids. This work follows a recently published study by the authors on the ultrastructure of manchette microtubule arrays (DOI: 10.26508/lsa.202503415). Here, the authors first use cryo-ET to confirm that microtubules in isolated manchettes contain mainly 13 protofilaments and have a polarity that places nearly all of their plus ends towards the perinuclear ring. From their tomograms, they also show that microtubule lattices in both plus and minus ends exhibit a range of uncapped end-structures, and observe that CLASP2 broadly colocalizes with microtubules throughout the manchette. Additionally, they observe a variety of decorating proteins both outside and inside the manchette MTs, and focus on identifying a specific repeating array of inner proteins. Using single particle cryo-EM, they generate a 3.2 A reconstruction of manchette microtubules and show that SPACA9, a MIP present in axonemal doublets and singlet microtubules at the ends of sperm flagella, interacts with SH3D21 and tubulin only at the seam and in a manner that allows SH3D21 to thread between protofilaments 1 and 13 towards the outer face of these specialized microtubules, where supposedly the rest of SH3D21 performs its functon(s).

The manuscript is clearly presented and relatively well-written, and the study addresses some long-standing questions about the molecular nature of microtubules in the manchette, a specialized microtubule array transiently appearing during spermiogenesis and required for correct formation of mature sperm. As outlined below, I have a few concerns regarding the conceptual advances offered by this study. Specifically, the authors make several claims about mechanisms that in my opinion are not supported by convincing evidence, and would need addressing prior to publication.

We would like to thank the reviewer for their detailed assessment of our manuscript and all the suggestions that greatly improved our manuscript.

Major points:

1. The observation that manchette microtubules contain 13 protofilaments and have their plus ends oriented towards the PNR is not necessarily novel, given that the authors recently showed 13 pf cross-sections in figure s4 of their recent LSA paper, as well as since MAP localization (as cited in the manuscript) and early EM studies (https://doi.org/10.1016/1047-8477(92)90034-8) have already defined the polarity of MTs in the manchette. The authors should tone-down the reported impact of these observations.

We agree that the polarity and PF number are not novel observations; we added the quantification of the PF number and polarity for a more general audience. We have removed the novelty claim: the section title is now 'Manchette MT ends are structurally variable' and we state that this was confirming previous results: 'To confirm the MT polarity and PF number in our isolated manchette preps,'.

2. The authors provide beautiful cryo-ET data of higher quality and resolution than in their recent LSA study, yet little analysis or attempt to characterize the other decorating proteins is made. For example, in the LSA study, the authors were able to identify dynein-dynactin assemblies. Several

MIPs and MAPs shown in Fig. 3a seem potentially averagable. Further, the relative location of MIP/MAP-decorated MTs in the manchette might suggest something about the role of e.g. SPACA9-SH3 in this large structure. Do all manchette MTs contain SPACA9-SH3? Further analysis would be warranted for this study, and the data are on hand.
The densities highlighted with yellow triangles in Fig 3a are, unfortunately, not sufficiently regular for high-resolution structural analysis.

Regarding the relative localization of SPACA9-SH3D21 along the manchette, our IF in spermatids demonstrates that the proteins are present all along the manchette (Fig. 3d). In our cryo-EM SPA data analysis, we find about 20% of manchette MTs clearly decorated with the complex (we have added this information to a processing supplementary figure (new Fig. S1)). But it should be noted that the quantification from the SPA should be taken with caution because it was done on isolated manchettes, and we cannot exclude the possibility that some complexes fall off during the isolation (there is no fixation during isolation). As such, the IF imaging is a better technique for the localization of SPACA9-SH3D21 along the manchette, as it was done in spermatids.

3. Line 147: The authors need to show the outcomes of all of these AlphaFold predictions, including PAE plots and the structures coloured according to pLDTT scores. Otherwise it is difficult for the reader to assess why one model would be more likely than others.
We thank the reviewer for this suggestion. We have added PAE plots and pLDTT scores for all predictions in a new, separate supplementary figure (new Fig. S4). Of note, both PAE plots and pLDTT scores are clear for the prediction of the SH3D21-SPACA9 complex.

4. The authors should explain why super-resolution microscopy was necessary for Fig. 3d when normal IF was sufficient for Fig 2. The authors should also comment on why their IF, even with super-res methods, is unable to resolve or outline individual MTs in the manchette structures?
As also requested by the other reviewers, we have now imaged all the IF stainings of the manchette with STED microscopy (Revised Fig. 2). The manchette MTs are highly dense, making it harder to resolve individual ones, however, bundles are sometimes resolved, see for example in Fig. 2 and the added movies.

5. In Figure 2. From line 120 - 132, the authors seem to suggest that CLASP2 should localize to both ends of microtubules, and reference 50 also seems to suggest that CLASP2 binds specifically to the ends of microtubules. Can the authors address why CLASP2 is present in the whole manchette in Fig.2c, rather than at the ends of microtubules?
Many MT end-binders can also bind along MT lattice, an effect more obvious at higher protein concentrations, and this is well-established for CLASPs (refs 48-50). CLASP2 is likely present along the whole manchette for two reasons: first, because its concentration in spermatids is high, and second, because whilst most MTs plus ends are at the perinuclear ring, the minus ends are at different points along the manchette. We have added IF staining for a minus-end marker, CAMSAP1, which, in agreement with our cryo-ET data, shows that the minus ends are present within the manchette (see revised Fig 2). As CLASP2 binds to both plus and minus ends, and can associate with MT lattice, albeit more weakly, the signal is across the entire manchette.

6. In Figure 2 and Figure 3. The localizations of CLASP2-CLIP170 and SPACA9-SH3D21 were done separately. It would be more convincing if CLASP2 and CLIP170 were shown in the same sample (i.e.

colocalization). Same for SPACA9-SH3D21.
We would have liked to do the colocalization together but antibody availability limited our options. The antibodies we used were rabbit anti-CLIP170 and anti-CAMSAP1, and rat anti-CLASP2. However, the only secondary antibodies suitable for STED available to us were Alexa Fluor 594 for both anti-rabbit and anti-rat secondaries, precluding spectral separation and resulting in unreliable co-localization. Primary conjugated antibodies were not available and are less suitable for STED because they do not allow signal amplification by secondary antibodies. Therefore, each protein was imaged separately. Importantly, all proteins co-localized with the manchette (α-tubulin), supporting their spatial distribution.

7. Regarding the interaction between SPACA9 and SH3D21, the authors could include further validation. For example, this interaction could be validated biochemically using recombinant SPACA9 and the SH3 domain of SH3D21. The authors even suggest a conserved residue N119 that would be a prime target to mutate and disrupt such a potential interaction.
We have followed the reviewer's suggestions for further validation. We have expressed both SPACA9 and SH3D21 separately and together in U2OS cells. IF showed colocalization with MTs (see new Supplementary Fig 6). We further performed pull-down assays showing that the proteins interact in extracts of overexpressing HEK293 cells (see Fig. S6d).

8. An illustrative workflow showing how the SPA reconstruction was obtained is missing and would be helpful. Related to this, I wonder why less than 20% of MT particles contain strong MIP density after classification. Does this suggest that the SPACA9 decoration only occurs on a subset of MTs in the manchette?
We have followed the reviewer suggestion, and a figure summarizing the SPA workflow is now provided (new supplementary Figure S1). Indeed, only 20% of microtubules show a clear decoration with the protein complex. As mentioned above in point 2, this might be due to dissociation of the complex from MTs during manchette isolation. The IF and mass spec, however, demonstrate that the complex is highly abundant on spermatid manchettes.

9. There is a reference to using "quantitative analysis" for assigning SH3D21 via docking, but the results of this analysis are not shown in the paper. Please include.
We apologize for the omission; the results of this analysis are now provided in the revised Fig S3c.

Regarding mechanistic insights in general. The authors make several claims that are unsubstantiated. These include:

10. The suggestion that SPACA9-SH3D21 confer mechanical stability to manchette microtubules, even in part. The authors use taxol, a stabilizing small molecule, to isolate the manchettes. It is not clear why taxol is necessary, especially if MIPs like SPACA9-SH3D21 and MAPs like CLASP2 work together to stabilize these structures. Do manchette MTs disassemble if the prep is performed without taxol? This contradiction needs to be addressed.
The addition of Taxol is indeed a good point. We have performed the manchette isolation both with and without Taxol. In both cases, we were able to get the manchettes. However, in the absence of Taxol, MTs were structurally less well preserved, thus less suitable for high-resolution

structural analysis. For that reason, we have decided to continue using Taxol. Our interpretation is that, manchette stability is likely the collective property of multiple MAPs and MIPs and our data provide two of the mechanisms and potentially there are more. To reflect this point in the manuscript, we have rephrased the end of the abstract to read: 'Our study reveals a novel seam microtubule inner protein complex with a unique binding mode that could provide one plausible explanation for MT regulation that maintains manchette integrity over an extended period, crucial for sperm development.'

11.The reports that SPACA9 and SH3D21 depletion in mice has no effect on fertility is striking given the authors' model that the proteins work together as a molecular staple to hold together the weakest part of the MT lattice, the seam. What does the manchette structure look like when one of these proteins is knocked out? Can alphafold be used to predict (including tubulin) whether similar staple structures form with homologs from human vs. mouse vs. rat? If not, can biochemical reconstitutions of MTs with purified MIPs be done to test effects on MT stability, especially when combined with CLASP2?

Unfortunately, in the original publications of the single SPACA9 and SH3D21 knock-outs, spermatids were not imaged, so there is no information on the manchette in these KOs. Given our discovery of the SPACA9/SH3D21 complex in the manchette, a double knock-out would be very interesting, but this goes beyond the scope of the current study.

To show that SPACA9 and SH3D21 increase MTs stability, we added nocodazole treatment experiments in tissue culture cells overexpressing SPACA9 and SH3D21 (see new Supplementary Figure 6). Biochemical in vitro reconstitutions combining SPACA9, SH3D21 and CLASP2 could be performed but would be very challenging, because with three proteins, a large parameter space of protein concentrations would need to be explored. Such experiments go beyond the scope of the current study.

Regarding the suggestion to use AlphaFold predictions, AlphaFold still struggles to predict intact microtubules and only associates tubulin dimers longitudinally into a single protofilament, but not laterally, which would be necessary to create the binding site for the complex.

12. The authors claim in the discussion (line 203) that their results suggest CLASP2 is a crucial component of the manchette that likely acts by stabilizing MTs at their plus and minus ends. But the data just show broad colocalization with MTs. This contradiction needs to be addressed.

As explained in point 5, manchette MTs have different lengths; in particular, minus ends can be found at different points along the manchette, as shown by CAMSAP1 staining (revised Fig 2). As such, when staining with CLASP2, a broad localization is observed, also because CLASP2 can bind along MT lattice.

Minor points:

1. Line 45: This sentence is difficult to read. Do the authors mean "At the seam, lateral interactions between protofilaments (PFs) are heterotypic (α-tubulin interacts with β-tubulin) rather than homotypic (α–α or β–β interactions), as seen throughout the remainder of the MT lattice"?

We thank the reviewer for pointing this out and have revised the sentence exactly as suggested. Line 46.

2. Line 64 and throughout the manuscript: the authors repeatedly stress that manchette MTs are the first evidence of cytoplasmic MTs having new MIPs, especially seam-specific ones. But can it really be said that these are akin to cytoplasmic MTs? It could be counter-argued that their apparent high degree of stability, association with IFT proteins, and colocalization with centriole biogenesis components like delta and epsilon tubulin makes them rather unlike conventional "cytoplasmic" microtubules.
We agree with the reviewer that manchette MTs are not like cytoplasmic MTs. We have removed the claim on cytoplasmic microtubules throughout the manuscript and instead refer to non-axonemal MTs.

3. Line 115: Confusing wording. Something like "100% (102/102) of MTs had their plus-ends oriented towards the perinuclear ring (Fig. 1e)" might be better.
We have reworded this sentence. It now reads: At the perinuclear ring side, 100% (102/102) of MTs had their plus-end (Fig. 1f). Line 126.

4. Line 128: Confusing wording. CLASP2 has already been shown to "play a role" in manchette MTs. Can the authors be more specific as to why they are looking at CLASP2 here?
The microtubule plus and minus ends structures we observed in the manchette resemble CLASP2-stabilized ends. The only available reference for the role of CLASP2 in the manchette has been shown in a publicly available doctoral thesis (Drabek 2005), but the results were never published in a peer-reviewed journal, nor on a preprint platform.

5. Line 141-142: Why do the authors need to use a docking-based approach, when ModelAngelo worked for the adjacent SPACA9 subunit?
ModelAngelo works well for resolutions better than 4Å. The region of SPACA9 that interacts with the microtubule was resolved to ~3 Å, thus ModelAngelo worked well for SPACA9. The region of the map corresponding to SH3D21 was in the range of 4-6 Å thus ModelAngelo could not be used for that region. In the resolution range of ~4-7Å domain-based docking approaches are used.

We have added this information to the text. Lines 152-156.

6. Line 158: I don't understand what it means to be structurally supported by a conserved stretch of prolines. Can the authors clarify?
We have reworded this sentence. It now reads: 'The region of amino acids 122-131 of SH3D21 is characterized by a conserved repeat of four prolines forming a left-handed polyproline (PP) II helix (Fig. 4b and Fig. S5). The PPII helix orients the subsequent amino acids perpendicular to the MT lattice…'. Lines: 172-175.

7. Line 160: positions 128 and 129 of what?
Here, we refer to the residues of SH3D21, as shown in the figures. We have clarified this in the text. It now reads: 'which allows two conserved positively charged amino acids (R128 and K129 of SH3D21) to interact with the negatively charged residues in the H2-B3 region of β-tubulin (Fig.

4b).' Line 175.

8. Line 165: alpha chain of A153. Do the authors mean the alpha carbon? Please clarify.
We thank the reviewer for pointing out this typo. We corrected it to alpha carbon. Line 181.

9. Line 176: "SH3D21 or SPACA9 and SH3D21 compact…" shouldn't this read "SH3D21 or SPACA9 and SH3D21 to compact…" ? In general this sentence is difficult to follow.
We appreciate that this sentence was not clear. It was revised together with the rest of this paragraph. Lines 195-198.

10. Line 212: this sentence needs to be checked for grammar.
We rephrased this sentence. It now reads: 'Both SPACA9 and SH3D21 are highly enriched during spermiogenesis and have been studied in the context of sperm formation. Yet, a single knockout of either SPACA9 or SH3D21 does not seem to affect fertility in mice[66,67]. Line 230-232.

11. Fig.1c and Fig.1d do not clearly show which part of the sperm that manchette corresponds to. It would be better to include an illustration of the sperm intermediate that contains manchettes to help understand the location of manchette in sperm.
We added a small illustration of an elongating spermatid to Figure 1a to clarify the position and role of the manchette during spermiogenesis in spermatids.

12. The authors could include the rotation direction of the microtubule in Fig.1e and 1f, which would help the discussion of microtubule orientation. This could either be an arrow or plus/minus sign.
We added arrows indicating the slew of each MT average (revised Fig. 1f & 1g).

13. The color of IF images should be reconsidered, it would be better to avoid using green and red together for colour-blind folks.
We have replaced the red with magenta as advised.

14. In Supplementary Figure 2. What if the authors include tubulin dimers in AF predictions? Would there be more SH3 domains that could fit into the density after including tubulin dimers in AF predictions?
We considered this and have tried that before, but AF is not able to predict laterally interacting tubulin dimers.

15. Regarding the elliptical nature of the microtubules in the SPA reconstruction - can the authors speculate what causes this? The 40 deg rotation of tubulin at the seam is striking. Are there changes in tubulin-tubulin contacts that accommodate this rotation?
We could not observe clear differences in tubulin contacts.

16. The reported resolution in the PDB pre-validation report is 3.50 Å, while the manuscript claims 3.2 Å. Can the authors clarify this discrepancy?
This discrepancy is due to masking. We added to the FSC plot (revised Fig. S2a) an FSC with a loose mask (which gives 3.5Å) and with a tight mask, the resolution estimation is 3.2Å.

17. Taxol is a commercial trademark for the compound paclitaxel; it would be more appropriate to use the scientific term for the compound throughout the manuscript.
We have changed the name of Taxol to paclitaxel throughout the manuscript.

18. The pHs of buffers used in the methods are not listed. Please specify.
We apologize for the omission, we have added this information to the revised version.

19. Line 338: Unclear wording "followed by two additional three washes", please clarify.
We apologize for this typo. We corrected it, and it now reads: 'followed by three washes with 400 µL of DPBS.'. line 340.

Reviewer #3 (Remarks to the Author):

In this work, Judernatz et al. investigate the organization and structure of manchette microtubules isolated from rat spermatids. They find that these microtubules are uniformly aligned with the plus ends localized at the perinuclear ring. The plus and minus ends are structurally heterogenous and the microtubule seam is bound by two proteins: SPACA9, which has previously been found associated with various axonemal structures, and SH3D21, which they propose as a novel seam-binding protein and regulator of microtubule stability.

The structural characterization of manchette microtubules is a valuable contribution to our understanding of how specialized microtubule-based cellular structures are generated and stabilized. However, several claims are not fully supported by the presented data. Some concerns could be addressed by including additional data of existing analysis and clarifying the methods. Other conclusions – particularly regarding CLASP2's role – require further experimental support.

The structure raises interesting questions about whether SPACA9 can form other multi-protein complexes in microtubules, and how SH3D21 manages to thread through the lattice. The manchette shows clear morphological changes during spermatid differentiation and the role of SH3D21 in this process would be of interest for future work. The expression and abundance of SH3D21 in different tissues is not fully discussed and so whether this could be a wide-spread mechanism for microtubule regulation is an open question.

We thank this reviewer for the valuable suggestions to improve our manuscript.

Major points
1 - The claim that CLASP2 binds both ends of manchette microtubules is not convincingly supported. The immunofluorescence in Fig 2C shows CLASP2 is distributed throughout the manchette, which could suggest lattice-wide localization rather than end-specific binding. If manchette microtubules

vary in length, minus ends may be dispersed, potentially explaining this pattern. While CLIP170 is used as a plus-end marker, a minus-end marker such as CAMSAP1 should be included to validate localization to both ends. Furthermore, the interpretation of microtubule end morphology as indicative of CLASP2 binding (line 126) is problematic. Splayed ends can occur independently of CLASP2, as shown in prior studies (e.g., PMID: 7775577). Given that microtubule ends are very sensitive and this study is carried out on purified, taxol-stabilised manchettes, I have additional concerns that reliable conclusions can be made about the state and morphology of microtubule ends when these could be altered during purification.
We thank the reviewer for the suggestions to include CAMSAP1 staining. It is now provided in our revised version (revised Fig 2c). The CAMSAP1 staining indeed shows that manchette MTs have different lengths, as also observed by cryo-ET. While most MTs plus-ends are at the perinuclear ring, the minus-ends are at different points along the manchette. As the staining was done on spermatids, it shows that the length variability is not due to the manchette isolation. Likewise, the staining of CLASP2 was done in spermatids and not on isolated manchette. However, we fully agree that CLASP2 could be binding not just to MT ends but also along MT lattice, and modified the text accordingly. Lines 141.

2 - The conclusion of exclusively 13 protofilament microtubules being present in the manchette by cryo-electron tomography (line 114) is contradicted by single particle processing workflow, when 13% are found to match a 14 protofilament microtubule reference (line 401). Since this classification was performed after discarding junk particles, the detection of 14 protofilament microtubules may reflect a real biological feature. If the authors think this is due to poorly aligned particles or technical reasons, images of the maps should be provided.
Particles that were removed during protofilament sorting, which are not 13 protofilament microtubules, are indeed misaligned particles, and no clear 12 or 14 protofilament class was identifiable. This confirms our observation in cryo-ET that most microtubules have 13 protofilaments. We now include in the revised version a workflow of the data processing showing the results of all the steps (new Fig. S1).

3 - The claim that manchette microtubules are uniformly oriented (Line 114, Fig 1e/f), requires more data to be shown, since this can only true if all microtubules in each field of view clearly have the same protofilament skew.
The MT polarity analysis was performed on all tomograms and the images in Fig. 1f & 1g are only examples. We analyzed 25 tomograms and all MTs in a field of view had the same polarity. This information is now given in the text. Lines 124-129.

Minor points
1 - Figure 2a/b depicts images and quantification plus and minus ends but it seems the color coding is wrong for splayed/curved ends (according to the pie chart, pink should be splayed, green curved but in annotated images I would describe pink as curved and green as splayed).
Due to this confusion, we have changed the MTs ends nomenclature. Following Gudimchuk and McIntosh 2021 Nat Rev, we use 'flared' for MTs where the PFs are about the same length but are not straight and 'tapered' for MT that have a long extension of PFs.

2 - There is a discrepancy in the number of microtubules analyzed for their end morphology and polarity. In the text, it is states the protofilament number of 358 total microtubules were analyzed (line 114). 102 are at the PNR and 139 at caudal side (241 total). The remaining 117 are unaccounted for. In the end morphology quantification (Figure 2), 129 plus-ends are identified and 145 minus ends (274 total). This 33 more ends than in the polarity determination. To give confidence in how the results were obtained, how these values were obtained should be clarified (including number of tomograms, number of microtubules present in each, if any were excluded from analysis/for what reason).

To clarify, to determine the MT polarity in a tomogram, we do not need to see the end (plus or minus), it is based on protofilament slew, thus the access of 117 is coming from tomograms in the center of the manchette.

To determine MT end morphology, we could use more ends than for the polarity measurements. The reason is that some end MTs were not long enough to undergo rotational averaging and to determine polarity, but the end morphology could be determined as it was in the tomogram. We have added the information on how many tomograms contributed for each of the measurements to clarify this point. Lines 124-129.

3 - The overall resolution is reported as 3.4 Å but the resolution of the region containing SH3D21 is low (at least below 6 Å but the scale in Supplementary Figure 1 is cut off so this information is not provided in the manuscript). The local resolution range for each component in the structure (Tubulins, SPACA9, SH3D21) should be provided so that readers can easily assess the level of confidence of protein identification.

As requested, we have increased the resolution range in Fig S2b (it is now 3-7Å). And as you can see in the new figure, most of SH3D21 is below 6. We now also provide the resolution range in the text for each component: 'SPACA9 (local resolution 3 - 5 Å)' …' the protein density binding between PF1 and PF13 (local resolution 4 – 6 Å),…'. Lines: 153-155.

4 - The claim that SH3D21 induces high curvature at the seam (line 188) would be strengthened by comparing interprotofilament angles between manchette microtubules and sperm tail singlets (Fig. 5c). Currently, it remains possible that SPACA9 contributes to curvature in other regions.

Following the reviewer's suggestion, we have measured the rotational angle for sperm tail singlet as well. The results are provided in the revised Fig. 5c. As expected, the interprotofilament angles in the SPACA9 decorated sperm singlet are close to the average of 27.69, and the angle at the seam is only slightly higher (29.6 degrees compared to 41 degrees for the manchette MTs).

5 - Line 208 states "To the best of our knowledge, this is the first observation of a seam binding MIP." This is misleading, as seam-binding MIPs have been described in axonemal structures. The claim should be qualified (e.g., "outside axonemal microtubules").

We have added the disclaimer suggested by the reviewer throughout the manuscript.

6 - For Supplementary Fig 3 – the blue coloring for the SH3 domain is not clearly visible. A color with higher contrast and/or larger images should be provided.

We agree with the reviewer and have changed the color of the SH3D21 domain to magenta for better contrast. (revised Figure S5)

7 - The mask displayed in PDB validation report appears to be a hard-edge cylinder – is this the correct mask used for final refinement? Overall the description of single particle processing would benefit from a Supplementary Figure to show intermediate classes obtained and masks used.

We have added a supplementary figure showing the workflow, including the classes obtained and the masks used (new Fig S1). We don't know why the mask in the validation report appears to be a hard-edged cylinder. The mask used was a soft-edged mask with a soft padding width of 6 pixels.

8 - Acquisition of six datasets acquired on both on both 200 keV and 300 keV microscopes are described in the methods (three sets of tomograms; line 365, three single particle datasets; line 389) but only one is listed in Supplementary Table 1. All electron microscopy data should be listed in the table and how data were merged should be described.

To clarify, we only used 300 kV data for the single particle analysis of the microtubules. We clarify this in the materials and methods section. Line 415

Dear Tzviya,

Thank you for submitting a revised version of your manuscript to The EMBO journal. I sincerely apologise for the delay in the assessment process for your manuscript. Your study has now been seen by all original referees, who find that their previous concerns have been sufficiently addressed and now recommend publication of the manuscript after minor revisions.

In addition to the remaining reviewer requests, there are a few editorial points that need to be addressed before I can extend the official acceptance of the manuscript:

1. Please note that it is The EMBO Journal policy for the transcript of the editorial process (containing referee reports and your response letters) to be published as an online supplement to each paper. If you should prefer removal of any referee-only figures included in the point-by-point response(s), e.g. because they may still be used for future publication or because they have been reproduced from published work by others, please do let us know immediately via response email.
More information is available here: https://www.embopress.org/transparent-process#Review_Process
2. At EMBO Press we ask authors to provide source data for the main manuscript figures. You will receive a separate email with instructions for providing source data with your revised manuscript, including how to upload and organise the files.
3. Please submit a complete author checklist, which you can download from our author guidelines (https://media.springernature.com/original/springer-cms/rest/v1/content/27825796/data/v1). Please insert information in the checklist that is also reflected in the manuscript. The completed author checklist will also be part of the Review Process File.
4. The headings and order of the sections in the manuscript text should be as follows: Abstract / Introduction / Results / Discussion / Methods / Data Availability / Acknowledgements / Disclosure and Competing Interests Statement / References / Figure Legends / Expanded View Figure Legends.
5. Please remove the figures from the manuscript text and upload them as separate high-resolution figure files. Please move the legends to the end of the manuscript text. The supplementary figures should also be removed and uploaded as separate high-resolution files, renamed Figure EV1 - EV6, and their legends should be placed after the main figure legends under the title "Expanded View figure legends".
6. Please check that the funding information is correct and identical both in the manuscript and our online system. Currently, Marie Sklodowska-Curie grant project 101153345 and Dutch Research Council (NWO) grant OCENW.XL21.XL21.048 are missing in the online system.
7. Primary datasets produced in this study need to be deposited in an appropriate public database and resolvable links to the datasets listed in the Data Availability section at the end of Methods. More information about the format of this section can be found here: https://link.springer.com/partners/embo-press/editorial-policies#Data%20availability%20statement.
8. CRediT has replaced the traditional author contributions section because it offers a systematic, machine-readable author contributions format that allows for more effective research assessment. Please remove the Authors Contributions from the manuscript and use the free text boxes beneath each contributing author's name in our online submission system to add specific details on the author's contribution. More information is available in our guide to authors.
9. Please update references according to The EMBO Journal style - the order should be alphabetical. Where there are more than 10 authors on a paper, the first 10 should be listed, followed by 'et al.'
10. Please download and fill our Reagents and Tools Table template (.docx), which you can find in our author guidelines: https://media.springernature.com/original/springer-cms/rest/v1/content/27825802/data/v1
When submitting your revised manuscript, please do not include the Reagents and Tools Table in the Methods section of the manuscript but upload it as a separate file choosing the file type "Reagent Table". Some of the information currently provided Supplementary Table 1 could be included in the Reagents and Tools Table in the section "Other" as instrumentation.
11. Please remove Supplementary Table 1 from the manuscript text, rename it "Table EV1" and upload it as a separate file.
12. Rename the movie files Movie EV1 - Movie EV4. Place each movie legend in a simple .txt README file or in a word document and zip each legend with the corresponding movie file before uploading in our system.
13. Please note that movie files should be mentioned in a sequential order in the manuscript text. Currently, movie S1 (movie EV1) is called out after movies S2-S4.
14. During our routine image integrity checks, we observed that in Figure 3D, top line nucleus and merge panels appear to have a portion removed as seen with appropriate filters. Please check and clarify.
15. Papers published in The EMBO Journal are accompanied online by a 'Synopsis' to enhance discoverability of the manuscript. It consists of A) a short (1-2 sentences) summary of the findings and their significance, B) 3-4 bullet points highlighting key results (the highlights currently included in the manuscript can be used here) and C) a synopsis image that is 550x300-600 pixels large (width x height, jpeg or png format). You can either show a model or key data in the synopsis image. Please note that the image size is rather small and that text needs to be readable at the final size.

Please let me know if you have any questions regarding any of these points. You can use the link below to upload the revised files.

Thank you again for giving us the chance to consider your manuscript for The EMBO Journal. I look forward to receiving the final version.

With kind regards,

Ieva

Ieva Gailite, PhD
Senior Scientific Editor
The EMBO Journal
Meyerhofstrasse 1
D-69117 Heidelberg
Tel: +4962218891309
i.gailite@embojournal.org

We realize that it is difficult to revise to a specific deadline. In the interest of protecting the conceptual advance provided by the work, we recommend a revision within 3 months (21st Jul 2026). Please discuss the revision progress ahead of this time with the editor if you require more time to complete the revisions.

Referee #1:

The authors adequately addressed comments, and I think the additional data (while toning down some of the mechanistic claims) greatly improved the manuscript. The comments below are only for minor improvements or additional insights that would be helpful for the field.

1. It was noted that Taxol normally augments the spacing of the microtubule lattice, yet the spacing of the manchette microtubule lattice is compact. Nothing is discussed with respect to the binding of Taxol (shown in Supplemental Figure 2D). Was binding different from what was expected?
2. It seems that given pulldown assays are established and beautifully demonstrate an interaction between SPACA9 and SH3D21, why was a mutant of residue N119 (Figure 4C) not made? This would support the authors' idea that this conserved residue mediates an interaction.
3. Lines 102-111 is a repeat of lines 94-102.
4. Line 159-shouldn't there be a reference for the rat manchette proteome? Or is there mass spec data in this manuscript? The proteome is also mentioned in supplemental Figure 4.
5. The green arrowheads in Figure 1f-g are difficult to read.
6. Supplemental Figure 6: It seems like there are still microtubules in cells that are not expressing SPACA9 despite the use of nocodazole. Perhaps microtubules are indeed absent and insets with a zoomed-in view would help to see that. Alternatively, are those cells actually expressing SPACA9, just at low levels? Lastly, the size of the scale bars does not appear to be noted.

Referee #2:

The authors have addressed my previous major concerns. The manuscript should be suitable for publication pending corrections of the following points:

Line 23: typo at "microtubule(MT)-based..."

Line 102: something doesn't make sense with "lattice.preparations...." up until line 104.

Line 127-128: "...had their plus-end" is not grammatically sound. Please reword.

Line 132: "Most plus ends..." plus-end vs plus end, the authors aren't consistent. Same for minus ends on line 223.

Line 140: "-TIP..." is not defined.

Line 146: It might be more precise to replace "high-resolution imaging" with "Cryo-ET" (if applicable)

Line 165: "plDDT" should be pLDDT and is also not defined. "PAE" and "STED" as well.

Line 180: "clear density for a hydrogen bond..." The authors cannot claim this at the resolutions reported. Please reword or remove.

Line 182: These should be referred to as Figs. S5a and S5b.

Line 185: It is not clear what Fig. S5 is referring to in this sentence.

The order of lines 186-201 and 202-213 should be swapped. Lines 202-213 present experimental evidence validating the SPACA9-SH3D21 model, whereas lines 186-201 analyze the complex assuming the model is correct. Logically, the validation evidence should precede the functional analysis.

Line 191: "elliptic..." I think the authors mean more elliptical?

Line 198: This should be Fig. 5c.

Line 224: Is there no published manchette proteomics data that can be cited here?

Are the manchettes MTs really that dynamic? Have turnover rates been measured & reported? This is confusing.

Line 226: Do the authors mean knock-out of CLASP2? Unclear.

Line 239: I suggest removing terms like "highly interesting" from a scientific manuscript.

Line 240: commas after mammals and metazoans should be removed

Lines 240-242: Did the authors consider alternative splicing variants? For instance, the Human Protein Atlas indicates that human SH3D21 is ubiquitously expressed and has multiple isoforms. The AlphaFold model in Fig. S5c appears to correspond to isoform AF-A4FU49-6-F1, which contains three SH3 domains, whereas others (e.g., AF-A4FU49-F1) contain only one. The authors should incorporate the potential impact of these variants, particularly the possibility of germline-specific alternative splicing into discussion.

Line 790: Each mention of a PDB ID should be cited according to the databases recommendations.

Referee #3:

This manuscript focusses on the mechanism of stability for microtubules within the manchette, a subcellular structure which forms during spermiogenesis and is critical for nuclear reshaping. The authors combine immunofluorescence, structural (single particle cryo-EM and cryo-ET) and biochemical analysis to identify a complex of SPACA9 and SH3D21 which binds in the microtubule lumen. They also analyse the ends of the manchette microtubules and suggest that CLASP2 plays a role in regulating their morphology and stability.

Overall, the manuscript has been significantly strengthened by the additional experiments performed. Specifically, the new data in Suppl. Fig 6 show that SPACA9 and SH3D21 colocalise in cells and bind microtubules and that SPACA9 or SPACA9/SH3D21 but not SH3D21 alone leads to increased microtubule stability. This provides valuable insight into the function of the protein complex they identify bound to the manchette. The authors also validate that SPACA9/SH3D21 interacts in cells, through co-IP. The inclusion of more data/information relating to the alphafold structure predictions and single particle analysis workflows also provides confidence in identification of these components and overall this part of the manuscript is convincing.

Regarding the appearance and regulation of microtubule ends, the description of cryo-ET data analysis and discussion of what was known previously known (lines 120-129) is improved. The new data in Fig 2c, showing the relative position of the minus-end binding CAMSAP1, which can now be compared to that of the plus-end binding CLIP-170 and CLASP2 is valuable. This data shows that the localisation of each component is distinct, with CLIP-170 at the perinuclear ring, CAMSAP1 at the minus ends which are of variable length, and CLASP2 evenly distributed across the manchette. The manuscript has been re-written to reflect previous concerns about the conclusions relating to CLASP2. This has improved the accuracy of the claims in the results but should be discussed more carefully to reduce confusion. My suggestions are outlined below.

Overall, the question of how manchette microtubules are stabilised is important to address due to its implications in male fertility. This study identifies SPACA9/SH3D21 as proteins which bind the manchette, characterises their interaction and shows they can act to stabilise microtubules in cells. This study provides a vital platform for future work into how factors which control manchette stability are regulated throughout the spermatogenesis process.

Major points:

1) Line 224 states that CLASPs bind to both plus and minus ends and the data presented in the manuscript are in agreement with this. But the data show additional binding along the microtubule lattice. Since there is no mention of CLASP2 in the introduction, whether this lattice binding is novel and what role it might have is not clearly stated. This should be explicitly discussed, for example by including the information provided in response to reviewer 2, point 5 here.
2) It should be made clear that the claims in lines 260-263 are possible mechanisms rather than proven conclusions. The idea that SPACA9/SH3D21 prevents seam opening is discussed clearly in the sections above but the suggested mode/mechanism of action for CLASP2 needs more detail.

Minor points:
1) End of introduction (lines 102-111) is duplicated.
2) In Supplementary Figure 1, the number of particles in each class selected during cryo-EM processing should be provided (not only in the final classes). This is important so that readers can easily understand how many particles were required at each step to obtain the classification results.
3) Please include the definition of blunt, flared and tapered end morphology (explained in response to reviewer 2, minor point 1) in the methods and cite the Gudimchuk and McIntosh 2021 Nat Rev paper as the basis for this.
4) In Supplementary Figure 6, the ladder for the co-IP should be fully labelled so we can interpret the additional bands at the bottom properly. I would suggest putting this on the left and indicating positions of SH3D21/SPACA9 on the right.

EMBOJ-2026-123987-T

Referee #1 (Report for Author)

1.It was noted that Taxol normally augments the spacing of the microtubule lattice, yet the spacing of the manchette microtubule lattice is compact. Nothing is discussed with respect to the binding of Taxol (shown in Supplemental Figure 2D). Was binding different from what was expected?
There are no differences in local Taxol binding between our structure and those of previously published structures. We added this observation to the results as: "…, while the binding mode of paclitaxel is similar to the previously reported one (Fig. EV2D) (Prota et al, 2023)".

2. It seems that given pulldown assays are established and beautifully demonstrate an interaction between SPACA9 and SH3D21, why was a mutant of residue N119 (Figure 4C) not made? This would support the authors' idea that this conserved residue mediates an interaction.
We thank the reviewer for this suggestion. We feel that further mutagenesis experiments go beyond the scope of this study.

3. Lines 102-111 is a repeat of lines 94-102.
We removed this duplicated paragraph.

4. Line 159-shouldn't there be a reference for the rat manchette proteome? Or is there mass spec data in this manuscript? The proteome is also mentioned in supplemental Figure 4.
We added the reference for the rat proteome to both sentences.

5. The green arrowheads in Figure 1f-g are difficult to read.
We changed the arrowheads to one large arrowhead, indicating skew, next to the rotational averages.

6. Supplemental Figure 6: It seems like there are still microtubules in cells that are not expressing SPACA9 despite the use of nocodazole. Perhaps microtubules are indeed absent and insets with a zoomed-in view would help to see that. Alternatively, are those cells actually expressing SPACA9, just at low levels? Lastly, the size of the scale bars does not appear to be noted.
We agree that in cells that are not expressing SPACA9, even upon treatment with Nocodazole, some MTs are not depolymerized. This is most likely due to other microtubule-associated proteins stabilizing a subfraction of MTs in the cells, and at the tested concentrations, Nocodazole will never fully depolymerized 100% of MTs in cells.
Scale bar sizes were added to the figure legends.

Referee #2 (Report for Author)

Line 23: typo at "microtubule(MT)-based..."
The typo was corrected

Line 102: something doesn't make sense with "lattice.preparations...." up until line 104.
This was corrected

Line 127-128: "...had their plus-end" is not grammatically sound. Please reword.
We reworded this sentence to "100% (102/102 in 8 tomograms) of MT plus ends faced toward the perinuclear ring (Fig. 1F)."

Line 132: "Most plus ends..." plus-end vs plus end, the authors aren't consistent. Same for minus ends on line 223.
We changed the text, and now consistently refer to "plus ends" and "minus ends".

Line 140: "-TIP..." is not defined.
TIP is now defined as minus end tracking protein.

Line 146: It might be more precise to replace "high-resolution imaging" with "Cryo-ET" (if applicable)
We agree with this and reworded to Cryo-ET.

Line 165: "plDDT" should be pLDDT and is also not defined. "PAE" and "STED" as well.
We defined these abbreviations.

Line 180: "clear density for a hydrogen bond..." The authors cannot claim this at the resolutions reported. Please reword or remove.
We reworded this sentence. It now reads "…revealed a density suggesting a hydrogen bond …"

Line 182: These should be referred to as Figs. S5a and S5b.
We corrected these figure references.

Line 185: It is not clear what Fig. S5 is referring to in this sentence.
We removed the unclear figure reference.

The order of lines 186-201 and 202-213 should be swapped. Lines 202-213 present experimental evidence validating the SPACA9-SH3D21 model, whereas lines 186-201 analyze the complex assuming the model is correct. Logically, the validation evidence should precede the functional analysis.
We agree with this point and have swapped the paragraphs.

Line 191: "elliptic..." I think the authors mean more elliptical?
We corrected this typo.

Line 198: This should be Fig. 5c.
We corrected the wrong figure reference.

Line 224: Is there no published manchette proteomics data that can be cited here?
We cited published manchette proteomics in the relevant sentences throughout the manuscript and figure descriptions.

Are the manchettes MTs really that dynamic? Have turnover rates been measured & reported? This is confusing.
Manchette MTs are not dynamic like cytosolic MT; they retain structural integrity over several days during spermiogenesis (Introduction (Dunleavy et al, 2019a; Lehti & Sironen, 2016; Mochida et al, 1998)).

Line 226: Do the authors mean knock-out of CLASP2? Unclear.
Indeed, we mean a CLASP2 knock-out. We clarified this sentence, it now reads 'Characterization of manchettes in CLASP2 knock-out mice will help to further understand CLASP2 function in the manchette and during spermiogenesis'.

Line 239: I suggest removing terms like "highly interesting" from a scientific manuscript.
We reworded "highly interesting" to "Future studies on the interaction of the external region of SH3D21 with other proteins or the disordered tails of MT with cross-linking mass spectrometry can clarify its role ."

Line 240: commas after mammals and metazoans should be removed
We removed the comma.

Lines 240-242: Did the authors consider alternative splicing variants? For instance, the Human Protein Atlas indicates that human SH3D21 is ubiquitously expressed and has multiple isoforms. The AlphaFold model in Fig. S5c appears to correspond to isoform AF-A4FU49-6-F1, which contains three SH3 domains, whereas others (e.g., AF-A4FU49-F1) contain only one. The authors should incorporate the potential impact of these variants, particularly the possibility of germline-specific alternative splicing into discussion.
Regarding splicing forms: In the rat, there are no other splicing forms of the protein, and the predicted SH3D21 has only one SH3 domain. However, we agree that multiple isoforms in humans suggest that splicing variants are important to consider as potential mechanisms of MT binding/regulation in spermatids. We have added a short sentence mentioning this to the discussion: "Interestingly, humans have multiple isoforms, with one predicted to have three SH3 domains. Hence, it will be valuable to investigate whether the seam bridge has a different organization in the manchette MTs of non-mammalian organisms and investigate the potential role of spermatid-specific splicing isoforms in humans."
While working on this revision, we also found an error in the panels' order of Fig S5c, which has now been corrected.

Line 790: Each mention of a PDB ID should be cited according to the databases recommendations.
We added PDB IDs whenever a protein structure is mentioned.

Referee #3 (Report for Author)

Overall, the question of how manchette microtubules are stabilised is important to address due to its implications in male fertility. This study identifies SPACA9/SH3D21 as proteins which bind the manchette, characterises their interaction and shows they can act to stabilise microtubules in cells. This study provides a vital platform for future work into how factors which control manchette stability are regulated throughout the spermatogenesis process.

Major points:
1) Line 224 states that CLASPs bind to both plus and minus ends and the data presented in the manuscript are in agreement with this. But the data show additional binding along the microtubule lattice. Since there is no mention of CLASP2 in the introduction, whether this lattice binding is novel and what role it might have is not clearly stated. This should be explicitly discussed, for example by including the information provided in response to reviewer 2, point 5 here.
We included a paragraph discussing CLASP2 binding across the whole manchette. It now reads: 'Many MT end-binders can bind along the MT lattice, an effect more pronounced at higher protein concentrations, and this is well-established for CLASPs (Foster et al, 2022; Aher at al, 2018; Lawrence & Zanic, 2019). As CLASP2 binds to both plus and minus ends, and can associate with MT lattice, albeit more weakly, the signal is across the entire manchette.'

2) It should be made clear that the claims in lines 260-263 are possible mechanisms rather than proven conclusions. The idea that SPACA9/SH3D21 prevents seam opening is discussed clearly in the sections above but the suggested mode/mechanism of action for CLASP2 needs more detail.
We do not investigate the mechanism of CLASP2 in this study, but rather reveal its involvement in the manchette. Since the mechanism of MT stabilization via CLASP2 is not fully understood and our study does not provide new mechanistic insights into this, we prefer not to speculate on this in our discussion.

Minor points:
1)End of introduction (lines 102-111) is duplicated.
We removed the duplicated paragraph.

2) In Supplementary Figure 1, the number of particles in each class selected during cryo-EM processing should be provided (not only in the final classes). This is important so that readers can easily understand how many particles were required at each step to obtain the classification results.
We added absolute numbers of each class throughout the 3D classification steps (revised Fig S1).

3) Please include the definition of blunt, flared and tapered end morphology (explained in response to reviewer 2, minor point 1) in the methods and cite the Gudimchuk and McIntosh 2021 Nat Rev paper as the basis for this.
We included this in the materials and methods section and added the corresponding reference. It now reads: 'MT end structures were assigned based on previous literature (Gudimchuk & McIntosh, 2021). In short, blunt ends are straight ends with all PFs roughly the same length. Flared MT ends are where PFs are about the same length but not straight. Tapered ends have a long extension of a few PFs.'

4) In Supplementary Figure 6, the ladder for the co-IP should be fully labelled so we can interpret the additional bands at the bottom properly. I would suggest putting this on the left and indicating positions of SH3D21/SPACA9 on the right.
We added labels for the molecular weight ladder to the figure.

Dear Tzviya,

I sincerely apologise for the delays in the processing of your manuscript caused by conference travel and holidays here in Southern Germany. Thank you for incorporating the final editorial and formatting requests in the manuscript. I am now pleased to inform you that your manuscript has been accepted for publication. Congratulations with a nice study!

Before we forward your manuscript to our publishers, there are a few of aspects that I still need your input on:
1. We would like to propose some edits in the manuscript title, abstract and synopsis. We have also selected a short blurb that will accompany the title of your manuscript in our online table of contents. Please take a look at the proposed text changes in the attached text file - any corrections or edits from your side are very welcome.
2. With the changes in our website, the dimensions of the synopsis image that we can incorporate have changed to 550 x 300 pixels width/height. I have selected a section of the synopsis image that you kindly provided that would approximately fit these specifications. Please let me know if this attached version would be acceptable.
3. Latest at the proofs stage, please add resolvable links to the datasets in the Data Availability section - i.e., links that lead directly to the dataset rather than the database in general.

Once I have received your input on these points, I will be happy to forward your manuscript to our publishers for typesetting.

Your manuscript will be processed for publication by EMBO Press. It will be copy edited and you will receive page proofs prior to publication. Please note that you will be contacted by Springer Nature Author Services to complete licensing and payment information.

Please note that it is The EMBO Journal policy for the transcript of the editorial process (containing referee reports and your response letters) to be published as an online supplement to each paper. If you should prefer removal of any referee-only figures included in the point-by-point response(s), e.g. because they may still be used for future publication or because they have been reproduced from published work by others, please do let us know immediately via response email.
More information is available here: https://link.springer.com/partners/embo-press/editorial-policies#Peer%20review

You may qualify for financial assistance for your publication charges - either via a Springer Nature fully open access agreement or an EMBO initiative. Check your eligibility: https://link.springer.com/journal/44318/how-to-publish-with-us

Should you be planning a Press Release on your article, please notify the production team via this form in order to coordinate publication and release dates: https://forms.cloud.microsoft/e/eH4A8JVP6g

I look forward to hearing from you soon on the final textual polishes. Thank you for your contribution to The EMBO Journal!

Best wishes,

Ieva

Ieva Gailite, PhD
Senior Scientific Editor
The EMBO Journal
Meyerhofstrasse 1
D-69117 Heidelberg
Tel: +4962218891309
i.gailite@embojournal.org

EMBO Press Author Checklist

Corresponding Author Name: Tzviya Zeev Ben Mordehai	
Journal Submitted to: The EMBO Journal	
Manuscript Number: EMBOJ-2026-123987R	

USEFUL LINKS FOR COMPLETING THIS FORM
The EMBO Journal - Author Guidelines
EMBO Reports - Author Guidelines
Molecular Systems Biology - Author Guidelines
EMBO Molecular Medicine - Author Guidelines

Reporting Checklist for Life Science Articles (updated January
This checklist is adapted from Materials Design Analysis Reporting (MDAR) Checklist for Authors. MDAR establishes a minimum set of requirements in transparent reporting in the life sciences (see Statement of Task: 10.31222/osf.io/9sm4x). Please follow the journal's guidelines in preparing your
Please note that a copy of this checklist will be published alongside your article.

Abridged guidelines for figures
1. Data
The data shown in figures should satisfy the following conditions:
- the data were obtained and processed according to the field's best practice and are presented to reflect the results of the experiments in an accurate and unbiased manner.
- ideally, figure panels should include only measurements that are directly comparable to each other and obtained with the same assay.
- plots include clearly labeled error bars for independent experiments and sample sizes. Unless justified, error bars should not be shown for technical
- if n<5, the individual data points from each experiment should be plotted. Any statistical test employed should be justified.
- Source Data should be included to report the data underlying figures according to the guidelines set out in the authorship guidelines on Data

2. Captions
Each figure caption should contain the following information, for each panel where they are relevant:
- a specification of the experimental system investigated (eg cell line, species name).
- the assay(s) and method(s) used to carry out the reported observations and measurements.
- an explicit mention of the biological and chemical entity(ies) that are being measured.
- an explicit mention of the biological and chemical entity(ies) that are altered/varied/perturbed in a controlled manner.
- the exact sample size (n) for each experimental group/condition, given as a number, not a range;
- a description of the sample collection allowing the reader to understand whether the samples represent technical or biological replicates (including how many animals, litters, cultures, etc.).
- a statement of how many times the experiment shown was independently replicated in the laboratory.
- definitions of statistical methods and measures:
  - common tests, such as t-test (please specify whether paired vs. unpaired), simple χ2 tests, Wilcoxon and Mann-Whitney tests, can be unambiguously identified by name only, but more complex techniques should be described in the methods section;
  - are tests one-sided or two-sided?
  - are there adjustments for multiple comparisons?
  - exact statistical test results, e.g., P values = x but not P values < x;
  - definition of 'center values' as median or average;
  - definition of error bars as s.d. or s.e.m.

Please complete ALL of the questions below.
Select "Not Applicable" only when the requested information is not relevant for your study.

Materials

Newly Created Materials	Information included in the manuscript?	In which section is the information available? (Reagents and Tools Table, Materials and Methods, Figures, Data Availability Section)
New materials and reagents need to be available; do any restrictions apply?	Not Applicable	

Antibodies	Information included in the manuscript?	In which section is the information available? (Reagents and Tools Table, Materials and Methods, Figures, Data Availability Section)
For **antibodies** provide the following information:<br>- Commercial antibodies: RRID (if possible) or supplier name, catalogue number and or/clone number<br>- Non-commercial: RRID or citation	Yes	Reagents and Tools Table

DNA and RNA sequences	Information included in the manuscript?	In which section is the information available? (Reagents and Tools Table, Materials and Methods, Figures, Data Availability Section)
**Short novel DNA or RNA including primers, probes**: provide the sequences.	Yes	Reagents and Tools Table

Cell materials	Information included in the manuscript?	In which section is the information available? (Reagents and Tools Table, Materials and Methods, Figures, Data Availability Section)
**Cell lines:** Provide species information, strain. Provide accession number in repository **OR** supplier name, catalog number, clone number, and/**OR** RRID.	Yes	Reagents and Tools Table
**Primary cultures:** Provide species, strain, sex of origin, genetic modification status.	Not Applicable	
Report if the cell lines were recently **authenticated** (e.g., by STR profiling) and tested for mycoplasma contamination.	Not Applicable	

Experimental animals	Information included in the manuscript?	In which section is the information available? (Reagents and Tools Table, Materials and Methods, Figures, Data Availability Section)
**Laboratory animals or Model organisms:** Provide species, strain, sex, age, genetic modification status. Provide accession number in repository **OR** supplier name, catalog number, clone number, **OR** RRID.	Yes	Reagents and Tools Table
**Animal observed in or captured from the field:** Provide species, sex, and age where possible.	Not Applicable	
Please detail **housing and husbandry conditions**.	Not Applicable	

Plants and microbes	Information included in the manuscript?	In which section is the information available? (Reagents and Tools Table, Materials and Methods, Figures, Data Availability Section)
**Plants:** provide species and strain, ecotype and cultivar where relevant, unique accession number if available, and source (including location for collected wild specimens).	Not Applicable	
**Microbes:** provide species and strain, unique accession number if available, and source.	Not Applicable	

Human research participants	Information included in the manuscript?	In which section is the information available? (Reagents and Tools Table, Materials and Methods, Figures, Data Availability Section)
If collected and within the bounds of privacy constraints report on age, sex and gender or ethnicity for all study participants.	Not Applicable	

Core facilities	Information included in the manuscript?	In which section is the information available? (Reagents and Tools Table, Materials and Methods, Figures, Data Availability Section)
If your work benefited from core facilities, was their service mentioned in the acknowledgments section?	Yes	Acknowledgements

Design

Study protocol	Information included in the manuscript?	In which section is the information available? (Reagents and Tools Table, Materials and Methods, Figures, Data Availability Section)
If study protocol has been **pre-registered, provide DOI in the manuscript**. For clinical trials, provide the trial registration number **OR** cite DOI.	Not Applicable	
Report the **clinical trial registration number** (at ClinicalTrials.gov or equivalent), where applicable.	Not Applicable	

Laboratory protocol	Information included in the manuscript?	In which section is the information available? (Reagents and Tools Table, Materials and Methods, Figures, Data Availability Section)
Provide DOI OR other citation details if **external detailed step-by-step protocols** are available.	Not Applicable	

Experimental study design and statistics	Information included in the manuscript?	In which section is the information available? (Reagents and Tools Table, Materials and Methods, Figures, Data Availability Section)
Include a statement about **sample size** estimate even if no statistical methods were used.	Yes	Materials and Methods
Were any steps taken to minimize the effects of subjective bias when allocating animals/samples to treatment (e.g. **randomization procedure**)? If yes, have they been described?	Not Applicable	
Include a statement about **blinding** even if no blinding was done.	Not Applicable	
Describe **inclusion/exclusion criteria** if samples or animals were excluded from the analysis. Were the criteria pre-established?<br><br>If sample or data points were omitted from analysis, report if this was due to attrition or intentional exclusion and provide justification.	Not Applicable	
For every figure, are **statistical tests** justified as appropriate? Do the data meet the assumptions of the tests (e.g., normal distribution)? Describe any methods used to assess it. Is there an estimate of variation within each group of data? Is the variance similar between the groups that are being statistically compared?	Not Applicable	

Sample definition and in-laboratory replication	Information included in the manuscript?	In which section is the information available? (Reagents and Tools Table, Materials and Methods, Figures, Data Availability Section)
In the figure legends: state number of times the experiment was **replicated** in laboratory.	Not Applicable	
In the figure legends: define whether data describe **technical or biological replicates**.	Not Applicable	

Ethics

Ethics	Information included in the manuscript?	In which section is the information available? (Reagents and Tools Table, Materials and Methods, Figures, Data Availability Section)
Studies involving **human participants**: State details of **authority granting ethics approval** (IRB or equivalent committee(s), provide reference number for approval.	Not Applicable	
Studies involving **human participants**: Include a statement confirming that **informed consent** was obtained from all subjects and that the experiments conformed to the principles set out in the WMA Declaration of Helsinki and the Department of Health and Human Services Belmont Report.	Not Applicable	
Studies involving **human participants: For** publication of **patient photos**, include a statement confirming that consent to publish was obtained.	Not Applicable	
Studies involving experimental **animals**: State details of **authority granting ethics approval** (IRB or equivalent committee(s), provide reference number for approval. Include a statement of compliance with ethical regulations.	Not Applicable	
Studies involving **specimen and field samples:** State if relevant **permits** obtained, provide details of authority approving study; if none were required, explain why.	Not Applicable	

Dual Use Research of Concern (DURC)	Information included in the manuscript?	In which section is the information available? (Reagents and Tools Table, Materials and Methods, Figures, Data Availability Section)
Could your study fall under dual use research restrictions? Please check biosecurity documents and list of **select agents and toxins** (CDC): https://www.selectagents.gov/sat/list.htm	Not Applicable	
If you used a select agent, is the security level of the lab appropriate and reported in the manuscript?	Not Applicable	
If a study is subject to dual use research of concern regulations, is the name of the **authority granting approval and reference number** for the regulatory approval provided in the manuscript?	Not Applicable	

Reporting

The MDAR framework recommends adoption of discipline-specific guidelines, established and endorsed through community initiatives. Journals have their own policy about requiring specific guidelines and recommendations to complement MDAR.

Adherence to community standards	Information included in the manuscript?	In which section is the information available? (Reagents and Tools Table, Materials and Methods, Figures, Data Availability Section)
State if relevant guidelines or checklists (e.g., **ICMJE, MIBBI, ARRIVE, PRISMA**) have been followed or provided.	Not Applicable	
For **tumor marker prognostic studies,** we recommend that you follow the **REMARK** reporting guidelines (see link list at top right). See author guidelines, under 'Reporting Guidelines'. Please confirm you have followed these guidelines.	Not Applicable	
For **phase II and III randomized controlled trials**, please refer to the **CONSORT** flow diagram (see link list at top right) and submit the CONSORT checklist (see link list at top right) with your submission. See author guidelines, under 'Reporting Guidelines'. Please confirm you have submitted this list.	Not Applicable	

Data Availability

Data availability	Information included in the manuscript?	In which section is the information available? (Reagents and Tools Table, Materials and Methods, Figures, Data Availability Section)
Have **primary datasets** been deposited according to the journal's guidelines (see 'Data Deposition' section) and the respective accession numbers provided in the Data Availability Section?	Not Applicable	
Were **human clinical and genomic datasets** deposited in a public access-controlled repository in accordance to ethical obligations to the patients and to the applicable consent agreement?	Not Applicable	
Are **computational models** that are central and integral to a study available without restrictions in a machine-readable form? Were the relevant accession numbers or links provided?	Yes	Data Availibility
If publicly available data were reused, provide the respective **data citations in the reference list**.	Yes	References
